# A qualitative study of drivers of psychoactive substance use among Mekelle University students, Northern Ethiopia

**DOI:** 10.1186/s13011-018-0190-1

**Published:** 2019-03-04

**Authors:** Znabu Hadush Kahsay, Azeb Gebresilassie Tesema, Alessandra N. Bazzano

**Affiliations:** 1 Health education and behavioral science Unit, School of public health, Mekelle Univerity, POB:1871, Mekelle University, Mekelle City, Ethiopia; 2 Global Community Health and Behavioral Sciences, Tulane University School of Public Health, New Orleans, LA 70125 USA

**Keywords:** Motivating factors, Substance use, Mekelle University, Ethiopia

## Abstract

**Background:**

Psychoactive substance (PS) use is a public health concern among University students. Understanding the factors underlay the use helps to underpin effective preventive interventions. However, there is dearth of exploratory studies on the drivers of psychoactive substance use in Ethiopian universities. Here, we aimed to explore the drivers for psychoactive substance use among Mekelle University undergraduate students.

**Methods:**

Exploratory qualitative study was conducted from April 1 to May 30, 2017. We conducted five focus group discussions (FGDs) and eleven in-depth interviews (IDIs) with students, proctors and bar owners. We also conducted three round interviews within two weeks interval with each of four key-informant researchers (KIR). Participants were selected purposively and the investigators conducted the discussions and the interviews using semi-structured guides. Data were audio-recorded, transcribed verbatim and imported into qualitative data analysis software for coding and analysis. An inductive approach was applied to crystalize non-repetitive emerging themes overarching the drivers for psychoactive substance use among university students.

**Results:**

The following themes emerged as drivers for psychoactive substance use among University students; (1)feeling helpless following detachment from family, (2) prior experience with substances, (3) socialization reasons, (4) low academic performance, (5) physical environment (explained by easy access to substance and limited recreational alternatives), and (6) sub-optimal organizational support.

**Conclusions:**

Multiple drivers that range from individual to structural levels are involved in university student’s use of psychoactive substances, with socialization process at the center of the factors. Thus, the study appeals for a range of multifaceted interventions directed to the individual, interpersonal and organizational level factors.

**Electronic supplementary material:**

The online version of this article (10.1186/s13011-018-0190-1) contains supplementary material, which is available to authorized users.

## Background

Psychoactive substance (PS) use, such as drinking alcohol, cigarette smoking, khat chewing, cannabis use, and use of illicit drugs, is a public health concern among youth worldwide (1). Its negative consequences including academic failure, risk behaviors including risky sexual behavior, substance use disorders, and disability, become major threats for the youth population including university students [1, 2].

Literature shows prevalent substance use among college students [3–5], including those attending universities in low and middle income countries such as Ethiopia [6–11]. In Ethiopia, the youth demographic aged 15–29 years accounts for 28% of the total population [12–14] and the number of young people joining Universities is increasing over time. Students from different areas of the country where substance use may be prevalent attend Ethiopian universities for higher education. Among Ethiopian Men, 46% use alcohol 27% Khat and 4% Tobacco. In Tigray region, where Mekelle University is based, 91% of men surveyed and 71% of women surveyed, consume alcohol [12]. Psychoactive substance use has become a pressing concern among students in Ethiopian Universities, where rates range from 16 to 62% [6, 7, 15]. Students often stay for three to six years on campus to attend higher education programs in Ethiopian universities, which could make them vulnerable to harmful behaviors like PS use. However, the drivers of the use among University students remain poorly documented.

Understanding these factors that drive behavior through association, though not necessarily causation, will be an important step towards designing effective preventive measures. Previous studies exist, but most of those have been quantitative studies. These studies revealed factors related to PS use including peer influence, academic reasons (including wakefulness for reading), recreation, and anxiety management [7, 9, 15–17]. Mass media advertisement of alcohol beverages for example, is also reported as a factor [11, 18–20]. However, the quantitative approach used in previous studies resulted in restricted capacity to explore the range and depth of the these associated factors to use substance among this population. In addition, there is a dearth of evidence or detailed description of the drivers, the context. and how these interact to clarify PS use. This research gap decreases the ability of policy makers and programmers to use existing literature to design effective preventive measures for treatment of youth. The investigators of the current study followed a qualitative approach to facilitate wider and deeper understanding of the drivers to use PS.

Interventions to reduce the health burden of substance use are more effective if programs are backed by contextualized evidence. Thus, the current study aimed at exploring the driving factors for tobacco, alcohol, Khat, cannabis and illicit drugs use, here after collectively referred as PS use, among Mekelle University undergraduate students with the intention to assist in development of future interventions. The inclusion of these specific substances follows the WHO Alcohol, Smoking and Substance involvement (ASSIST) classification found at http://www.who.int/substance_abuse/activities/assist/en/.

## Methods and participants

### Study setting

The study was conducted among undergraduate students attending Mekelle University (MU) from April to May 2017. MU is located in Mekelle town, the capital of Tigray regional state, Ethiopia. In 2018, the University had 26, 371 undergraduate students in six Campuses [21].

The study was part of the baseline assessment for a large scale study on stage specific behavioral therapeutic intervention for PS use among Mekelle University students, Ethiopia. A quantitative study was conducted to determine the prevalence of problematic PS use among MU students followed by a qualitative exploration of the driving factors. Specifically, the current article aimed to explore contextual drivers for PS use among University students.

### Study design

An exploratory qualitative study design was employed to understand the drivers, defined as likely associated factors not proximal causes, for PS use (as previously defined through WHO ASSIST as alcohol, tobacco, khat, cannabis, and illicit drugs) among University students.

### Sample size and sampling procedure

Five focus group discussions (FGDs) (four with substance user students and one with non-users) each with 8–10 participants were conducted. In succession to the FGDs, 11 in-depth interviews (IDIs) were conducted; seven with students (three substance users and four non-users), two with proctors and one with a bar owner. Participants, including proctors of student dormitories and bar owners (around the gates of campuses), were chosen due to the likelihood of having information on University students’ substance use. Participants were selected purposively [22] based on their characteristics and expertise in the subject matter. The investigators also asked participants follow up questions (probes) for issues raised during the discussion/interview that need to be completed, confirmed, clarified. In addition to the FGDs and IDIs, the study also utilized a Key Informant Researchers’ (KIR) approach to better capture drivers for PS use. Four key informant researchers (KIRs), two substance user students and two non-users students, were purposively recruited with the role of providing information, introductions, and interpretation as well as access to observations that an outsider would not normally have [22, 23]. They were trained and oriented to explicitly observe and search out salient views to illustrate the complex webs of driving factors. They used informal discussions and non-participant observations with substance user students, drug vendors, proctors, security/guards, and also conducted observation of evidence of substances previously used in the MU compound to broaden their understanding. Each KIR was interviewed three times within two weeks interval for new insights and understandings in the regard.

Semi-structured guides with open ended questions were used for the FGDs and interviews. The FGDs were conducted first to explore the range of opinions on the subject matter followed by interviews for deeper understandings. Guides were continuously modified as the data collection moves forward to capture emerged issues. FGDs and interviews were conducted in place convenient to the participants where privacy is kept and recording is possible with minimal sound disturbance.

### Trustworthiness

The investigators reflected on and considered their prior conceptions, expectations, and experiences before the study began, to reduce introducing potential bias while they collected, transcribed, coded and analyzed the data. Data from FGDs, interviews with key informants and KIRs were triangulated for similarities and variations. The authors adopted an inductive approach and conducted analysis side to side of the data collection to catch the emerging issues in the succeeding discussions/interviews. Investigators also conducted debriefing sessions on a daily basis.

### Data analysis

Investigators conducted data collection and analysis concurrently. Audiotaped data were listened to repeatedly for comprehension and familiarity, these were transcribed verbatim and imported into Atlas.ti qualitative data analysis software version 7.5 (ATLAS.ti Scientific Software Development GmbH, Berlin, 2015) for coding and analysis. Field notes and investigator memos were also linked to respective files to assist analysis. Independently, two investigators openly coded the data. Then, the two investigators came together to check and discuss the inter-coder reliability. Followig this, similar codes were systematically categorized and unique themes emerged. After themes emerged in first round analysis, the investigators again reviewed transcripts for a second round of analysis aimed to check whether codes and themes were grounded in the data and if important insights and dimensions of each theme were fully recognized.

### Ethical consent

Ethical approval and clearance was obtained from the ethical review board of the College of Health Science at the Research and Community Service office, Mekelle University (approval number 1048/2017). Written consent was sought from each participant.

## Results

### Participant socio-demographic characteristics

The age of the students ranged from 20 to 27 and all were the second year students and above (Table 1). The driving factors emerged were (1) feeling of helplessness following detachment from family, (2) prior experience of substance use, (3) socialization reasons, (4) low academic performance, (5) physical environment, and (6) sub-optimal organizational support (see Table 2).

**Table 1:** Description of participants for vulnerability of psychoactive substance use among Mekelle University Undergraduate Students, Tigray, Ethiopia

FGDs	Purpose of inclusion	Sex	≠of participants	College
FGD 1	Substance non-user student	Male	Nine	Health sciences
FGD 2	Substance user student	Males	Eight	Health sciences
FGD 3	Substance user student	Males	Eight	Business and Economics
FGD 4	Substance user student	Females	Eight	Business and Economics
FGD 5	Substance user student	Males	Eight	Engineering and Technology
Participants in In-depth interview (IDI) and Key informant’s interview(KII) and Key informant researchers(KIR)				
Interviews	Purpose of inclusion	Sex	Age	College
IDI 1	Substance non-user student	Male	23	Law and Governance
IDI 2	Substance user student	Male	21	Business and Economics
IDI 3	Substance user student	Male	27	Business and Economics
IDI 4	Substance user student	Male	21	Business and Economics
IDI 5	Substance Non-user student	Female	20	Health sciences
IDI 6	Substance Non-user student	Female	22	Business and Economics
IDI 7	Substance user student	Male	22	Business and Economics
KII 1	Proctor	Male	27	Health sciences
KII 2	Proctor	Female	24	Health sciences
KII 3	Bar owner	Male	26	–
KII 4	Shop owner	Male	32	–
KIR 1	Substance user student	Male	21	Engineering and Technology
KIR 2	Substance user student	Female	21	Business and Economics
KIR 3	Substance non-user user	Male	22	Business and Economics
KIR 4	Substance non-user user student	Male	21	Health sciences

**Table 2:** Themes and sub-themes on motivating factors for substance use among Mekelle Univeristy students, 2018

Theme 1. Feelings of helplessness following detachment from family	
Theme 2. Prior substance use	
Theme 3. Socialization Reasons	
• Peer pressure	
• *Feelings of inferiority*	
• Poor socialization skill	
• Ceremonies & Festivities	
Theme 4. Physical environment	
• *Easy access to substance use*	
• *Shortage of alternative Entertainments*	
• *Excess free time*	
Theme 5. Sub-optimal Institutional support	
• Administrative related	
• Poor regulation enforcement in University	
• Media and advertisement	
Theme 6.Dissatisfaction with academic conditions	
• *Perceived and actual academic failure*	
• *Factors related to quality Education*	
• *Hopeless in the educational carrier*	

### Theme 1: Feelings of helplessness following detachment from family

Descriptions of feeling helpless following detachment from the family emerged as driver to psychoactive substance (PS) use. It was explained by absence of family support and supervision, as well as general feelings of loneliness. The resulting anxiety appeared to drive students to use PS. The absence of parental supervision linked with feeling helpless and subsequent PS use was captured in the following quote:

*I strongly believe that among the situations that motivate us [students] to use a substance is being out of [sight] watching from family. Unlike in the pre-university, there is no family who would be discouraged or disappointed with my use here in the University"* [21-years-old-student].

Lack of support from the community (including the teachers) was also repeatedly reported as sub-optimal, which left students feeling helpless and lonely.

*In high school, your teacher worries about his student’s future career. He tries to help you to succeed to the edge of your potential. But here [in University], it seems it is not their concern if you are going to be dismissed; it is not surprising to them. Therefore, you feel that there is no one around you: no one is there to help you. Then, you will make the substance as an alternative*. [20-years-old,Female-student].

Absence of parental support also led to mismanagement of pocket money provided by family, which often led them to PS use. Participants repeatedly mentioned that students with relatively greater amount of pocket money often got involved in extensive and poly-substance use.

Participants also frequently mentioned that the University’s administrative body, the student’s union office and the proctors in University rarely offer help to the students, which make them feel helpless. This view was captured in the quote below.

… *After their life gets ruined due to substance use, the worst [problem] on the campus is that there is no care provided to them even the students themselves"* [Male-proctor].

### Theme 2: Prior substance use

Students with previous substance use often continue this use in university. Based on their experience, students tend to socialize with students of similar experience and interest. A KIR stated:

*Based on their experience, students continue substance use. […] If someone were drinking alcohol during pre-University, he looks for a friend who drinks here [in University]. The same is for Khat chewing and tobacco use* [KIR 2].

Participants also mentioned that students coming from areas where substance use is common (e.g. Khat and alcohol) share the community’s positive salient belief that favors that substance use.

*For students who come from areas of the country where Khat chewing is common, Khat could be seen as means of entertainment and socialization* [KIR 2].

Another student also reflected how his parents’ Khat use influenced him to use it in University.

*I was observing my family buying Khat every day at home for consumption. Then I feel there is a reason why they use it. Then I was planning to experiment with it one day and I used it when I joined University"* [21 years old,male].

### Theme 3: Socialization reasons

Reasons related to socialization were the most frequently cited driving factor for PS. Peer pressure, curiosity, feelings of inferiority, local or cultural proverbs that justify PS use, poor socialization skills, and social celebrations were among the sub-themes emerged.

### Peer pressure

Peer pressure was reported among the students. Students experienced peer pressure from other students when encountering dormitory life, classmates, older or more ‘experienced’ students, groups and the networks they establish.

*Here [in University], you’re eager to build a friendship with other students. Unfortunately, you may come across students with exposure to substance use. […] Then, what comes is invitation, just to try it. We all are young and the peer influence is stronger* [23 years old, 5^th^ year].

Freshmen students often experienced peer pressure from older students on campus, and were influenced by socialization cues. Conversely, senior students purposefully approached freshmen students to benefit from sharing the younger students’ money in order to cover the cost of substances. Consequently, the older students facilitated freshmen initiation of substance use. One KIR stated:

*Fresh[men] students come with more money while they join University. Then, they build a friendship with senior students who smoke a cigarette, chew Khat or drink alcohol. Therefore, the freshmen start using substance following the pressure of the senior students* [KIR-2].

Freshmen students’ discomfort with refusing peer invitations to experiment with PS made them vulnerable to the use. A male user underlined this view:

*[…] actually, you may refuse once or twice; but if a user convinced you that it doesn’t have any consequence it will be difficult for you to resist more* [21-years, 3^rd^-year].

Participants also frequently mentioned that students consider using substances as an indication of worldliness or being more modern. Freshman students and those from the semi-urban residence are often motivated by this factor. One user’s FGD participant stated this:

*Smoking is mostly done because others are doing it. We consider that he is smoking because he is modernized (they used a local term “Arada”), so, someone wants to looked modernized and smoke* [22-years, User].

### Feelings of inferiority

Participants reported student variations in residence background, academic performance, pocket money they receive from family, dressing, and communication styles influential in making students feel inferior to their peer. In particular, students from rural or semi-urban residence feel inferior, which pushes them to PS use relieve associated anxiety linked to it. One PS user KIR reflected:

*We may get relaxed for the moment in the dorm following the substance we used. The non-users are considering it as an indication of joy and pleasure. They assume that we are from richer family while they are not. [KIR-2].*

Such feeling was also evident among those who used PS in lesser amounts, the less costly type and for shorter periods of time. The situation drove them to diminish the difference whenever they got an opportunity.

### Poor socialization skill

Participants also reported that students’ poor skills for creating and maintaining social relationship made them isolated from peers, thus linking with PS use. In addition, students who quarrel with dormitory mates or classmates, or those with recent breakups of friendship (including with girlfriend/boyfriends) preferred to spent most of their time alone and restrict their social interaction. According to the participants, this situation motivated students to consider PS use as means of getting relief.

A male user stated,:

*One year back, one student was involved in excessive drinking and cigarette smoking following break up with his girlfriend. Finally, he commits suicide* [27-years old, 3^rd^-year].

KIRs also shared their experiences that students often participated in extensive substance use following someone declining their request for friendship or after a breakup with opposite sex.

### Ceremonies & Festivities

Participants also disclosed that events like cultura holidays and birthday celebrations facilitated initiation of PS use, particularly alcohol drinking. A KIR underlined that the majority of students left off of campus for an event celebration, which constituted a breakdown event for most. They then wanted to repeat it again and some continued to use PS frequently. A female non-user stated her observation:

… *A female student who never drank alcohol may go to her friend’s birthday celebration. […] After drinking, she may get out of her control and involve an activity that she may not want to do in normal condition"* [2nd-year, female-student].

### Theme 4: Physical environment

Easy access to substance use, the absence of alternative entertainment activities and excess free time also emerged as drivers for PS use.

### Ease of access

Participants repeatedly mentioned that easy access to khat, alcohol, and cigarettes around the campus drives students to use it. The numbers of Khat Vendors, Shisha houses, and bars are increasing with time around the gates of the campuses. A female user in an FGD stated it this way:

*“… when you fail to handle the academic issues in the university, you would look out to the bars in front of the gates. No need to expense a lot, no need to be tired because it is just there in front of the gate*” [21 year-old,3^rd^ year].

Participants also stated that it was common to see students making a line at shops in front of the gate to buy cigarettes after lunch.

Participants also noted that easy access to substance contributes to relapse of use after quitting. A female student stated it like this:

*“Let say, you have decided to cut it down. Then, the problem would be where would you spend your time? You are already surrounded by the substances. You may decide to sleep without taking alcohol; however, you are hearing disturbing music from the bars for the whole night, perhaps it may be the music you like. Then, you [feel the] urge to go there*” [21-years-- female user].

Similarly, a female smoker stated,” Sometimes when there is no class, I visit my aunt for a week. I totally stop smoking then. But, when I come back again to the campus, I would start it again” [KIR-2].

### Shortage of alternative entertainments

Participants frequently mentioned the shortage of alternative entertainment activities drives students to PS use. The university compound has only a limited number of entertainment areas, which causes students to feel frustrated. This pushes the students to use PSs. A male user stated it:

*There are no entertainment centers in the compound. The students are closer to the substances than to other alternatives to spent time. Fresh students are often attracted to the substances because there are no other alternatives* [21-years-old,3^rd^-year].

### Excess free time

Along with the shortage of alternative entertainment centers, presence of excess free time was also reported to be linked with PS use. It was particularly reported during the first and second months of each academic semester because instructors do not start class on the expected start date, which creates an opportunity to have excess free time for PS use.

### Theme 5: Sub-optimal institutional support

Low administrative support was also reported to be linked with PS use. Participants mentioned three issues: the lack of alternative entertainment centers, poor enforcement of rules that restrict access to and use of PS around the campus, and low availability of interventions that offer help to quit PS use. Weakness in enforcement of the regulations related to substance use inside the university compound also resulted in continued use by students subsequently. A member of the student representative office also mentioned:

*Had it been followed by serious administrative measurements and I became aware of it, I would use it less in the compound"* [23-years old, 4^th^-Year].

Some participants also raised the issue of advertisement for alcohol beverages around campus as one that also drives students to consider using the substance. Posters, billboards, other media, and campaigns for concerts around the campus were all reported as contributors to intention towards and use of substances among students.

### Theme 6: Dissatisfaction with academic conditions

Participants reported dissatisfaction with academic conditions as frequently related to PS use. The participants explained this link in relation to low academic performance (perceived and actual), low quality of education provided, and hopelessness regarding future education and career.

### Perceived and actual academic failure

Fear of academic failure was reported as closely linked with students chewing khat, which becomes much more apparent when exams approach. Students assumed that it would help them to stay alert for reading. However, after their first use, they reported continuing to use it even after the exams were over. An FGD discussant reported,

*[…] the main reason why students do start substance use on this campus is during stress preceding an exam. If students do not cover the handout provided or if they know that they scored lower on the exam. They start smoking and khat chewing to relief the anxiety* [22-year-old,5^th^ year].

Participants also mentioned students start to use substance at the end of the academic year following low academic performance. An FGD discussant stated:

*Think! If the efforts you made over the year were not fruitful, it is really disappointing. Students fail to score pass mark in our department and many of them initiate substance use* [22-years, 2^nd^-year].

### Factors related to quality education

Participants mentioned that University instructors’ sub-optimal effort to educate the students is also implicated in PS use. Instructors were reported as reluctant to start and finish the course in a timely manner. Thus, some chapters of the course were often left uncovered and given to students as a reading assignment for the final exam. In addition, there were reportedly instructors who did not suggest reading materials, did not account for student’s variation in receptiveness to the class material, did not show scores for assessment, and were not transparent while grading. Participants reported that the psychological burden related to these issues was linked with PS use. A female FGD participant stated:

*It is common for instructors to provide us reading material today for an exam on the following day. He may also tell us to read ourselves for all the uncovered chapters. He is [beyond questioning] in grading. […] They are reluctant to show your assessment results* [21-years old, 3^rd^-year].

Additionally, the students mentioned that dissatisfaction with the limited educational inputs, including for example a shortage of books in a library and internet connection to download supporting materials, mediates low satisfaction, and is linked with PS use.

### Feeling hopelessness about future and career

Though students mostly reported initiating substance use for reasons like entertainment, a way to pass time, for studying, as well as others, one very relevant factor was hopelessness about their future life and career. A 3rd-year female substance user stated:

*Alcohol gives pleasure and entertainment while it is in a limited amount and occasionally. However, what makes you repeat it is hopelessness: hope regarding your future life and career* [21-years, 3^rd^-year-Female].

A KIR also stated:

*For many senior students, their future life looks empty. They have lost the thoughts they build during pre-university but [are] filled with none in the University. You may see that your previous wisdom and thought has collapsed in the new environment [university] but you didn’t get its replacement. This creates a vacuum and leads you to propose life is empty! Everything is empty! […] and students find substance use as the place to prove their proposition* [KIR-1].

Freshmen students may be dissatisfied with their assignments based on their self-evaluation related to a future career. The poor perception of possibility getting a job in their area of the study also creates anxiety for students, which in turn predisposes them to PS use.

An additional file also provides more quotes on each theme (See Additional file 1).

## Discussion

The study identified a range of individual, interpersonal and organizational drivers for PS use among university students. Initially, the students suffered from loneliness and feelings of helplessness related to lack of parental oversight. Loneliness, defined as a subjective deficiency of social relationships in a quantitative or qualitative way, was reportedly associated with indulging in PS use [7, 24]. The levels of moderate to severe loneliness experienced among university students were reported to be 35.6% in Germany [25], and 60% in Turkey [26]. Loneliness, which is likely to be more frequent among first year students, is also correlated with poor parental status, divorced families, and low levels of engagement in romantic relationships [26]. Conversely, parental supervision and living with family reduces risk of substance use [27–29]. Our results, and those of other studies thus suggest that absence of parents, and loneliness, may drive students to consider PS use.

Students’ previous exposure also emerged as being an associated factor for PS use in our study and in others. Lifetime experience of substance use before students join university and common use among the peer community may contribute to use in university [12, 30–33]. In addition, students who have lived in a place where PS use is common are more likely to use it in university [34, 35]. In addition, students with family/relatives who use PS such as alcohol, khat, and cigarettes [6, 7, 9, 20] are more likely to use these substances in university.

Peer pressure emerged as a prominent socialization-related factor linked with PS use. Students used PS to be accepted in their social circles. This finding is consistent with previous studies in Ethiopia and abroad [4, 7, 36, 37]. Psychoactive substance user students often perceived that their peers also used them, sometimes more frequently than themselves [7, 29]. The current study explored the powerful influence of local norms in creating peer pressure. The norms communicated the benefits of use, inciting new students to experiment.

In addition, students’ feeling of inferiority was another factor related to in relation to PS use among the students. Feelings of inferiority among young people were described to be associated with anxiety and depression, leading students to consider using PS for status reasons. Students entering universities could potentially vary in socio-economic background, academic performance, and intellectual ability, which could be related to feelings of inferiority. Similarly, previous international studies also show that university students’ feelings of inferiority (such as poor body self-image) could result in stress and procrastination [38–41]. In these situations, the students may consider PS use as coping up mechanisms for the inferiority.

To establish and maintain relationships is not an easy task for university students. Failure to establish or maintain relationships or friendships with the same or opposite sex could be followed risky behaviors like problematic PS use, as was reported in our study. Other studies identified the use of PS as a means to get relief from anxiety [8, 17, 29]. For example, Alcohol is perceived to make people more sociable, braver and stronger, relieve anxiety and reduce fears of conflict situations [17, 35]. Event celebrations including birthdays, holidays, post-exam parties, and others also was also found as strong socialization reason for PS use among university students in our studies. These are also acknowledged in previous studies [42–44].

Physical environments conducive to PS use around campuses similarly were reported as being linked with PS use in our study. With the limited range of recreational alternatives inside the university compound, students considered PS use to relax and pass time. Use of substances to relax is among the most commonly mentioned reasons for PS use among university students in other studies [7, 8, 29]. In addition, the perceived and actual ease of access to the PS created motivation to use it among participants of the current study. Access to PS use influences young people’s PS use [6, 35, 45, 46]. This may imply a need to impro theve range of recreational alternatives for students. Universities in lower income countries may apply less attention to provision of quality and quantity recreational options for the students, which should be remedied.

The current study also suggested that there is sub-optimal institutional support to restrict access to PS use around the campuses, and to assist students who may be suffering from substance abuse. The perceived ease to access PS in our study was high. Moreover, there was weak enforcement of organizational rules and regulations to restrict access. Media campaigns and promotion of substances around campuses was also reported. Previous studies have illustrated that media predisposes experimentation with PS use among university students [36, 45, 47].

Dissatisfaction with academic conditions was reported in relation to PS use in the current study, and was explained by dissatisfaction with: the assigned field of study, anxiety related to future employment opportunities, poor academic performance, and low quality of education provided. Academic dissatisfaction was also reported as reason for PS use in other Ethiopian settings [34, 48]. Students in our study and in others reported use of stimulants to help them concentrate [7, 15]. An important finding here was that hopelessness about career and future life in general was linked with consistent PS use. This was similarly reported in relation to cigarette smoking among college students in Turkey [49]. A previous study in Ethiopia also linked feelings of hopelessness with PS use among senior students [48]. Students’ principles, salient beliefs, and their approach to life may transform as they proceed through university with multi-dimensional exposure to the environment and campus life. Failure to successfully to adapt may result in hopelessness, and association with self medication using PS.

### Strengths and limitations of the study

While the study benefitted from strengths including local research team who had expertise in qualitative research and were aware of cultural and social context within the student and university environment, the study is not without limitations. The first limitation is the potential reliability of self-report by participants on their substance use (including those who may have substance use disorders). Self-report may be incorrect or incongruent, and individuals with substance use issues may lack insight into their own motivations. In addition, participants may have omitted information on the basis of social desirability, leading to some bias.

## Conclusions and recommendations

University students in Ethiopia, and particularly in Mekelle, reported experiencing multiple factors linked to psychoactive substance use. These factors, or drivers, were found at students’ individual and interpersonal level, and were also evident at structural and organizational levels. Loneliness and previous exposure were linked with PS use among university students. Students’ inability to overcome stressful issues such as social setbacks and university conditions may be an important factor related to substance use disorders. In addition, academic matters, sub-optimal physical and organizational environment, were reported to be linked with PS use among this population. The current study suggests an urgent need to provide proactive multilevel interventions (individual, interpersonal and organizational) to address a range of driving factors among university students to prevent morbidity and disability arising from substance use disorders. Further stronger studies such us grounded theory approach may be helpful to consolidate the drivers for PS use among students.

## Additional file

Additional file 1: Codes-quotations list. (DOCX 237 kb)
