# Supplementary material for: A qualitative study of drivers of psychoactive substance use among Mekelle University students, Northern Ethiopia
Source: Subst Abuse Treat Prev Policy. 2019 Mar 4;14:11. doi: 10.1186/s13011-018-0190-1 (PMC6398241; doi:10.1186/s13011-018-0190-1)
Supplement: Supplementary file 1 — Codes-quotations list. (DOCX 237 kb) [file 13011_2018_190_MOESM1_ESM.docx]

**Codes-quotations list**

**Code-Filter: Code Family "3_Motivating factors for substance use"**

______________________________________________________________________

HU: PASA atlas January 18-2018

File: [C:\Users\user\Desktop\PASSA quali_recent\PASA atlas January 18-2018.hpr7]

Edited by: Super

Date/Time: 2018-01-20 12:20:50

______________________________________________________________________

**Code: Motivators for substance use {1-0}**

**P 5: 13_Male KIR_BC_Round III.pdf - 5:11 [???? ??????? ?????? ???? ?????..] (3:269-3:736) (Super)**

Codes: [Motivators for substance use - Family: 3_Motivating factors for substance use]

No memos

የዯምብ ኣስከባሪዎች የተማሪዎች ፖሉሶች

የዱስፒሉን ኮሚቴዎች 4 ቱ የተማሪዎች ህብተር 2 በዱፓርትመንት የተማሪዎች ፖሉሶች ናቸው ኣንደ ዯሞ የተማሪዎች

ምክትሌ ፕሬዝዲንት እነሱ የነገሩኝ ነገር በቃ ከባሇፇው የነገርኩህ ነገር የተሇየ የሇውም ተማሪዎች የሚገፊፌዋቸው ነገሮች

ከግቢ ተፅኖዎች ኣለ ከቤተሰብ ኣሇ እንዯዛ ከቤተሰብ አይን ጥቅሙ ያሇው ነገር እዚይ ያለ ተማሪዎች ከቤተሰብ

የሚሊክሊቸው ብር ሉሆን ይችሊሌ ወይም ዯሞ ችግር ሃሰብ ሉበዛ ይችሊሌ እና ከዛ ነገር ሇመውጣትጓዯኛ ጋር እንትን ብል

ይሄዲለ ላልቹ ዯሞ በገቢ ውስጥ ዯሞ ብዙኣይነት አለ የትምህርት ሉሆን ይችሊሌ የጓዯኛ ገፉት ሉሆን ይችሊሌ እነዚህ

ካበሇፇው ከነገርኩህ የተሇየ የነገሩኝ የሇም ምክንያቱ ተማሳሰይ ናቸው እና እነዛ ነገሮች ናቸው እነሱ የሚገፊፌዋቸው ኣለ

______________________________________________________________________

**Code: Motivators for substance use- abscence of alternative entertainment {14-0}**

**P 1: 1_Female Users FGD in Busness college.docx - 1:10 [???? ??? ?? ???? ???»? ??? ???..] (39:39) (Super)**

Codes: [Motivators for substance use- abscence of alternative entertainment - Family: 3_Motivating factors for substance use]

No memos

ከሰዓት ወይም ማታ ኣከባቢ ቴሌቬ»ን እንኳ በኣንድ ብሎክ ምንም ነገር የለም።ስለዚህ ኣብዛኛዉ ሰዓታችነ ከሰዓት በባላ ያለዉ ወደ መዝናኛ ነዉ ራሳችን የምንወድዉ።

**P 1: 1_Female Users FGD in Busness college.docx - 1:31 [?? 1:-?? ????? ?? ?? ????? ???..] (62:62) (Super)**

Codes: [Motivators for substance use- abscence of alternative entertainment - Family: 3_Motivating factors for substance use]

No memos

ኮድ 1-እኔ ምክንያት ነዉ ብዬ የማስበዉ ተስፋ መቁረጥ ነዉ።ኣብዛኞቻችን ምንም ይሁን ምክንያታችን ለሂወት ትርጉም ማጣት።ኣሁን ትርጉም ኣጥተህ መጠጥ ብትጀምር እሱም ደግሞ ይበልጥ ትርጉም ሊያሳጣህ ይችላል።ፍህ ማለት ስትጀምር ደግሞ ሁሉን ነገር ቁስ ይሆናል።እና ኣንዱ ሱስ ራሱ የሱስ ሂወት ራሱ እንድትለምደዉ ያደርግሃል።ትርጉም ኣልባ ሂወት ማለት ነዉ።እቤት ስንሆን መሸሸግያ ኣለን ት/ቤት ቢደብርህ ቤትህ ሂደህ ትሸሸጋለህ እዚ ግን እንደዚህ ኣይደለም።ሙሉ ግዜ እዛዉ ነህ መሸሸግያ የለህም።ልትሸሸግበት የምትችልበት ይሄ ብቻ ነዉ።

**P 1: 1_Female Users FGD in Busness college.docx - 1:53 [?? ??? ????? ????? ??? ??? ???..] (83:83) (Super)**

Codes: [Motivators for substance use- abscence of alternative entertainment - Family: 3_Motivating factors for substance use]

No memos

ግቢ ዉስጥ የተመቻቸ ኣይደለም ያለዉ ላይፍ ሃርድ ነዉ የሚያደርግብን።እኔ እንጃ በላይፍ ታይማችን ዉስጥ እንደ ግቢ የተቀጣንበት ኣንዳንዴ ለመማር ነዉ ወይስ ለመቀጣት ነዉ የመጣሁት የምልበት ጊዜ ኣለ። ላይፋችን የሚያበላሸዉ የሁላችንም ግቢ ያለ ነገር ነዉ።ሃይስኩል ጥሩ ግሬድ ይዘህ መጥተህ እዚህ ላይፍ 2 ማምጣት የሚያቅተን ጊዜ ኣለ።ማን ሱሰኛ መሆን ይፈልጋል ማን ሂወቱን ማበላሸት ይፈልጋል።

**P 2: 10_M_IDI_Male non user BC.docx - 2:25 [?????? ???? ???? ????? ???? Mi..] (35:35) (Super)**

Codes: [Motivators for substance use- abscence of alternative entertainment - Family: 3_Motivating factors for substance use]

No memos

እነዲያውነ መዓልቲ መዓልቲ ከይሰከሩ ብያንስ Mimimum 10 ከይሰከሩ ገለ ከይሰከሩ አይአትውን Mimimum ምሸት ምሸት ክፍጠር ዝኽእል፡፡ ምናልባት ሎምዘበን እካ ቀኒሱ እዩ ምስቲ አብ ግቢ ምስቲ DSTV ግዛዕ ምዛዕ ስለ ብመጠኑ ተማሊእሎም ምውፃእ ተከልኪሎም ደሓን ኮይኑ አሎ

**P 2: 10_M_IDI_Male non user BC.docx - 2:58 [?? ???? ?? ???? ??? ???? ???? ..] (69:69) (Super)**

Codes: [Motivators for substance use- abscence of alternative entertainment - Family: 3_Motivating factors for substance use]

No memos

ብኡ ተወሳኪ ከዓ ሕፅረት ከምቲ ዝበልኻ ሕፅረት ናይ ምዝንናይ በተለይ ናይ መቐለ ዩኒቨርስቲ ኢሉ እውን አብ ትግራይ ዩኒቨርስትታት ዝረአ ችግር መዝናነይ የለን ሕፅረት አሎ ብጣዕሚ፡፡ ሐዚ እንታይ እዩ ዩኒቨርስቲ ኮይኑ ወይ DSTV ኩዕሶ ትሪኤሉ ተዘይ ሀልይዎ ወይ አጋላት ቃና ክሪአ እንተደልየን እንተዘይሃዩ

**P 2: 10_M_IDI_Male non user BC.docx - 2:59 [?? ?? ???? ?? ???? ?????? ??? ..] (72:72) (Super)**

Codes: [Motivators for substance use- abscence of alternative entertainment - Family: 3_Motivating factors for substance use]

No memos

ሐዚ እዩ ዝማላእ ዘሎ እምበር አይነበረን ሓንቲ DSTV ነይራ ዓሚ ሓንቲ አዳራሸ ሕሰቦ ሓንቲ አዳራሸ ንዝኩሉ ተምሃራይ ሓንቲ ን500 ከም ግዛዕ አቢላ 300 እንተ ሒዛ ብዙሕ እዮ ሕሰቦ ዝኢ ኩሉ ብዙሕ ሸሕ ተምሃራይ አጋላት አይነበረንን ሪኢካ ከምኡ እናገበረ ተሸጊረን ሐዚ ይቅረፍ አሎ እዩ፡፡ ይአትወለን አሎ ግን ከምኡ ክዝናነዩ መዝናነይ ቦታ ንሱ ጥረሕ አይኮነን ኩዕሶ ግዛዕ አይኮነን ናይ ሰብ ድሌት ብዙሕ እዩ፡፡ ዝኾነ መናፈሻ ነገር ከድሊ እዩ ምሽይ ክብል እንተሎ ዝፃወትሉ ፡፡ ዘይብላ እነተኾይና ባዶ ሜዳ ገለ እነተኽይኑስ ከፍ ኢለም መቸም አብ እምኒ ክጫወቱአይኽእሉን፡፡ ዝኾነ ክህሉ ዝኾነ Green meatt ግዘዕ ምዛዕታት ዝኮነ መዝናነዩ ከምኡታት ስለ ዘየለ ክዝናነዩ አይኽእሉን፡፡

**P 2: 10_M_IDI_Male non user BC.docx - 2:60 [?????? ?????? ?? ?? ???? ??? ?..] (72:72) (Super)**

Codes: [Motivators for substance use- abscence of alternative entertainment - Family: 3_Motivating factors for substance use]

No memos

እነዲያውመ መብዛህትኡ ግዜ ንሱ እዩ፡፡ ብሓቂ እዚ ግቢ ናይ ቴንሸን (Tension) በታ ጥራሕ ይመስሎም እሞ ክላ ካብቲ ግቢ እዙይ ንውፃእ ኢሎም እቲ ግቢ ባዕሉ ውነ ከምቲ ናይ ጭንቂ ገይሮም ይወስድዎ ካብኡ ከተማ ከይዶም ሰቲዮም ይመፁ ወይ ግዛዕ ይገብሩ አብ ትሪፊ ሰዓቶም ከፍ ዝብሉላ ዝናፈሱላ ቦታ እንተዝህሉ ነይራ እንዲያውም ናብ ደገ ዝወፅኡ ሰዓት አይምሃለወን እቲ ሰዓት አብዙይ ምተቀተለ ነይሩ፡፡

**P 9: 17_Male User_Adihaki campus.docx - 9:13 [?? ????? ???? ????? ?? ?? ????..] (27:27) (Super)**

Codes: [Motivators for substance use- abscence of alternative entertainment - Family: 3_Motivating factors for substance use]

No memos

ምቹ አይደለም መዝነኛ ስለሌለው ወዳ ሱስ ያመራል በዚህ ምክንያት ወ

**P 9: 17_Male User_Adihaki campus.docx - 9:18 [????????? ???? ??? ?? ???? ???..] (42:42) (Super)**

Codes: [Motivators for substance use- abscence of alternative entertainment - Family: 3_Motivating factors for substance use] [Motivators for substance use- Availability of the substances - Family: 3_Motivating factors for substance use]

No memos

ኢንቨይሮንመንቱ አከበቢ ውስጥ ብዙ ነገሮች መዝነኛ የሉም በላይኛውም በር በሰው በደርፉር በር መጠጥ ቤቶች እና ጫት ቤቶች

**P13: 20_KIR 1_ 3rd round interveiew_Business Compus_mekelle Univeristy .doc - 13:28 [?? ??? ??? ????? ?? ?? ???? ??..] (33:33) (Super)**

Codes: [Ceasing: Challenges to cease substance use - Family: 8_Intention to cease and experiance of relapse for substance use] [Motivators for substance use- abscence of alternative entertainment - Family: 3_Motivating factors for substance use]

No memos

*ከዛ ደግሞ ከሱስ ወጥተንስ ሌላ ምን አለ፡፡ ምን እናደርጋለን ያው ደብርት ውስጥ ነው የምንወድቀው የሚል ፍራቻ ደግሞ አላቸው፡፡*

**P16: 23_KIR 2_1st interveiw_Busness campus Mekelle Univeristy.doc - 16:8 [?? ?????? ?? ?? ?? ?????? ??? ..] (124:124) (Super)**

Codes: [Motivators for substance use- abscence of alternative entertainment - Family: 3_Motivating factors for substance use]

No memos

እዛ እምጨምረው ሰው ወዳ ሱስ የሚገባበት ሁኔታ የዚች ኣገር የራስ የድህነት ነው የትም መሄድ የለውም ደህና ከሆንክ ደህና ኢኮነሚህ የዳገ ከሆነ ከትምህርት ነፃስትሆን የሆነስራ ትስራለህ ኢዚህ ጋ ግን ምንም የምትከራው የለም ከት/ት ነፃ ሰትነህ ከነዚህ ምንም መዋይ የለህም ስለዚህ የምትገባበት መጃመሪያ ድህነት ራሱ ነው ምንም ስራ የለም ለረሱ ለራሱ የምትዝናናበት ቦታ የለም ስለዝህ ሌለ ቦታ መሄድ ከሌለህ ጋደኞችህ ወዳዛ ሊሄዱ ታያለህ ኣንተም ተከትለህ ትገባለህ ስለዚህ መሰረቱ ማንኛዉም ሰው ወዳ ሱስ የሚገባበት ምክንያት ራሱ የዚች ሀገር ድህነት ነው

**P16: 23_KIR 2_1st interveiw_Busness campus Mekelle Univeristy.doc - 16:10 [??? ??? ??? ???? ?? ?? ???? ??..] (124:124) (Super)**

Codes: [Consquences of substance use: Pshychological distress - Family: 7_Experiance of consquences of substance use] [Motivators for substance use- abscence of alternative entertainment - Family: 3_Motivating factors for substance use]

No memos

ኣንተ አይን ይሄከ እንዲህ ነው ብሎ ኣንተን ሊመልስ እንደመሞከር ቤተስከም ማንም ዝም ብሎ የጨቀጭቀሃል እዛው ውስጥ እይለህ ኢሄየት የደርስል ከዛ ኣይልፍም ሁሉ እንደዘ ናቸው ዝም ብለው ነው የሚጨቃጨቁት ስለዚህ ምንም ኣየመጡም ተጨቃጭቀው የተውኛል ብሎ ተመልስ ይገባል በጠቃለይ ግን ምክንያቱ ድግነት ነው

**P20: 5_IDI_Male user-BC.docx - 20:16 [??? ??? ?? ????? ????? ????? ?..] (38:38) (Super)**

Codes: [Motivators for substance use- abscence of alternative entertainment - Family: 3_Motivating factors for substance use]

No memos

በግቢ ውስጥ ምቹ አይደለም በጠቃለይ ለተማሪው ብዙ መዝናኛዎች የሉትም ተማሪው ለሱስ ይቀርባል ከሌላ ነገር ይልቅ ማለት ነው መዝናኛ ቦታዎች የሉት ፍሬሽ ላይ አብዛኞቹ ልጆች ወደዚህ ነገር ነው ቀድመው ለመግባት የሚፈልጉት ከሱስ ውጪ ሌላ ብዙም ነገር የለም አከባቢው ላይ ግቢ ውስጥም ያሉ መዝናኛ ቦታዎች ትንሽ ናቸው መቀመጨዎች ቦታዎች የሉትም እናም አብዛኛውን ሰው በቃ ውጪ ወጥቶ ጫት ቤት ቁጭ ማለት ነው የሚፈልገው በዛ ሰአት ላይ ልምድ ይሆንበታል፡፡

**P20: 5_IDI_Male user-BC.docx - 20:24 [??????? ???????? ?? ????? ????..] (52:52) (Super)**

Codes: [Motivators for substance use- abscence of alternative entertainment - Family: 3_Motivating factors for substance use]

No memos

እንደልካችሁ ኢንቫይሮመንቱ ምቹ አይደለም እዚጋር አሁን ብዙ ልጆች በሱስ ምክንያት ከዚህ ግቢ ተባርረው ብዙ ነገር አቅድመው እንካን የሚመጡ ተማሪዎች አሉ ማለት አገራቸው ሄደው ማለት ነው ግን እዚህ ሲመጡ በቃ ከአንድ አንድ ምንም የሚያውቅት ነገር የለም አዲስ ነገርም ለመልመድ ከባድ ነው ኢንቫሮሜንቱ ሌላ ቦታ ቢሄድም ብዙ ነገር የሚደረግበት ቦታ የለም በላይ በርም መጠጥ ቤት ነው እዚህም (ዳርፉር) ዳግም መጠጥ ቤትና ጫት ቤት ነው እና ወደዚህ ነው የሚመለሱት ድጋሜ ማለት ነው ብዙ እንካን ተቀጥተው በዛ ነገር ድጋሜ ነው የሚመለሱት ምንም አማራጭችመ ብዙም ስለሌሉ ማለት ነው

______________________________________________________________________

**Code: Motivators for substance use- Abscence of family support in campus {3-0}**

**P18: 3_M_FGD_Male User's _Ayder.docx - 18:18 [?? ?? ????? ???? ??? ???? ? ??..] (47:47) (Super)**

Codes: [Motivators for substance use- Abscence of family support in campus - Family: 3_Motivating factors for substance use]

No memos

ሓደ ካብ ቤተሰብኻ ምፍላይ ማለት ንባዕሉ ከ በቃ ኸቢድ እዩ ከም ድሌትካ ኢካ ትከውን አብ ገዛኻ ተኾይንኻ ሪኡኒ አኮይ ሓፍተይ ድያ ገለ እናበልካ ትስቀቅ ኢኻ ብሙሉኡ አይትውድእን አብ ዘይዓድኻ ግን ዘይዓድኻ አይዓድኻን

**P19: 4_IDI_ Male user BC.docx - 19:28 [?? ??? ??????? ????? ??? ?????..] (81:81) (Super)**

Codes: [Motivators for substance use- Abscence of family support in campus - Family: 3_Motivating factors for substance use]

No memos

አዎ አሁን የዩኒቨርስቲ ተማሪዎች በሱስ እንዲጠመዱ ምክንያት የሆንበት አንዱ ከወላጅ መራቅ ነው ብዮ ነው የማምነው እኔ Because ተቆጪ የለህም፣ ተሳዳቢ የለህም፣ ገልማጭ የለህም ዩኒቨርሲቲ የሚቆጣጠር ግቢ ውስጥ የሆነ activity እንዳተፈፅም ነው፡፡ ውስጥ ላይ እንዳታጨሰ፣ ውስጥ ላይ እንዳትቅም፣ ውስጥ ላይ ፓፋ እንዳታደረግ ትችላለህ፡፡ ስለዚህ ተቆጫጫሪ የለህም፡፡ ከሃገር ተሰደህ መጥተህ እበህጋ ማንም ባልማኝ የለኝም ብለህ ነው የምታሰበው እና ያ ይመስለኛል የሚገፋፋቸው፡

**P19: 4_IDI_ Male user BC.docx - 19:30 [?? ?? By the way ???? ?? ??? ?..] (87:87) (Super)**

Codes: [Motivators for substance use- Abscence of family support in campus - Family: 3_Motivating factors for substance use]

No memos

ያው እኔ By the way ከግቢው ውጪ ይሁን ግቢ ውስጥ Normally ማስብ ያልብን የራሱ ኣስትሳስብ ይመስለኛል በawareness ይመስለኛል ዕድገቱ ይመስለኛል ይህ influence ግቢ ውስጥምከ ግቢ ውጭም influence እኮ የማይበግራችው ኣሉ ብጥሩ ተቀርፆ ያደገ ልጅ ማለት ነው እና የራስህ ኣስትሳስብ ነው already ዋናው ይቤተስብ ጉዳይ ሲሆን ሁለተኛ ድግሞ ከቤተስብ በተጨማሪ ኣንተ ራስህ ኣለህ ስለዚህ የራስህ የኣስተሳስብ ጉዳይ ይመስለኛ

______________________________________________________________________

**Code: Motivators for substance use- Acadamic related {27-0}**

**P 1: 1_Female Users FGD in Busness college.docx - 1:27 [?? 3:- peer pressure ?????? ??..] (58:58) (Super)**

Codes: [Motivators for substance use- Acadamic related - Family: 3_Motivating factors for substance use] [Motivators for substance use- Educational system it slef - Family: 3_Motivating factors for substance use] [Motivators for substance use- Peer pressure - Family: 3_Motivating factors for substance use]

No memos

ኮድ 3- peer pressure እንዳላቸዉ ሊሆን ይችላል።ኑሮ ሲከብዳቸዉ ይመስለኛል ተማሪዉ ወይም ት/ት ሊከብደዉ ይችላል ወይም ላይፍ ግቢ ላይ ያለዉ ላይፍ ለምሳሌ ኣንድ ሴት ደምኛ ከተደረገች ተናዳ የምትጀምረዉ ሊሆን ይችላል እንጂ ደስ ብሎት የሚጀምር ሰዉ ይኖራል ብዬ ኣላስብም።

**P 3: 11_M_IDI_Male nonuser BC.docx - 3:28 [?????? ???? ????? ????? ????? ..] (40:40) (Super)**

Codes: [Motivators for substance use- Acadamic related - Family: 3_Motivating factors for substance use]

No memos

ከትምህርቱ አንፃር ትምህርቱ ተማሪውን ሲከብደው ወደ ሱስ ይገባል ፡፡

**P 3: 11_M_IDI_Male nonuser BC.docx - 3:30 [??? ??? ???? ????? ?? ?????? ?..] (41:41) (Super)**

Codes: [Motivators for substance use- Acadamic related - Family: 3_Motivating factors for substance use]

No memos

በግቢ ውስጥ ሳይሆን በፋሚሊም አለ በትምህርቱ አንፃርም ትምህርቱ በጣም የሚከብደው ከሆነ በቃ ራሱን ያለማጨናነቅ በዛበዛ ምክንያቶች ሱሱን እንትን ይላሉ

**P 4: 12_MAle KIR_BC_round .docx - 4:15 [??? ????? ??? ?? ????? ????? ?..] (13:13) (Super)**

Codes: [Motivators for substance use- Acadamic related - Family: 3_Motivating factors for substance use]

No memos

ከዚህ በተጨማሪ ኣዲስ ገቢ ተማሪዎች ኣንዳንዴ ከሚድርስባቸው የትምህርት ክብደት ለምሳሌ የህግ ትምህርት ክፍል ተማሪዎች በተለይ ማለት ነው እና የART ወይም የጥበባት ክፍል ተማሪዎች ለነዚ ሱሶች ተጋላጭ ናችው

**P 4: 12_MAle KIR_BC_round .docx - 4:16 [??? ??? ???? ????? ????? ??? ?..] (14:14) (Super)**

Codes: [Motivators for substance use- Acadamic related - Family: 3_Motivating factors for substance use]

No memos

ሌሎች ድግሞ ፍሬሾች እንደገቡ ተማሪዎች ማለት ነው ኣዲስ ተማሪዎች የሚፈልጉት ዲፓርትመንት ኣይነት ካለ ማግኘት እና ክፍል ሌላ ኣማራጭ ከማጣት ምክንያት ኣላስፈላጊ ሱሶች እንድሚደረጉ ነግሮኛል እሱ ማለት ነው።

**P 4: 12_MAle KIR_BC_round .docx - 4:34 [??? ?? ?? ?? ?? ??? ?? ?? ????..] (44:44) (Super)**

Codes: [Motivators for substance use- Acadamic related - Family: 3_Motivating factors for substance use]

No memos

አሁን እሱ ላይ እዚ ግቢ ማለት ነው እዛ ያልጀመረ ተማሪ More እዚ የሚጀምርበት ምክንያት አንድኛው ነገር ትምህርት ከብዶት ነው በቃ አሁን እዚ ያየሁዋቸው ብዙ የህግ ተማሪዎች አሉ ምንም እንዲመጡ በቃ አካለቸው ፊታቸው ስታየው እብጥብጥ ያሉ ናቸው ምንም ነገር የለባቸውም የምታየው ነገር ማለት ነው፡፡ እዛም ብዙም ብዙም አይጠቀሙም የነበሩ ማለት ነው፡፡

**P 4: 12_MAle KIR_BC_round .docx - 4:35 [?? ??? ????? ?? ??? ????? ????..] (45:45) (Super)**

Codes: [Motivators for substance use- Acadamic related - Family: 3_Motivating factors for substance use]

No memos

እዚ ሲመጡ ትምህርት በቃ በጣም የሚከብድ ስለሆነ እንትን ይላሉ በቃ ያነሱ አዕምሮ ዋቸው Active እንዲሆንላቸው እፆችን መጠቀም ይጀምራሉ ጫት መቃም ጫት ቤት አሁን ብዙ ግዜ የምታያቸው ተማሪዎች እነሱ ናቸው ያህግ ተማሪዎች ማለት ነው፡፡ የህግ ተማሪዎች ይበዛሉ፡፡እንዳውም የሚፃፍ ነገር ካለ እዛ ሆነው ነው የሚፅፋት እዛ ጫት ቤት ውስጥ ሆነው ማለት ነው

**P 4: 12_MAle KIR_BC_round .docx - 4:36 [???? ????? ??????? ????? ?????..] (45:45) (Super)**

Codes: [Motivators for substance use- Acadamic related - Family: 3_Motivating factors for substance use]

No memos

ብዙዎች ብዙዎቹን ያናገርካቸው ተማሪዎች ለመዝናናት ወይ ከድብርታቸው ለመላቀቅ ካልሆነ አልኮል አይጠቀሙትም ጫትን ግን በጣም More ይጠቀሙታል ስለዚህ ትምህርት በሚከብድበት ሰዓት ክብደት አሁን የደረሳቸው ዲፓርትመንት አርቶች አሁን በጣም ብዙ ፁሁፍ ይፅፋሉ እንደነገርኩህ ይፅፋሉ ደሞ ክላሳቸውም አብዛኛውን ግዜ ሌሊት ነው፡፡ የነሱ ማለት ነው ትያትር More Practically ስሚሰሩ ማለት ነው እና ያሀንን ነገር ለመወጣት ግድ እንትን ጫት ይጠቀማሉ More ማለት ነው ከዲፓርትትመንት ያነጋገረኩዋቸው እንደዚህ ነግረውኛል፡፡

**P 4: 12_MAle KIR_BC_round .docx - 4:37 [Other Social science ??? ?????..] (48:48) (Super)**

Codes: [Motivators for substance use- Acadamic related - Family: 3_Motivating factors for substance use]

No memos

Other Social science ሞልቶ በውጤትም ባይሆን መጀመርያ Sociology ሞልቶ ከሆነ አማርኛ የደረሰው ወይም እንግሊዘኛ የደረሰው ተማሪ ወደ ቤተሰብ እንዳይመለስ ቤተሰብ ስለዚህ ስለ ዲፓርትመንት የሚያውቀው ነገር የለም ደሞም ካብዙ ቦታም ተማሪዎች አሉ ከደቡብ ክልል መጥተው እዚ ትግራይ የደረሰው ተማሪ ነበር አወቃለው፡፡ ብዙ ተማሪ ማለት ነው፡፡ ሱማሌዎች ጋምቤላዎች እዚ ደርሳቸው ነበረ እና አሁን ጋምቤላ ክልል እንግሊዘኛ የደረሰው ተማሪ ተማሪ አይባልም በነሱ ባህል ማለት ነው

**P 4: 12_MAle KIR_BC_round .docx - 4:38 [??? ?? ????????? ?? ????? ????..] (49:49) (Super)**

Codes: [Motivators for substance use- Acadamic related - Family: 3_Motivating factors for substance use]

No memos

አሁን እኔ ያነጋገርካቸውም አሉ አንደንድ ተማሪዎች ማለት ነው እና ያለፍላጎት ስለደረሳቸው በቃ አንደኛ ተመርቂ ስራ አላገኝም የሚል Pressure አለ እነሱ ላይ ማለት ነው ያ ስላላ በቃ More of የሆነ እራሳቸውን የሚደብቁበት ነገር ነው የሚፈልጉት ማለት ነው፡፡ ስለዚህ በቃ በዚያ ምክንያት አንደሄዱ እንደሆነ አይቻለው ሁሉም የሚያደርጉት

**P 4: 12_MAle KIR_BC_round .docx - 4:48 [??? ??? ???? ?????? ???? ??? ?..] (58:58) (Super)**

Codes: [Motivators for substance use- Acadamic related - Family: 3_Motivating factors for substance use]

No memos

ሌላው ቅድም ያልኩህ የትምህርት ክብደት በረሱ ያረሱ ተፅእኖ አለው የሚገፋፉ ነገር አለው በከበደክ ሰዓት ሌላ ተማሪ የጀመረው ተማሪ በቃ ዶርሜ ውስጥ በቃ ጫት ሳይቅም የማያነብ ተማሪ አለ አሁን እኛ ጋ ማለት ነው፡፡ እና ሎሎች ጎዋደኞች ደሞ አሉት ደሞ እንደዚሁ ያነባሉ ፡፡ ግን እያደረ Effort እየጨረሱ በሚመጡበት ስዓት ጫት ቅሜ በቃ እስቲ ልመክረው በሚል እነሱ ጋ ይሄዳሉ እንደዚህ ይለምዱታል ማለት ነው

**P 6: 14_X_FGD male non-user at Ayder docx.docx - 6:4 [?? ?? ?????? ???? ???? ?? ?? ?..] (15:15) (Super)**

Codes: [Motivators for substance use- Acadamic related - Family: 3_Motivating factors for substance use]

No memos

ከዛ ደሞ በትምህርት ኣንጻር ኣይደር ግቢ ያው የጤና ሳይንስ ትምህርት ነው ትንሽ ጫና ኣለ ትምህርቱ ከዛ ኣንፃር ያው ትምህርቱም ትሽ ስለሚያጨናንቃቸው ወደዛ የመሄድ ልምድ በጣም እየሰፋ

**P 6: 14_X_FGD male non-user at Ayder docx.docx - 6:5 [?? 5 ??? ?? ????? ????? ?? ???..] (17:17) (Super)**

Codes: [Motivators for substance use- Acadamic related - Family: 3_Motivating factors for substance use] [SU_ Trend in tearms of time - Family: 1_Commonly used substances among univeristy students]

No memos

ኮድ 5 ማለት ሱስ እንዳለው እየጨመረ ነው በኣሁኑ ጉዳይ ማለት ኣይታሃቸው ማይገመቱ ሰዎች እያጨሱ እየቃሙ ምታገኛቸው ምን እንደሆነ ምክንያቱ ኣጋጣሚ በተላይ በተለይ ግን የመዲሲን ተማሪ በቃ ኣብዛኛው ያሉትን ኣብዛኛው የሚጠቀሙት በዚው በዛም ብትሄድ የመዲሲን ተማሪ ነው የሚጠቀመው ለምንደ ነው ምናምን ብትለው post ነኝ ጥንቀት ነበረብኝ ይልሃል እንደዛ እያሉ እየገቡ ነው ወደ ሱስ

**P 6: 14_X_FGD male non-user at Ayder docx.docx - 6:6 [post ??? ????? ??? ??? ?????? ..] (18:18) (Super)**

Codes: [Assumed purpose to strat substance use- Concentration - Family: 4_Percieved importance of subatance use by users] [Motivators for substance use- Acadamic related - Family: 3_Motivating factors for substance use]

No memos

post ማለት ተፈትነው ሲወጡ ወይም ከላይብረሪ ወጥተው ብቻቸው ሲሆኑ ወደ ሲጋራ መጨስ መግባት እምቢ ሲላቸው በጣት ይሞክራሉ ጫት በመጠቀምህ እንደዚህ እንደዚህ ትምህርት እንድታጠና ያደርጋል ምናምን ስትባል በዚህ ምክንያት ወይ መግባት እምቢ ሲላቸው ወይም መቀመጥ ሳይችሉ ወይም የትምህርት ውጤት ዝቅ ሲል ወደ ሱጉዳይ ይገባሉ እና በሱ ምክንያት ነው

**P 6: 14_X_FGD male non-user at Ayder docx.docx - 6:12 [?????? ??? ?? ?? ????? ??? ???..] (20:20) (Super)**

Codes: [Motivators for substance use- Acadamic related - Family: 3_Motivating factors for substance use]

No memos

ከማውቃቸው ቤዘድ ሁኜ ነው ምናገረው ፈተና ኣልፎ grade ሲወጣ ዝቅ ሲል ብዙ ዝቅ ሲል ብዙ ኣሁን ደግሞ ፈተና ኣልፎ gradeዱ ሲወጣ ደግሞ የትምህርት gradeዱ ትንሽ ዝቅ ሲል ብዙተማሪ እንደገና ወደዛ የማምራት ሂኔታ ኣለ በመዲንዲሱን others ኣሉ ብዙ ተማሪዎች ኣውቃለሁ ግን በmedicineን ይበዛል

**P 6: 14_X_FGD male non-user at Ayder docx.docx - 6:16 [???? ?? ?? exam ??? ???? page ..] (23:23) (Super)**

Codes: [Motivators for substance use- Acadamic related - Family: 3_Motivating factors for substance use]

No memos

ሁለተኛ ደሞ ስለ exam ፈተና በተለይ page ከበዛብህ ጫት ምናምን ተጠቀማለህ ጭንቅላትህ activate ያደርገዋል ምናምን ለግዝያዊ ስለሚባል እንደዛ ሚጠቀሙ ኣሉ በተለይ medicine ስለሚበዛባቸው ለእንደዛ ምክንያት ምናምን ብለው ይጠቀማሉ

**P 6: 14_X_FGD male non-user at Ayder docx.docx - 6:57 [??? ?? ??? ????? ???? ???? ???..] (81:81) (Super)**

Codes: [Assumed purpose to strat substance use- Relief from anxity - Family: 4_Percieved importance of subatance use by users] [Motivators for substance use- Acadamic related - Family: 3_Motivating factors for substance use]

No memos

እዚህ ግቢ በብዙ ምክንያት ሊጀመር ይችላል በእኔ ኣስተያየት ኮመን የሆኑት ሶስት ነጥቦች ኣሉ ኣንዱ ከሁሉም እንደተባለው የ መዲሲን ተማሪ ነው የመጀመርያ የሚያጨሰው እዚህ ግቢ እና እነሱ ለምሳሌ ኣስበሀዋል በ stress ግዜ ምናምን ግዜ ይጀምራሉ ማለት ፈተና ሲያልፍ ወይም ደሞ ፈተናው የሚነበብ ሃንድ ኣውት ሲበዛ ስትረሱ ለማቃለል ያው እንደተባለው ሲጋራ እና ጫት ምናምን ይጀመራል

**P 6: 14_X_FGD male non-user at Ayder docx.docx - 6:58 [??? ?? ??? ???? ?? ??? ???? ??..] (81:81) (Super)**

Codes: [Motivators for substance use- Acadamic related - Family: 3_Motivating factors for substance use]

No memos

ዋናው ግን ኣመቱ ሲያለቅ ነው ኣመቱ ሲያልቅ ብዙ ተማሪ ፖስት ሲበዛ ሲጋራ ዋናው ኣመቱ ሲያልቅ ያው ገሬዱ የሚሰራው ኣንዋሊ ነወ ብዙ ተማሪ ፌይል እና የዛኔ ሲያፈደርግ ኣስበው 12 ወር ተምረህ እንደገና ኣስራ ሁለት ወር ትደግማለህ ስትባልየሚመጣው ብስጭት አስብሀዋል እና ብዙ ተማሪ ከዛ ነው የሚጀምረው እኔ ከማውቀው ካለኝ እንትን 50 እስከ 60 percent ተማሪ ፋል ያደርጋል በየኣመቱ የተለመደ ነው ከነዚህ almost 90 percent ቱ ከወንዶቹ ይጀመራሉ ማለት ይቻላል እና የ gradeዱ ዚቅ ሲል ኣንዱ ምክንያት ነው

**P 6: 14_X_FGD male non-user at Ayder docx.docx - 6:59 [??? ?? ????? ????? ??? ??? ?? ..] (81:81) (Super)**

Codes: [Motivators for substance use- Acadamic related - Family: 3_Motivating factors for substance use]

No memos

ሌላው ደሞ ቀስበቀስ መበመሃል ደህና ማለት ብዙ ተማሪ ኣይደለም ሲያነብ ስትረስ ሲበዛው ስትረሱ ለማቃለል ኣንድ ኣንዴ ተማሬ ለምሳሌ ብዙም ኣይደለም ኣንድ ኣንድ ተማሪ ሁሉም ኣይደሉም ዋናው ግን ከግሬድ የተያያዘ ነው

**P 6: 14_X_FGD male non-user at Ayder docx.docx - 6:62 [ver nignt club ?? most common ..] (82:82) (Super)**

Codes: [Assumed purpose to strat substance use- Entertainment - Family: 4_Percieved importance of subatance use by users] [Motivators for substance use- Acadamic related - Family: 3_Motivating factors for substance use] [Motivators for substance use- Peer pressure - Family: 3_Motivating factors for substance use]

No memos

ver nignt club አለ most common ነው ሌላው እንዳልኩት የፈተና ስትረስም አለ ከgradu የተያያዘ ኣለኣይደል እና peer influence ኣለ እዚህ 3 ነገር የዚህ ግቢ ተማሪን affect የሚያደርጉ ነገሮች ና ማቸው

**P 6: 14_X_FGD male non-user at Ayder docx.docx - 6:64 [??? ?? ??? ????? ???? ???? ???..] (85:85) (Super)**

Codes: [Motivators for substance use- Acadamic related - Family: 3_Motivating factors for substance use]

No memos

ሌላው ደሞ ኣንድ ግሬሪንግ ሲስተሙ በተለይ የመዲሲን በኣንዴ የሚያነብቡት 300 እንስከ 400 ፔጅ ከ10 ነው የሚያዘው እንደዛ እያሉ ሚያነቡት ነገር በጣም ችግር ኣለ ግሬዲንግ ሲስተሙ ኣሁን የኛ ተሻሽለዋል ግን የተበላሸ ኣለ የመዲሲን ገና ኣልተስተካከለም አሁን ለምሳሌ እዚህ ያለ የራሱ ዩኒክ ነው ማለት በኢትዮጵያ ወደ 35 ሌሎች ዩንቨርስቲዎች ኣሉ ለብቻው የሚሰራው ግን ይሄ ነው በቃ ምክንያቱ ለምሳሎ ከ60 በታች ለምሳሌ 50እስከ 59 ብታመጣ ትወድቃለህ ስለዚህ በመዲሲን ዲ ካመጣህ በሌላውይቅር ትወድቃለህ

**P 6: 14_X_FGD male non-user at Ayder docx.docx - 6:65 [????? ????? ??????? ????? ????..] (89:89) (Super)**

Codes: [Motivators for substance use- Acadamic related - Family: 3_Motivating factors for substance use]

No memos

ኣንደኛው ምክንያት እንደተባለው በጭንቀት ምክንያት ብዙ ወር ወይም ኣመት ከምናምን የጣርክበትን እሱ ባዶ ሁኖ ስታገኝ በቃ ወደ ሞጥፎ ነገር ትገባለህ በጭንቀት ምክንያት ስለቤተሰብ ታስባለህ በዛ ኣመት ያወጣሀው ወጪ ብዙ ነገር ኣስበህ በቃ ሁሉም የራሱ ጉዳይ ብለህ ወደዛ ሱስ ትገባለህ ትገፋፋለህ ማለት ነው

**P 6: 14_X_FGD male non-user at Ayder docx.docx - 6:76 [??? ???? ?????? ???? ??? ?????..] (112:112) (Super)**

Codes: [Motivators for substance use- Acadamic related - Family: 3_Motivating factors for substance use]

No memos

ኣሁን ለምሳሌ የግሬዲንግ ሲስተም ስንል በኢትዮጵያ ዩንቨርሲቲ ሲሰራ 50እስከ 60 ካመጣህ ታልፋለህ እዚህኛውግቢ ግን ኣታልፍም ዚስኢስ በቃ ዘ criticall issue እችኛዋ ነገር በዚህ ብቻ በሌላ ቢሆን ንሮ ግሬዱ ለምሳሌ ኣሁን ተማሪው ሌላው ጎንደር ሌላውጅማ ኣንደኛው መቀሌ ቢደርሰው እና ስለዚ ቤተሰብ ጋር ስትሄድ ኣንደኛው ዩንቨርስቲ ከጅማ ወይም ከሌላ የመጣ ተማሪ ተመርቆ ሲመጣ ግን መቀሌ ሁኖ ደሞ ኣይደር ግቢ ቢዝነስ ኣለ አሪድም ኣለ ብዙ እንት ኣሉ ስለዚ ከኣይደር የመጣ ተማሪ ግን ኣይመረቅም

**P 6: 14_X_FGD male non-user at Ayder docx.docx - 6:77 [??? ??? ??? ??? ??? ?????? ???..] (115:115) (Super)**

Codes: [Motivators for substance use- Acadamic related - Family: 3_Motivating factors for substance use] [Motivators for substance use- Peer pressure - Family: 3_Motivating factors for substance use]

No memos

ከግቢ ውስጥ ኣንዱ ሲባል ኣንዱ የትምህርት ጫናና ሁለተኛ ደሞ peer infulence ሁለቱ ነው በኣብዛኛው እንትን የሚለው ከግቢ ውስጥ

**P 7: 15_X_IDI_ Male proctor Ayder.docx - 7:3 [??? ??????? ??? ????????? ????..] (7:7) (Super)**

Codes: [Motivators for substance use- Acadamic related - Family: 3_Motivating factors for substance use]

No memos

አሁን አጀማመራቸው ስናይ እንጠይቃቸዋለን ምንደነው የሚሉት ትምህርት ከብዶኝ፣ አዲስ ገቢ ስለሆንኩኝ ፈተና ተደራረብኝ ፈተና አለኝ ለዛነው የጀመርኩት፣

**P19: 4_IDI_ Male user BC.docx - 19:36 [plas ??? ??? class ???? ?? ???..] (96:96) (Super)**

Codes: [Motivators for substance use- Acadamic related - Family: 3_Motivating factors for substance use]

No memos

plas ደግሞ አንተ class እያለህ ቁጭ ብለህ እየቃምክ ሰትውል ለውጤት ማሸቆልቆል ምክንያት የማይሆንበት አጋጣሚ አይኖርም ሰለዚህ ችግር አለበት ማለት ነው

**P24: 9_IDI_Female proctor Ayder.docx - 24:11 [?? ?? ?? ?? ???? ?????? ????? ..] (40:40) (Super)**

Codes: [Motivators for substance use- Acadamic related - Family: 3_Motivating factors for substance use]

No memos

በቃ እኔ ዝም ብዬ ሳስበው ኣብዛኛውም የህክምን ተማሪዎች ናቸው የጥርስ ህክምና ያሉ ናቸው። እሱ ደግሞ ብዙ አመት የግቢ ቆይታ ስለሚማሩ ማለት ነው። ሳስበው ጀምረው አይደለም የመጡት በዚህ ግቢ ውስጥ ነው የከመሩት በግቢም ወዲያውኑ አይደለም የሚጀምሩት አንድ ሁለት ዓመት ተምረው ነው ወይም 3ኛ ዓመት ሲደርሱ ወይም 5ኛ ዓመት ሲደርሱ C1, C2 እያሉ ሲሄዱ ከዛ እዛ ሲደርሱ እኔ ሳስበው ትምህርት ስለሚበረታባቸው ነው፤ ራሳቸው ዘና ለማድረግ ይጀምሩታል ወይም ስለሚረበሹ ነው ብዬ አስባለሁ።

______________________________________________________________________

**Code: Motivators for substance use- Availability of the substances {34-0}**

**P 1: 1_Female Users FGD in Busness college.docx - 1:50 [??? ??? ??? ?? service sector ..] (79:79) (Super)**

Codes: [Motivators for substance use- Availability of the substances - Family: 3_Motivating factors for substance use]

No memos

ኣሁን እዚህ ከተማ ላይ service sector ስንት ነዉ።ሰርቪስ ሴክተር ይበዛል።ሰርቪስ ሴክተር ይበዛል ማለት ደግሞ የመጠጥ ቤት ይበዛል ኣሁን ስርዓቱ መጠጥ ቤት እየከፈተ መጠጥ ቤቱ የተከፈተዉ ለኛ ኣይደለንም።መጠጥ ቤት በሚበዛበት ሃገር ላይ እንዴት ነዉ ኣትጠጡ ስለሱስ የሚወራዉ።

**P 1: 1_Female Users FGD in Busness college.docx - 1:52 [?? 3? ???? ??? ??? ??? ???????..] (82:82) (Super)**

Codes: [Motivators for substance use- Availability of the substances - Family: 3_Motivating factors for substance use]

No memos

ኮድ 3፦ በግቢዉ ዉስጥ ላይፍ በራሱ ትመስለኛለች።ላይፍ በራሳችን ሃንድል ማድረግ ሲያቅተን።ከግቢዉ ዉጭ ፊት ለፊት ኣሉ የመጠጥ ቤቶች ብዙ ነገሮች ኣሉ ተማሪዉ ሲወጣ ፊትለፊት የሚታይዉ እሱ ነዉ ለምሳሌ ሙዚቃ ሊሰሙ ይችላሉ ለታክሲ ኣዉጥተህ ከምትሄድ ከተማ ሊደክምህ ይችላል ልታስበዉ ትችላለህ ግን ግቢ ወጥተህ ፊት ለፊት ኣለ መጠጥ ቤት ለምን ኣልሄድም ትላለህ እኛ እሱ ራሱ ሊያበረታታ ይችላል።

**P 1: 1_Female Users FGD in Busness college.docx - 1:89 [???? ??? ?? ?? ?? ??????? ????..] (111:111) (Super)**

Codes: [Motivators for substance use- Availability of the substances - Family: 3_Motivating factors for substance use]

No memos

ለምሳሌ ኣሪድ ግቢ እና ይህ ስናስተያየዉ ይለያያል።ለምሳሌ ኣሪድ ግቢ የታክሲ ብር የሌለዉ ሰዉ ዶርም ዉስጥ ሊዉል ይችላል።እዚህ ግን እድሉ ኣለሽ።ሲጋራ ከበር እንደወጣሽ ነዉ የምታገኚዉ።መጠጥ እንደዚሁ ለተማሪ እንደዛ ኣይደለም።

**P 1: 1_Female Users FGD in Busness college.docx - 1:91 [???? ???? ?? ???? ????? ??? ??..] (111:111) (Super)**

Codes: [Motivators for substance use- Availability of the substances - Family: 3_Motivating factors for substance use]

No memos

ከፊትሽ ስታጪዉ እና ከፊትሽ ስታገኚዉ ኣንድ ኣይደለም።እንደወጣን ጫት ቤት መጠጥ ቤት ነዉ የምናገኘዉ።እና ወጣቱ ራሱ ለዚህ ነገር ይገፋፋል።

**P 2: 10_M_IDI_Male non user BC.docx - 2:73 [?? ???? ?? ?????? ??? ???? ?? ..] (91:91) (Super)**

Codes: [Motivators for substance use- Availability of the substances - Family: 3_Motivating factors for substance use]

No memos

ሐዚ እንታይ እዩ ዘይተጠቀሰ ክብሎ ዝኽእል አብ ግቢ ዘሎ ኩነታት አወጊድና ኢና አብ ከባቢ ግብታት ዘለ ንቶም ነገራይ ንሶም ከጋልፁ ዝኽእሉ ነገራት ግን ሕጊ ብሕጋዊ ዝኾነ መንገዲ ግን ክክልከሉ አለዎም ኢለ እየ ዝሓስበ፡፡ ሐዚ ንአብነት በተለይ እንዳ ጫት፣ እንዳ ሺሻ፣ እንዳ ግዛዕ ምዛዕ

**P 3: 11_M_IDI_Male nonuser BC.docx - 3:32 [???? ??? ??? ?????? ??? ??????..] (43:43) (Super)**

Codes: [Motivators for substance use- Availability of the substances - Family: 3_Motivating factors for substance use]

No memos

አንደኛ ይሄን ነገር የሚገፋፋው ነገር በዩኒቨርስቲው ዳርዳርየ አሉ ጫት ቤቶች አሉ ሓሺሸ ቤቶች አሉ ድርማ ምናምን ነገር የሚሸጥባቸው ሲጋራ የሚሸጥባቸው ቦታዎች አሉ ይሄን ነገር እንዲያስቆሙሉን ያው ቢቆምና ይሄ ነገር አይገፋፋቸውም ከነጭራሹ ቢጦፉ ያሄን ነገር ማንም ሰው ዘወር ብሎ አይየውም እና ከዩኒቨርሲቲው ዳርዳር ያሉትን እና ይሄን ነገሮች ቢጠፋ አረፍ ነው ብዮ ነው የማስበው

**P 4: 12_MAle KIR_BC_round .docx - 4:54 [??? ???? ????? ???? DSTV ??? D..] (69:69) (Super)**

Codes: [Motivators for substance use- Availability of the substances - Family: 3_Motivating factors for substance use]

No memos

አሁን ደርፋል በሚባለው አከባቢ DSTV አለና DSTV ይሄ Normal ክላሱ ነው፡፡ ከNormal ክላሱ ትንሽ ሄድ ብሎ በቃ ሀሺሸ፣ ቁማር፣ እነዚ ምናምን እንደዚ አይነት ነገሮች አሉ ከዚ እዚ ታያለህ DSTV በሚሞላ ሰዓት እዚህም TV አለ፡፡ እዛ ትገባለህ፡፡ እና ያን የሆነ ነገሮች ሲያረጉ ታያለህ ቁማር ወይ እዛ ልትጀምር ትችላለች ቁማር ስትጀምር ደግሞ እዛ የሆኑ ያሉ ተማሪዎች የሚያጨሱ ተማሪዎች የሚያጨሱ ተማሪዎች ናቸው እና ሰብ ምናምን እያሉ ሊገፋፉህ ይችላሉ በዚያ ምክንያት ያንን ነገር ይጅምሩታል ማለት ነው ያየሁት Environment ከውጭ ይሄ ነው

**P 4: 12_MAle KIR_BC_round .docx - 4:55 [??? ?????? ??? ??? ?? ???? ???..] (73:73) (Super)**

Codes: [Motivators for substance use- Availability of the substances - Family: 3_Motivating factors for substance use]

No memos

እዚጋ በተመለከተ ደግሞ አሁን በኛ ዳርፋል የሚባለው አከባቢ ማለት ነው ወይ ደግሞ በዚህ አከባቢ መጠጥ ቤቶች አሉ ግን ዋጋቸው በጣም ውድ ነው የመጠጦች ማለት ነው እንደሰብሰቲቲውት /Substitute/ ብለው አይነት በመጠቀም ጠላ እንትን ይላሉ ጠላ ቤቶች አሁን እዛ አከባቢ በጣም አሉ በጣም ማለት ነው የነሱ መኖራቸው ማለት ነው አንድ አንድ Environment ነው እነሱን እንዲጋብዛቸው የሚያደርግ ነው፡፡ እዚ 12 ወይም 10 ከፍለፈው እንትን ከሚሉ 3 ብር ምናምን እንደዛ ዓይነት Environment መኖሩ Cost በተመለከተ ይህንን Prefer በማድረግ እንትን ይላሉ፡

**P 6: 14_X_FGD male non-user at Ayder docx.docx - 6:14 [?? ?? ???? ?? ???? ?? ??? ??? ..] (20:20) (Super)**

Codes: [Motivators for substance use- Availability of the substances - Family: 3_Motivating factors for substance use] [SU_ Trend in tearms of time - Family: 1_Commonly used substances among univeristy students]

No memos

በዛ ላይ በሁሉም በር በተለይ በዛ በላይ ያለው መንደር በጣም ወደ እንትን ተቀይረዋል የሆነች መንደር ኣለች እዛ ጋ ኣለች ካያችሃት በጣም በዛ ሻይ ቡና ለማለት ሰፈሩ ከዚህ በፊት ምንም ኣልነበረውም ዝምብሎ መኖር ቤት ነበር ኣሁን ወደ ቡና ቤት እና በጣም የጫት ቤት ኣለ ቤመጠጥ ቤት ተጨምረዋል መጠጣት ጫት ያልጀመረ almost የለም ሊባል ይችላል እና radically increase እያደረገ ነው

**P 6: 14_X_FGD male non-user at Ayder docx.docx - 6:27 [??? ??? ??? ???? ?? ??? ?? ???..] (39:39) (Super)**

Codes: [Motivators for substance use- Availability of the substances - Family: 3_Motivating factors for substance use]

No memos

ኣሁን ሶርሱ በጣም በገበያ ላይ ስላለ በቃ መግዛት ይችላሉ እንድያውም ሁሌ በቃ በዛ ሱስ የተጠቁ ተማሪዎች በቃ ብር ብር ሲሉ አንደሚጠቀሙ ያስታውቃል ታዉቃለህ ሁሌ እየዘሩ ብር እና መጠቀመቀቸው ታውቃለህ

**P 6: 14_X_FGD male non-user at Ayder docx.docx - 6:32 [?? ?????? ???? ?? ???? ?? ??? ..] (42:42) (Super)**

Codes: [Motivators for substance use- Availability of the substances - Family: 3_Motivating factors for substance use]

No memos

ሺሻ የሚጠቀሙት በከተማ ነው በከተማ ኣለ ማለት ከ ምሰማው ነው ኣለ ጫትም እንዳልከው እዚህ ማለት ኣለ በቅርቡ ኣለ

**P 6: 14_X_FGD male non-user at Ayder docx.docx - 6:33 [?? ?? ?? ??? ???? ???? ????? ?..] (42:42) (Super)**

Codes: [Motivators for substance use- Availability of the substances - Family: 3_Motivating factors for substance use]

No memos

እዚ ግቢ ውጭ ከበር ኣከባቢ ግልፅም ኣይደለም ከተማ ግን የተተፈቀደላቸው ኣንድ ኣንድ ጫት ቤቶች ኣሉ ቤት ምርጥ የባህርዳር ምናምን ጫት ቤት የሚሉ ብዙ ቦታዎች ኣሉ እና እንደዛ ኣይነት እህ ለራሴ የታዘብኩት ቦታ ስላለ እዛ ሂዶ በጅምላ ገዝተው ነው የሚመጡት

**P 6: 14_X_FGD male non-user at Ayder docx.docx - 6:34 [?? ?????? ???? ?? ?? ?? ???? ?..] (42:42) (Super)**

Codes: [Motivators for substance use- Availability of the substances - Family: 3_Motivating factors for substance use]

No memos

ቤት ተከራይተው የሚኖኑ ያው እሱ ቤት በጅምላ ብር ሲላክለት ኣንድ ሁለት ሺ ለምን በጅምላ ገዝተው ለምን ኣይመጡም የሚሉት ነገር ኣለ በኣንዴ ገዝቶዉ ይመጣሉ

**P 6: 14_X_FGD male non-user at Ayder docx.docx - 6:78 [??? ?? ?? ??? ???????? ????? ?..] (115:115) (Super)**

Codes: [Motivators for substance use- Availability of the substances - Family: 3_Motivating factors for substance use]

No memos

ከግቢ ውጭ ግን ኣሁን የዩንቨርስቲው ኣቀማመጥ ኣለ ኣብዛኛው የኢትዮጵያ የዩንቨርስቲ ኣቀማመጥ ከከተማ ወጣ ብሎ ነው መሰራት ያለበት ተማሪ እንዲያጠና ከሆነ ተማሪ እንዳይረበሽ ኣሁን የኣይደር ግቢ ስለተሰራ ይመስኛል ዝሮዝሮ የእንትን ኣቀማመጥ ኣለ ለምሳሌ እዚህ እየተጨፈረ እያለ ኣንተ ቁጭ ብለህ ለማጥናት አስቸጋሪ ነው እንደዛ ኣለ እንደገና ደሞ ቅድም ያልኩት peer infulence እያለ እንደገና የትምህርት ጫና እያለ ኣንተ በተጨናነቅክበት ስኣት ኣክሰሱ ካገኘህ ለምሳሌ ጫትacess ካገኘህ ያንን ጫት መግዛት ትችላለህ እስከ ከሌ ለግን ኣታገኘውም ያን ነገር ዝሮዝሮ ግዜውም እንትን ይላል ማለት ነው

**P 6: 14_X_FGD male non-user at Ayder docx.docx - 6:79 [??? ??? ??? ??? ???? ?????? ??..] (117:117) (Super)**

Codes: [Motivators for substance use- Availability of the substances - Family: 3_Motivating factors for substance use]

No memos

ኣንድ የሆነ ነገር የሆነ እንካን ዩንቨርስቲ ሚያክል የሆነ ትካል ለመስራት ሲታሰበ ያገርዋ ውበት ወይ ደሞ የከተማዋ እድገት infulence ካላደረገ በስተቀር መሰራት ያለበት ወጣ ባለ ቦታ ብሎ ነው ምክንያቱ ያለው ነገር ከሆነ ነገር ሴፍ መሆን ኣለበት ሳይለንት የሆነ ነገር በተለይ ይሄ ደሞኒ ከ ሆስቲታል ነው እና ብዙ ኣስታማሚ ኣለ የታመመኣለ የሚያይ ይመጣል ብዙ ኣሉ

**P 6: 14_X_FGD male non-user at Ayder docx.docx - 6:82 [????? ??? ???? ??? ????? ??? ?..] (117:117) (Super)**

Codes: [Motivators for substance use- Availability of the substances - Family: 3_Motivating factors for substance use]

No memos

በዚህኛው ጭፈራ በዛኛው ጭፈራ በዚህኛው ጭፈራ ቤት በቃ እንደ ዲም ላይት ኣለ ኣይደል ቤቱ ጎላ ኣጥፊ ያለው ልክ እንደዛ የተሰራ በዚህኛው ጭፈራ በዛኛው ጭፈራ ራሱ በቃ ቤተክርስትያን ካልሆነ በስተቀር ሂደህ እንትን ካለላልክ በስተቀር የትኛው እንትን ምትልበት የለም ምክንያቱም በዚህኛው ብትሄድ ጭፈራ ነው በዛኛውም ብትሄድ ጭፈራ ነው የትም ኣትሄድም ስለዚ ራሱ ኣቀማመጡ infulence ኣለው የሚመስለኝ

**P 7: 15_X_IDI_ Male proctor Ayder.docx - 7:34 [?? ?? ????? ???? ?/?? ??? ??? ..] (57:57) (Super)**

Codes: [Motivators for substance use- Availability of the substances - Family: 3_Motivating factors for substance use]

No memos

አዎ ደጅ ያለውን፤ የከተማ ህ/ሰብ መቼም ቢሆን አትነግድ ወይ አትስራ አትለውም ግን ተማሪው የሚፈልገው ነገር በቅርብ ማግኘቱ ግን አንድ ጥቅም ይጋብዘዋል፤ በቃ በቅርብ መገኘቱ ነው የሚመስለኝ የምናልባት የሚታቀው እንደሆነ አሪድበደጃፍ ላይ ብዙ ጫት ቤት፣ ሲጋራ ቤት ፈርሰዋል፡፡ የፈረሱበት ምክንያት ግን በቅርብ ስለነበርና ከዛ በኋላ ግን ቀንሰዋልተጠቃሚ ምክንያቱ ምንድነው እዛው ወጥተህ በቅርብ ነበር ስታገኝ የነበረው፡፡ እዚህ ደግሞ የምናልባት ዩኒቨርሲቲው በከተማ ውስጥ መሆኑ ጫና ይኖረዋል፡፡ በቅርብ ወጥተህ የምታገኝበት ዩኒቨርሲቲ ራሱ በከተማ ውስጥ ከተማ መሃል መሆኑ ጫና አለው፡፡

**P 7: 15_X_IDI_ Male proctor Ayder.docx - 7:35 [????? ?? ??? ??? ????? ??? ???..] (57:57) (Super)**

Codes: [Motivators for substance use- Availability of the substances - Family: 3_Motivating factors for substance use]

No memos

ምክንያቱ በቃ ሲጋራ ደጃፍ ትገዛለህ ማንም ከልካይ የለህም ጫትም እንደዛው አሁን ምንድነው የተጀመረው በግልፅ ማስታወቂያ ይለጥፋሉ ከዛ ውስጥ ገብተህ ቤት ነው አታቀውም ነበር ተጠቃሚ ደግሞ የት ነው የሚጨሰው፣ የት ነው የሚመረቀነው ያቁታል ስለዚህ ቅርብ መሆኑና በደቂቃዎች ወጥተህ የምትገባበት መሆፐኑ ጫና አለው፡፡

**P 7: 15_X_IDI_ Male proctor Ayder.docx - 7:36 [?? ??? ????? ???? ????? ??? ??..] (57:57) (Super)**

Codes: [Motivators for substance use- Availability of the substances - Family: 3_Motivating factors for substance use]

No memos

ሌላ ሲጋራ በደርዘን ካልሆነ በስተቀር አንድ አይሸጥም የሚል ህግ ወጥቷል፡፡ ይህን ህግ አሁን አሪፍ ነው፤ ቅድም የነገርኩክ ልምድ 20-30 ሲያጨስ የነበረው አሁን ግን ከየት ያምጣ ምንድነው ሁሉ (ደርዘን) እንዳይገዛ አቅም የለም፤ አንዷም ደሞ አተሸጥም፡፡ ስለዚህ ያለው አማራጭ ምንድነው እየቀነሰ ቀስ በቀስ መተው ነው ያለው፡፡

**P 7: 15_X_IDI_ Male proctor Ayder.docx - 7:38 [??? ??? ??? ?? ??? ??????? ???..] (61:61) (Super)**

Codes: [Motivators for substance use- Availability of the substances - Family: 3_Motivating factors for substance use]

No memos

አሁን ያለው ደግሞ ጫት በጣም የሚነገድበት ቢዝነስ ነው፤ አንድ ቀን ልጃቸው ሓኪም ነበረች፤ ነርስ ማለት ነውና የ6 ወር እረፍት አወጣች፤ ከዛ በእረፍት ግዜ ሻይ መሸጥ ጀመረች ሻይ ከጀመረች በኋላ ሁኔታው አየችና ሌላ ምን ጀመረች መሰለህ ከዛ በላይ ጫት ጀመረች፡፡ በሰዎች ትእዛዝ ማለት ይህንን ነገር የለም ይልዋትና ከዛ በግልፅ ጫት ለምን አታመጭም ይልዋታል፡፡ በዩኒቨርሲቲ ደጃፍ ነው ይህንን የሚሆነው

**P 9: 17_Male User_Adihaki campus.docx - 9:18 [????????? ???? ??? ?? ???? ???..] (42:42) (Super)**

Codes: [Motivators for substance use- abscence of alternative entertainment - Family: 3_Motivating factors for substance use] [Motivators for substance use- Availability of the substances - Family: 3_Motivating factors for substance use]

No memos

ኢንቨይሮንመንቱ አከበቢ ውስጥ ብዙ ነገሮች መዝነኛ የሉም በላይኛውም በር በሰው በደርፉር በር መጠጥ ቤቶች እና ጫት ቤቶች

**P11: 19_KIR 1_ 2nd Ineterbview_ busness compus_Mekelle University.docx - 11:16 [????? ??? ???? ???? ??? ????? ..] (21:21) (Super)**

Codes: [Motivators for substance use- Availability of the substances - Family: 3_Motivating factors for substance use]

No memos

አጠቃላይ ሚስብ ነው፡፡ አስርና አስራ ስድስትን ሙሉውን ብናየው ሙሉ መጠጥ ቤት ነውና እነዚ ነገሮች ማህበረሰቡን ወደዛ አቅጣጫ እያመሩት መሆኑ ግልፅ ነው፡፡ ና ይሀ market ኦ በራሱ ወደዚያ ይወስደናል፡፡

**P11: 19_KIR 1_ 2nd Ineterbview_ busness compus_Mekelle University.docx - 11:24 [?? ??? ?? ?????? ?? ?????? ?? ..] (39:39) (Super)**

Codes: [Motivators for substance use- Availability of the substances - Family: 3_Motivating factors for substance use]

No memos

ያው መጠጥ ቤት እየተከፈተ ነው እየበዛልን ነው ብያለው፡፡

**P12: 2_M_FGD_Male user_BC .docx - 12:10 [?? ??? ??? ??? ??? ?? ??? ????..] (25:25) (Super)**

Codes: [Motivators for substance use- Availability of the substances - Family: 3_Motivating factors for substance use]

No memos

ነኝ ስልሽ አሁን እዚጋ አሪድ ግቢ ውስጥ እንደሌላ አይደለም የሆነ ነገር ስታስቡ ቢያንስ ከተማ መወረጃ ና መመለሻ ያስፈልግሻል እና በጣም በቃ እንደመታስበው እንደሌላው ቦታ ቀላል አይደለም ለማግኘት ወጪም አለው

**P12: 2_M_FGD_Male user_BC .docx - 12:28 [????? ?? ?? ???? ????? ????? ?..] (48:48) (Super)**

Codes: [Motivators for substance use- Availability of the substances - Family: 3_Motivating factors for substance use]

No memos

ለማግኘት ራሱ ግን ከሲጋራ በይበልጥ አታገኝም በቅርቡ አታገኝም በዛ ነው ደግሞ አብዛኛው ሰው ወደ ሱስ የሚገባው ስለ ሃሽሽ አሁን ያለን በተማሪ በኩል ብናስበው ማለት ነው በቀላሉ አታገኝውም ሓሽሽ ማለት ነው ምክንያቱም ምታወጣው ብር 50 ብር ምናምን ነው እንደዛ አሁን የተወሰነ ለኛ አሁን የሁለት ሞቶ ብር አይበቃንም የ50 ብር ከመጣች አንድ ጊዜ ነው ምንጨርሳት እና በዚህ ነው በቃ እዛ ጥልቅ ብለን እዛ ሱስ ውስጥ የምንገባበት በዛ ስለሆነ ነው በቅርቡ ስለማገኝው ነው

**P13: 20_KIR 1_ 3rd round interveiew_Business Compus_mekelle Univeristy .doc - 13:10 [??? “X” ???? ???? ?? ????? ?? ..] (16:16) (Super)**

Codes: [Motivators for substance use- Availability of the substances - Family: 3_Motivating factors for substance use] [Substance used_ Khat - Family: 1_Commonly used substances among univeristy students]

No memos

*እዚህ “X” የሚባል መቃሚያ ቤት አሰውየው ግቢ ውስጥ በፕሮክተርነት ያገለግል ነበርለ፡፡ አሁን መቃያ ቤት ከፍቶል ፡፡ እናም አሁን ላይ በየቀኑ በሚባል ሁኔታ ከዛ መቃሚያ ቤት ሰው አይጠፋም፡፡ ብዙ ግዜ በቡድን ነው እንጂ ለብቻህ የሚጠጣ አለ፡፡ 3ት እና 4ት እየሆኑ ማለት ነው፡፡*

**P13: 20_KIR 1_ 3rd round interveiew_Business Compus_mekelle Univeristy .doc - 13:12 [?? ??? ??? ??? ????? ?????? ??..] (16:16) (Super)**

Codes: [Motivators for substance use- Availability of the substances - Family: 3_Motivating factors for substance use]

No memos

*ሌላ ከጠጡ በኋላ እዛው መጣላት፣ መፈረካከስ እርስ በእርስም ሊሆን ይችላል፡፡ ሌላው ደግሞ እነዚህ በዳርፏር አካባቢ የሚወጡት ጫት በጣም ይጠቀማሉ፡፡ ግቢ በሁለት ነው የሚከፈለው፡፡ የላይኛው በር የሚባለው‹‹ የሀብታሞች መውጫ› ነው የሚባለው፡፡ በዳርፏር ግን ሱቆች ናቸው ያሉት ሲጋራ የሚሸጡ፡፡*

**P13: 20_KIR 1_ 3rd round interveiew_Business Compus_mekelle Univeristy .doc - 13:14 [??? ???? ????? ????? ??? ?????..] (18:18) (Super)**

Codes: [Consquences of substance use: Financial - Family: 7_Experiance of consquences of substance use] [Motivators for substance use- Availability of the substances - Family: 3_Motivating factors for substance use]

No memos

*እናም የሱቆቹ ባለቤቶች እንዳሉኝ ከሆነ ተማሪዎች መግዣ ብር ሲያጡ እዙ ሱቅ አካባቢ ሂደው ይቆማሉ፡፡ እናውቃቸዋለን እኛ ደግሞ እንሰጣቸዋለን አለኝ፡፡*

**P14: 21_KIR 2_ 2nd interview_Busness comapas_Mekelle Univeristy.doc - 14:10 [?? ??? ?? ??????? ??? ????? ??..] (6:6) (Super)**

Codes: [Motivators for substance use- Availability of the substances - Family: 3_Motivating factors for substance use]

No memos

ሌላ ከግብ ዉች የሚገፋፋቸዉ ነገር በለፈዉም ጠቅሼዋለሁኝ ከግቢ env’t አጠቃላይ ዙሪያ እንደዚህ በመጠጥ ቤቶች የተከበበ ነዉ በቃ ዙሪያዉ በመጠጥ ቤቶች፣ ምግብ ቤቶች፣ ጫት ስጋራ ምናምን የሚሸጠባቸዉ ሱቆች በጣም ስለሚበዙ እነዛ ነገሮች መኖቸዉ አንዱ ምክንያት ነዉ ማለት ነዉ፡፡ እነዚህ ነገሮች አይቻለሁ፡፡

**P17: 24_KIR 3_ Ayder campaus Mekelle Univeristy.docx - 17:27 [??? ????? ?? ?? ??? ??? ???? ?..] (59:59) (Super)**

Codes: [Motivators for substance use- Availability of the substances - Family: 3_Motivating factors for substance use]

No memos

ካልእ ተላዒሎም ናብ ግቢ ኣቲካ ከምዚ ዓይነት ሲጋራ ምስሓብ ካልእ ማልእ ዝለዓለ እዩ እንሀ ማለት እዩ ስለዚ ኣብቲ ሕጊን ደንብንስ እውን ድኩም ከምዝኮነ ካብኡ ክትመሃርዎ ባዕሉ እውን ሓቢርካ እዙይ ንምክልካል ስለዝኮነ ወይ team ፈጢርካ ክከውን ይክእል ብደንቢ ዝስራሕ ኣካል ክገብሩ ይግባእ፡፡

**P19: 4_IDI_ Male user BC.docx - 19:29 [Plus ??? ??? ??? ????? ??? ???..] (82:82) (Super)**

Codes: [Motivators for substance use- Availability of the substances - Family: 3_Motivating factors for substance use]

No memos

Plus ደግሞ ሲመጡ ቀድም የነብረው ተማሪ ራሳቸው ቀጠይ የሚመጣ ተማሪ ይገፋፋል፡ Fresh የሚመጣ ተማሪ ። plus ደግሞ ኣከባቢው ራሱ ለምሳሌ እዚህ ጋ Darfur ብታይ ማንም እያጨሰ በተለይ ምሳ ሰዓት ኣሁን ማንም በልቶ መጥቶ እዚህ ተስልፎ ነው እያጨስ የምተየው። ስለዚህ ያ So cityው ይገፋፋሃል ወደ ኣጫሽነት።

**P20: 5_IDI_Male user-BC.docx - 20:17 [??????? ?? ??? ??? ??? ???? ??..] (39:39) (Super)**

Codes: [Motivators for substance use- Availability of the substances - Family: 3_Motivating factors for substance use]

No memos

ከዩኒቨርስቲ ውጪ ያለው ሁኔታ ከግቢ ይወጣል ከግቢ ሊወጣ ከ10 የማያንሱ ጫት ቤቶች አሉ ዳርፉር ማለት ነው በየሱቁ ሲጋራ ይሸጣል መጠጥ ቤት አለ በየቦታው እናም ያው ለሱስ የፈለገውን ነገር ማግኝት ይችላል ሩቅ መሄድመ አይጠበቅበትም ያው ተከበን ነው የምንነረው በሱስ መገኘቱ ራሱ ተማሪው ቅርብ ነው ለዚህ ሌላ የለኝም፡፡

**P20: 5_IDI_Male user-BC.docx - 20:22 [??? ?????? ?? ??? ??? ??? ??? ..] (48:48) (Super)**

Codes: [Motivators for substance use- Availability of the substances - Family: 3_Motivating factors for substance use]

No memos

ማለት የሚዝናኑት ቦታ ከግቢ ውጪው ይሁን ግቢው አከባቢ ያው በዛ ብለው ዩኒቨርሲቲ እንደመሆኑ መጠን ብዙም ሊኖሩ ይገባ ነበር ግን ለምሳሌ ይህን በርና እዚጋር ያሉትን ጫት ቤቶችን ስታሰበው ግራ ነው የሚገባህ ማለት ዩኒቨርሲቲ ሁላ አይመስልህም እንደውም ተማሪዎች የት ነው የመጣነው ሁሉ ይላሉ ሱስ ለማካሄድ ነው ወይ የመጣ ነው እስኪል ድረስ ማለት ነው እና ብዙ ምቹ ሁኔታዎች የሉም

**P22: 7_F_IDI with Female non user_BC.docx - 22:1 [?? ?? ?? ?? ?? ?? ?? ??? ?? ??..] (9:9) (Super)**

Codes: [Motivators for substance use- Availability of the substances - Family: 3_Motivating factors for substance use]

No memos

ቡዙ ግዜ ጫት ከዛ ዉጪ ደሞ ፓፍ ኣሸሽ ሺሻ ምናምን ነገሮች ይጠቀማሉ ከሌላኛዉግቢ ለእንዲህ ነገሮች ተገላጭ ነዉ ምክንያቱም ከግቢ በር ላይ እንደወጣሽ ጫት ቤቶች ኣሉ በየሱቁ ሲጋራ ቤት ኣሉ በግልባጭ ላይ እንደዚህ ስፍሮች ስትሄጂ ደሞ ሙሉ መቃሙያ ቤቶች የሺሻ ቤቶች ኣሉ እበና ተማሪዉ በቕርቡ ነዉ የሚያገኛቸዉ እንደ ወጣ ነዉ የሚያገኛቸዉ ለዚ ነገር ተገላጭ የሚሆኑት እንደኛዉ የዩንቨርስቲዉ እዚህ ከተማዉ ጫፍ መሆኑም ኣንድ ሁለተኛከግቢ ፊት ለፊት ደግሞ እነዚህ ነገሮች ኣክሰሶች ስላሉ

______________________________________________________________________

**Code: Motivators for substance use- Ceremonies & Festivities {6-0}**

**P14: 21_KIR 2_ 2nd interview_Busness comapas_Mekelle Univeristy.doc - 14:5 [??? ?? ????? ??? ??????? ??? ?..] (4:4) (Super)**

Codes: [Motivators for substance use- Ceremonies & Festivities - Family: 3_Motivating factors for substance use]

No memos

ከግቢ ዉጪ የያዘቸዉ ደግሞ ያናገርካቸዉ ደግሞ ሰዎች ያነገርካቸዉ ሰዎች እንደነገሩኝ ተማሪዎች እንዲጀምሩና ሱስን በዛ እንዲቀጠልበት የሚደረጉ ምክንያቶች አንደኛዉ በኣል በግቢ life ዉስጥ ባዓል

**P14: 21_KIR 2_ 2nd interview_Busness comapas_Mekelle Univeristy.doc - 14:6 [?? ??? ?????? ??? ?? ??? ?????..] (6:6) (Super)**

Codes: [Motivators for substance use- Ceremonies & Festivities - Family: 3_Motivating factors for substance use]

No memos

አዎ በዓል በሚኖርበት ሰዓት ብዙ ተማሪ አብዛኛዉ ተማሪ በሚሆነዉ ሁኔታ ግቢ ዉስጥ አይደለም በዓሉ የሚያከብረዉ ከግቢ ዉጭ ወጣ ብሎ ይመጣል የዛኔ ያልጣጣ በቃ በዓሉ አያከብርም ሌ ዓይነት ነገር ነዉ እንትን የሚባለዉና በበዓሉ ግዜ በጣም ብዙ ተማሪ ይወጣል ያኔ ብዙ ነገር ያደርጋል ስለወጣ በዓል የሚወጣ ብቻዉን አይደለም ከጋደኛዉ ጋር ነዉ የሚወጣዉና በሚወጣበት ሰዓት ብዙ ነገር ያያል ያን ነገር በድጋሜ ማየት ይፈልጋል፡፡

**P14: 21_KIR 2_ 2nd interview_Busness comapas_Mekelle Univeristy.doc - 14:7 [???? ??????? ????? ??? ??? ?? ..] (6:6) (Super)**

Codes: [Motivators for substance use- Ceremonies & Festivities - Family: 3_Motivating factors for substance use]

No memos

ብዙዎቹ ያናገርካቸዉ ተማሪዎች እዚህ እንደ መጡ በቃ መስከረም 16/18 አካባቢ ነዉ የሚጠራዉና እንደመጡ even እዚህ ካሉ ተማሪዎች ጋር ወይም ነባር ተማቲ ከሆኑ ያቺ በዓል ማክበር ነዉ የሚፈልጉት በቃ አሁን መስከረም 16 የሚጠራዉ አብዛኛዉ ግዜ 18 ወይም ምናምን የመስቀል አለ አንደዚህ ገና እያለ ይቀጥላልና ያ በዓል እስኪመጣላቸዉ ይቸክላሉ፡፡ በዛ የገና በዓል አክብረዉ ከሆነ የገኛ በዓል ዉስጥ ወጥተዉ ያገኙት refreshment ሌላ ግዜ ሊደገምላቸዉ ይፈልጋሉ ብር ካገኙ አሁንም ይወጣሉ ሌላ ግዜ በዛ ምክንያት እየለመዱት ይመጣሉና ያን ነገር እንደሱስ ይሆንባቸዋል ለብዙ ተማሪዎች ማለት ነዉ፡፡

**P14: 21_KIR 2_ 2nd interview_Busness comapas_Mekelle Univeristy.doc - 14:8 [??? ?????? ?management ??? ???..] (6:6) (Super)**

Codes: [Motivators for substance use- Ceremonies & Festivities - Family: 3_Motivating factors for substance use]

No memos

አሁን ያናገርኩት የmanagement ተማሪ ነበርና እስከአሁን በዓል ምናምን አይወጣም እና ዘንድሮ እንደመጣን አካባቢ ጋደኛ አለዉ እኛ ዶርም ጋር ይመጣል እሱ በጣም ነዉ የሚጠጣዉ በቃ daily ካልወጣ በቃ ዶርም ሁኖ ይደብረዋል ባህሪዉ ይቀያያራል ምናምንና እሱጋ ይዘት ወጣ እንደመጣን አካባቢ ዛሬ የሚወጣዉ ቀድሞ እሱ ነዉ በቃ 11 ወይም 12 ሰዓት ከሆነ ግድ መሄድ አለባቸዉ በቃ ማለት ነዉ እና እንደዚህ ዓይነት ያሉ ነገሮች በኣላት ብሎ የጀመረዉ በዛ ተነስቶ እንዲጀምር ያደረገዉ ዋናዉ ነገር ከግቢ ዉጭ በሱስ እንዲጠመዱ የሚደርጉ በዓላት ናቸዉ፡፡

**P14: 21_KIR 2_ 2nd interview_Busness comapas_Mekelle Univeristy.doc - 14:9 [??? ??? ??? ??? ???? ????? ???..] (6:6) (Super)**

Codes: [Motivators for substance use- Ceremonies & Festivities - Family: 3_Motivating factors for substance use]

No memos

አልፎ አልፎ አልፎ ደግሞ ሰርግም አንዳንዴ ማለት ነዉ ያየሀቸዉ ተማሪዎች አሉና ሰርግ ሳይጠሩ የሚሄዱ ተማሪዎች ዝምብሎዉ እዚህ አካባቢ ሰርግ ካለ ተማሪዎች ዝም ብለዉ ይሄዳሉ Normal በቃ ብዙ ግዜ አይከላክላቸዉም ሰርግ ቤቶች ምናምን እዛ ሄደዉ ይመጣሉ ምናምን በዛ ተነስተዉ የመጀመር ነገር ማለት ነዉ፡፡

**P22: 7_F_IDI with Female non user_BC.docx - 22:10 [???? ?? ?????? ???? ??? ????? ..] (21:21) (Super)**

Codes: [Motivators for substance use- Ceremonies & Festivities - Family: 3_Motivating factors for substance use] [Process of getting addicted (from intitiation to addiction) - Family: 5_Process of getting addited for substances]

No memos

እንዴት ነዉ የሚጀሙርት ኣንደኛ ጓደኛ መጀመርያ ከታች ክላስ ጀምሮ የመጣ ጓደኛ ካለሽ ኣዉ ምልሽ ከሱ ጋር ከሆነ ጓደኞሽን ትመስያለሽ እኔ እንዳልኩሽ የተለያዩ መንገድች ለምሳሌ ምንም ምታቅ ልጅ ዛሬ የጓደኘዋ ልደት ሁኖ ልታክብር ሂዳ ያን ነገር ልትጀምር ትችላለች እዚህ የራስሽ Life ኣለሽ የልደት ኣከባበር ላይፍ ኣለሽ ለምሳሌ በዚ ሰኣት መጠጥ ጠጥጠታ የማታዉቅ ጠጥታ ከሰከረች ኣንድ ነገር የሆነ ነገር ካጣች ለምሳሌ ያቺ ልጅ ድንግል ከሆነች ጠጥታ ራስዋን ታጣለች ከዛ በዉኃላ ኣልህ በቀልም ኣትይም ምንም ኣትይዉ ሳትፈልግ እዛ ሂወት ዉስጥ ትገባለች በእቨንነደዚህ ኣይነት ብዙ መንገድ ነዉ ያሉት

______________________________________________________________________

**Code: Motivators for substance use- Discourses {19-0}**

**P 4: 12_MAle KIR_BC_round .docx - 4:62 [Observe ?????? ??? ?? ??? ??? ..] (90:90) (Super)**

Codes: [Motivators for substance use- Discourses - Family: 3_Motivating factors for substance use]

No memos

Observe ከማድርገው ነገር እኛ ለገሱ ጭሳም ምናምን ነገር ሽታው ምናምን ነገር ይመጣሉ በቃ ጭስ ጭስ ብለው ነገር ይመርጣሉ እና ሲመጡ ወይ ትተናቸው እንወጣለን ጭስም ምናምን ነገር እንደዚህ ይባላል፡፡

**P 4: 12_MAle KIR_BC_round .docx - 4:63 [?? ?????? ??? ?? ??? ?? ???? ?..] (90:90) (Super)**

Codes: [Motivators for substance use- Discourses - Family: 3_Motivating factors for substance use]

No memos

እኛ የማንጠጣው የሆነ ፍራ የፈራ ሙድ ይዛችሁ እስከመቼ ምናምን እንደዚህ ነገር ሌላ ብዙ Observe ያደረግኩት ነገር የለም፡፡

**P 5: 13_Male KIR_BC_Round III.pdf - 5:16 [???? ????? ????? ?????? ????? ..] (4:126-4:241) (Super)**

Codes: [Motivators for substance use- Discourses - Family: 3_Motivating factors for substance use]

No memos

ሇምሳላ የሚጠቀሙ ተማሪዎች ሇማይጠቀሙ ተማሪዎች በርጫ የአሪፌ መቀመጫ የኣራዲ መቀመጫ ላሊ

መጠጥ ሇጨዋ መጫወቻ ሇባላጌ መራገጫ ነው

ኣንዴ ብርእና ኣንዴ እስኪቀረን ዴረስ እንቅማሇን

**P 5: 13_Male KIR_BC_Round III.pdf - 5:17 [?? ????? ?? ??? ?????? ?? ????..] (4:311-4:420) (Super)**

Codes: [Motivators for substance use- Discourses - Family: 3_Motivating factors for substance use]

No memos

ላሊ ተማሪዎች ምን ይሊለ ከትምህርት ጋር በማገናኘት የቃመ ተጠቀመ ያሌቃመ ተረገመ ምንዴን ነውኣብዛኛው

የማይጠቀሙ ተማሪዎች ከተጫሩ ተሇቀሙ ተባረሩ እኛ ተጠቀምን ሇማሇት ነው

**P 5: 13_Male KIR_BC_Round III.pdf - 5:18 [??? ?? ???? ?????? ?? ????? ??..] (4:423-4:496) (Super)**

Codes: [Motivators for substance use- Discourses - Family: 3_Motivating factors for substance use]

No memos

ማንስ ምን ኣገባው ወጥሬብገኝ እኔ አውቃሇሁ የጉንጬየ አቅም ራሴን ስሇማቅ አያገባቹሁም ሇማሇት ራሳቸው ሇመከሊከሌ

**P 5: 13_Male KIR_BC_Round III.pdf - 5:19 [??? ??? ??? ???] (4:499-4:513) (Super)**

Codes: [Motivators for substance use- Discourses - Family: 3_Motivating factors for substance use]

No memos

ከጠጡ ኣራት ከመቱ አራት

**P 5: 13_Male KIR_BC_Round III.pdf - 5:21 [?? ???? ??? ?? ???? ??????? ??..] (4:520-4:751) (Super)**

Codes: [Motivators for substance use- Discourses - Family: 3_Motivating factors for substance use]

No memos

ቅሞ ከማዘን ብሌጥ ነው መመዘን የማይቅሙትን ተማሪዎች ምንዴንነው ኣብዛኛዎቹ የሚቅሙ ተማሪዎች በጥሳቸው ኣከባቢ

ወይም በ ኣካሊቸው ስታያቸsiው በሰውነታቸው አከባቢ እንዯዚህ እየቀጨጨ እየቀነሰ ይሄዲሌ እና በዛ ብትመዘኑ ይሻሊሌ

የሰውነት ክብዯታቸው ከጫት ይሌቅ ብትበለ ይሻሊሌ

የሰውነት ክብዯታቸው ነው ወይስ የጫቱ ነው

ከጫት ይሌቅ ብትበለ ይሻሊሌ ሇማሇት ነው

**P 5: 13_Male KIR_BC_Round III.pdf - 5:22 [??? ????? ????] (4:754-4:767) (Super)**

Codes: [Motivators for substance use- Discourses - Family: 3_Motivating factors for substance use]

No memos

በርጫ የሀሪፍች መቀመጫ

**P13: 20_KIR 1_ 3rd round interveiew_Business Compus_mekelle Univeristy .doc - 13:17 [????? ???? ????? ????? ????? ?..] (22:22) (Super)**

Codes: [Motivators for substance use- Discourses - Family: 3_Motivating factors for substance use]

No memos

*አባባሎቹ የሚቅሙ ተማሪዎች ለማይቅሙ ተማሪዎች የሚዛልፉ ደግሞ የማይቅሙ ተማሪዎች ደግሞ ለሚቅሙት የሚናንሱበት ማለት ነው፡፡ ተጠቃሚ ተማሪዎች ለምሳሌ ‹ በርጫ የሀሪፍ መቀመጫ› ይላሉ፡፡ ሌላው ‹ መጠጥ ለጨዋ መጫወቻ ለባለጌ መራገጫ ነው›፡፡ ሌላ ደግሞ ቃሚዎቹ ‹ አንድ ብር እና አንድ ጥርስ እስከሚቀረን ድረስ እንቅማለን፡፡*

**P13: 20_KIR 1_ 3rd round interveiew_Business Compus_mekelle Univeristy .doc - 13:18 [?????? ??? ?? ????? ??? ?? ???..] (22:22) (Super)**

Codes: [Motivators for substance use- Discourses - Family: 3_Motivating factors for substance use]

No memos

*ከትምህርት ውጤት ጋር በተገናኘ ደግሞ ሱስ ተጠቃሚ ተማሪዎች ‹‹ ያልቃመ ተለቀመ የቃመ ተጠቀመ ›› ይላሉ፡፡ አብዛኞቹ የማይቅሙ ተማሪዎች ከግቢ ሲጫሩ ተለቀሙ ከግቢ ተባረሩ ለማለት ፣ እኛም ቅመን ተጠቅመናል ለማለት ተፈልጎ ነው፡፡*

**P13: 20_KIR 1_ 3rd round interveiew_Business Compus_mekelle Univeristy .doc - 13:19 [?? ??? ??? ?? ???? ??? ???? ??..] (22:22) (Super)**

Codes: [Motivators for substance use- Discourses - Family: 3_Motivating factors for substance use]

No memos

*ሌላ ደግሞ ማንስ ምን አገባው ወጥሬ ብቅም፣ እኔ አውቀዋለህ የጉንጩን አቅም፡፡*

**P13: 20_KIR 1_ 3rd round interveiew_Business Compus_mekelle Univeristy .doc - 13:20 [??? ???? ??? ‹‹??? ?? ????….››..] (22:22) (Super)**

Codes: [Motivators for substance use- Discourses - Family: 3_Motivating factors for substance use]

No memos

*አሁን የቅድሙ ጥቅስ ‹‹ማንስ ምን አገባው….›› የሚለው አባባል ስሌለው ሰው ደንታ የለንም አንጨናነቅም ወጥረን እንቅማለን እንጂ ለማለት ነው፡፡ ሌላ ሰው ከሚል ሳይን የራሴን አቅም የማውቀው እኔ ስለሆንኩኝ ሌላ ቦታ የለኝም ለማለት ነው፡፡*

**P13: 20_KIR 1_ 3rd round interveiew_Business Compus_mekelle Univeristy .doc - 13:21 [????? ??? ?? ??? ???? ?????? ?..] (22:22) (Super)**

Codes: [Motivators for substance use- Discourses - Family: 3_Motivating factors for substance use]

No memos

*በተጨማሪ ደግሞ ምን ያክል መጠጣት እንዳለብህ ለመግለፅ ‹ከጠጡ አራት ከመቱ አናት›› ይላሉ፡፡ ይሄ ምን ማለት ነው አራት እንኳን የማይጠጣ ተማሪ ተማሪ አይደለም ለማለት ነው፡፡*

**P13: 20_KIR 1_ 3rd round interveiew_Business Compus_mekelle Univeristy .doc - 13:22 [??? ??? ?????? ????? ??? ‹‹ ??..] (22:22) (Super)**

Codes: [Motivators for substance use- Discourses - Family: 3_Motivating factors for substance use]

No memos

*ሌላው ደግሞ የማይቅሙት ተማሪዎች ደግሞ ‹‹ ቅሞ ከማዘን በልቶ መመዘን›› ይላ፡፡ አብዛኛው ግዜ የሚቅሙ ሰዎች ሰውነታቸው የቀነሰ እና ደከም ያሉ ናቸው እና ያንን ለመግለፅ ብትበሉ ይሻላችኋል ከሞትቅሙ ለማለት ነው፡፡*

**P14: 21_KIR 2_ 2nd interview_Busness comapas_Mekelle Univeristy.doc - 14:43 [?? ????? ????? ???? ???? ??? ?..] (28:28) (Super)**

Codes: [Motivators for substance use- Discourses - Family: 3_Motivating factors for substance use]

No memos

ሌላ አንዳንድ አባባሎች እንትን ለማለት ምክሬ ነበር አሁን ለምሳሌ "ጫትን በተመለከተ ጫት ሙቀት እንጂ እውቀት አይጨምርም"

**P14: 21_KIR 2_ 2nd interview_Busness comapas_Mekelle Univeristy.doc - 14:44 [???? ?? ?????] (28:28) (Super)**

Codes: [Motivators for substance use- Discourses - Family: 3_Motivating factors for substance use]

No memos

ሰካራም ቤት አይሰራም

**P14: 21_KIR 2_ 2nd interview_Busness comapas_Mekelle Univeristy.doc - 14:45 [??? ?? ??? ??? ???? ?????? ???..] (30:30) (Super)**

Codes: [Motivators for substance use- Discourses - Family: 3_Motivating factors for substance use]

No memos

አሁን ጫት ሙቀት እንጂ እዉቀት አይጨምርም ከእነዚህ ተማሪዎች ከማይቀሙት ተማሪዎች፡፡ የማይጠቀሙት ተማሪዎች እነሱን ነዉ የሚላቸዉ እነሱማ የሆነ ነገር perceive አድርገዋል ማለቴ ተጠቅመን ራሳችን እንጠቅማለን ዉጤት ለማሻሻል ብሎ አስበዉ ነዉ የሚገቡት ወይ ራሳቸዉ ለማዝናናት ብለዉ ነዉ የሚገቡት

**P19: 4_IDI_ Male user BC.docx - 19:41 [“??? ??? ??? ???? ??? ????? “ ..] (114:114) (Super)**

Codes: [Ceaseing : Intention to cease - Family: 8_Intention to cease and experiance of relapse for substance use] [Motivators for substance use- Discourses - Family: 3_Motivating factors for substance use]

No memos

“ኣጫሽ ወይም ሱሰኛ ያርፋል እንጂ ኣይተውም “ የሚባል እንትን ኣለና ያ ልጅ ተመልሶ እነዛ ግን እስከሁን ቀጥለዋል።

**P20: 5_IDI_Male user-BC.docx - 20:21 [???? ????? ??? ???? ??? ???? ?..] (44:44) (Super)**

Codes: [Consquences of substance use: Pshychological distress - Family: 7_Experiance of consquences of substance use] [Motivators for substance use- Discourses - Family: 3_Motivating factors for substance use]

No memos

ለምሳሌ በመቃሚያ ሰዓት መገላል የለም መገለል በኖርማል ላይሆን ሱሰኛ ያልሆኑት በPsychological ለመጉዳት ይመክራሉ ቀጥታ ሱሰኛ ነህ ብለው አይናገሩም ስነልቦናዊ እና በሌላ ነገር የበታችነት ማሳየት ማለት ነው፡፡

______________________________________________________________________

**Code: Motivators for substance use- Educational system it slef {6-0}**

**P 1: 1_Female Users FGD in Busness college.docx - 1:12 [?? ????? ??? ??/? ???? ???? ??..] (39:39) (Super)**

Codes: [Motivators for substance use- Educational system it slef - Family: 3_Motivating factors for substance use]

No memos

ግን መጀመርያ ነገር የት/ት ስርዓት ኣሰጣጡ ራሱ ለዚህ ነገር ምቹ ነዉ።

**P 1: 1_Female Users FGD in Busness college.docx - 1:27 [?? 3:- peer pressure ?????? ??..] (58:58) (Super)**

Codes: [Motivators for substance use- Acadamic related - Family: 3_Motivating factors for substance use] [Motivators for substance use- Educational system it slef - Family: 3_Motivating factors for substance use] [Motivators for substance use- Peer pressure - Family: 3_Motivating factors for substance use]

No memos

ኮድ 3- peer pressure እንዳላቸዉ ሊሆን ይችላል።ኑሮ ሲከብዳቸዉ ይመስለኛል ተማሪዉ ወይም ት/ት ሊከብደዉ ይችላል ወይም ላይፍ ግቢ ላይ ያለዉ ላይፍ ለምሳሌ ኣንድ ሴት ደምኛ ከተደረገች ተናዳ የምትጀምረዉ ሊሆን ይችላል እንጂ ደስ ብሎት የሚጀምር ሰዉ ይኖራል ብዬ ኣላስብም።

**P 1: 1_Female Users FGD in Busness college.docx - 1:46 [?? ???????? ???? ??? ????? ???..] (77:77) (Super)**

Codes: [Motivators for substance use- Educational system it slef - Family: 3_Motivating factors for substance use]

No memos

እኔ ከኣስተማሪዎች ከማማዝ በላይ መንግስት ነው። ልክ እኛ መንግስት እኛ የሚፈልገን ለቁጥር ነው ለማማላት ነው።እኛም እንደቁጥር እናሟላላን በየዓመቱ ይህን ያክል ቁጥር ያስመርቃል። ምንም የሚያታግለን የለም መፅሓፍ ለ47 section ያደረሳችሁ እንባላለን።ለነገ ፈተና ዛሬ handout ይሰጠናልበፈለገ ሰዓት ገብቶ በ37 አንደ ዩኒት ሁለት ዩኒት ጨርሶ ተፈትኖ ይላል።ማስተማር ያልፈለገውን ቻፕተር assignment ብሎ ይሰጠናል።ይሄ ሁሉ አስተማሪው እንደፈለገው ግሬድ ይሰጣል አስተማሪው ማን ስጋራ ብሎ ተቆጣጥረው መጥቶ አንድቀን የወሩን አቴንዳስ ፈሪሙልኝ ይለና እንፈርማለን ስርዓቱ እኛ ቁስ ነን እኛ ይህ ደግሞ ህይወት ውስጥ ይገባል ትርጉም ኣልቦ ህይወት የሚባለው ይገባል

**P 1: 1_Female Users FGD in Busness college.docx - 1:48 [???? ????? ????? ?? ??????????..] (78:78) (Super)**

Codes: [Motivators for substance use- Educational system it slef - Family: 3_Motivating factors for substance use]

No memos

ተምረን አይደለም አንብበን ነው የምንፈተነው።ደግሞ የአቀባበል በነገራችን እኩል ኣይደለም ደረጃ አለው በዛ treat ኣይደረግም የሚገባውም የማይገባውንም ሁሉ እኩል ነው።

**P 1: 1_Female Users FGD in Busness college.docx - 1:49 [?????? ?????? ?? ??? ???? ??? ..] (78:78) (Super)**

Codes: [Motivators for substance use- Educational system it slef - Family: 3_Motivating factors for substance use]

No memos

ኢንተርኔት ኣገልግሎት ኣለ ብለን ብናስብ ግቢዉ ዉስጥ የለም እኮ።ለእኛ እንድንጠቀምበት ቢሆን ምን ያክል ተማሪ ኢንተርኔት ኣግኝቶ እየተጠቀመ ነዉ።ኣለ በር ተዘግቶ እየተጠቀምንበት ኣይደለም።መፃህፍት ኣናገኝም።የማንበብ ልምዳችን እንዲበረታታ ኣያደርግም።ይህ ሁሉ ነገር ጭንቅላታችን እየተሰራ ኣይደለም ይህ ከመሰረቱ ነዉ ሁሉም ተማሪ ወደዚህ የሚገባዉ።

**P24: 9_IDI_Female proctor Ayder.docx - 24:14 [?? ????? ??? ?? ????? ??? ?? ?..] (50:50) (Super)**

Codes: [Motivators for substance use- Educational system it slef - Family: 3_Motivating factors for substance use]

No memos

ግን ምንድነው ደግሞ ሌላ የሰማነው ዘበኛ በር አይዘጋም፤ አሁን እንጃ አላቅም፤ አቴንዳንስ ደግሞ ሙሉ ሌሊት ክፍት ነው፤ ቱያዙ አልተያዙ ትርጉም የለውም ምክንያቱም ደስ ያላቸው ስራ ሰርተው ይመጣሉ ማንም አይዛቸውም በር ላይ፤ ስራ አላቸው ተብሎ ስለሚታሰብ ሁሉም እንኳን ባይሆኑ ተረኛ ነኝ ነው የሚሉህ።

______________________________________________________________________

**Code: Motivators for substance use- Excess pocket money {2-0}**

**P 4: 12_MAle KIR_BC_round .docx - 4:31 [?? ??? ??? ????? ???? ??? ??? ..] (39:39) (Super)**

Codes: [Motivators for substance use- Excess pocket money - Family: 3_Motivating factors for substance use]

No memos

እዚ ከመጡ በኃላ የቤተሰብ ቁጥጥር የለም ሌላም ቦቃ ብርም ሊነሱ ተብሉ በየወሩ ወይም ደሞ በየሣምንቱ የሚበጀትላቸው ብር ስለለ በዚህ ምክንያት እንትን ይላሉ፡፡

**P18: 3_M_FGD_Male User's _Ayder.docx - 18:16 [???? ??? ??? ????? ??? ????? ?..] (40:40) (Super)**

Codes: [Motivators for substance use- Excess pocket money - Family: 3_Motivating factors for substance use]

No memos

ገንዘብ ትሓደ ብዙሕ ትሰደደሉ ትሓደ ንእሽተይ ተሰዲድሉ አይደለም ትዋና ታይ እዩ አነ ሽሕ ቅርሺ ተልኢኩለይ ጓደኛየ 500 ብር ተተላኢኹሉ አነ ትሱሰይ ታይ ይገብሮለኹ የግፋሕለኹ የዕብዮለኹ አነ ኸዚ 10 ቅርሺ ተሃሊያትኒ ብዛ 10 ቅርሺ ክቅሕም ይኽእል እዩ 500 ብር ካለኝ የ500 ነገሮች እዩ ልእዝዝ ማለት እዩ ና200 ጫት ይቅሕም ናይ 200 ይሰቲ ማለት እዩ፡፡

______________________________________________________________________

**Code: Motivators for substance use- Excessive free time {8-0}**

**P 1: 1_Female Users FGD in Busness college.docx - 1:9 [1:-??? ???? ?/? ?? ???? ??? 9 ..] (39:39) (Super)**

Codes: [Motivators for substance use- Excessive free time - Family: 3_Motivating factors for substance use] [SU_ Time to use subatnce- Afternoon - Family: 2_Conveneient time and place to use susbstance]

No memos

1-ኣሁን ለምሳሌ ት/ት ቤት እያለን እስከ 9 ሰዓት ት/ት ነን ሰለዚህ ከ930 በኃላ ነዉ።እዚህ ግን 230-430 ከዛ በኃላ ነፃ ነን። የ2 ሰዓት ብቻ ሊሆን ይችላል የሚኖረን እሱም ላይኖር ይችላል። እና ሙሉ ቀን ዶርም ዉስጥ ቁጭ ማለት ነዉ።ግቢዉ ዉስጥ ደግሞ ምንም የተዘጋጀ ነገር የለመ ተማሪዉ ሊያሳልፍባቸዉ የሚችሉ።ስለዚህ ኣብዛኛዉ ሰዓታችን ነፃ ነን

**P 1: 1_Female Users FGD in Busness college.docx - 1:11 [?/? ??? ???? ?2 ?? ???????????..] (39:39) (Super)**

Codes: [Motivators for substance use- Excessive free time - Family: 3_Motivating factors for substance use]

No memos

ት/ት ደግሞ ቢያንስ ከ2 ወር ኣይጀምርም።ከመጠን በላይ ማለት ነዉ።ያ ሰዓት ደግሞ ወደዛ እንድንገባ ያደርገናል፤በጣመ ነፃ የሆንክበት ግዜ ማለት ነዉ።ከዛ እዛጋ የለመድከዉ ት/ት ተጠናክሮ ሲጀምር ከሱስ መዉጣት ይከብደናል

**P 1: 1_Female Users FGD in Busness college.docx - 1:39 [assignment ????? ?exam prepara..] (65:65) (Super)**

Codes: [Motivators for substance use- Excessive free time - Family: 3_Motivating factors for substance use]

No memos

assignment ይሰጠናል ለexam preparation ይሰጠናል።ግን assignment ሰርተን ለእንደዚህ ዓይነት የምናጠፋቸው ብዙ ጊዜዎች ኣሉ። ጊዜ ማሳጠሩ ሳይሆን lesson ስለማይኖር ነው። ኣናቅም እንደዚህ ኣይነት እንደምንገባ።

**P11: 19_KIR 1_ 2nd Ineterbview_ busness compus_Mekelle University.docx - 11:15 [?? ?????? ??? ?? ??? ??? ?????..] (18:18) (Super)**

Codes: [Motivators for substance use- Excessive free time - Family: 3_Motivating factors for substance use]

No memos

እና አብዛኛውን ሰዓት ደሞ ትርፍ ሰዓት ይበዛዋል ትምህርት ራሱ ማይጀመርበት ሰዓቶችም አሉና ቁም ማለትም ያስጠላል፡፡ እና እነዚ ሁለት ምክንያቶች ናቸው ግን ከዚ ደሞ ተጨማሪ ምንላቸው ወደ ሱስ ይገቡበታል ብየ እኔ…….

**P16: 23_KIR 2_1st interveiw_Busness campus Mekelle Univeristy.doc - 16:13 [???? ??? ????? ??? ??? ?? ????..] (127:127) (Super)**

Codes: [Motivators for substance use- Excessive free time - Family: 3_Motivating factors for substance use]

No memos

በግሬድ ደረጃ የምትዩን ከሆነ አሁን በኔ ልንገርሽ እኔ ት/ት ላይ አስራሰሁ ከበፊትም ቤዛ/base/ አለንኝ አስራለሁ ግን ዛሬ እኔ ቀኑን ሙሉ አለጫለኩም አሁን ነው የመጠሁት አሁን እንደንዴ እኔ ለምሰሌ አክስቴ ቤት ምናምን እሄደለሁ አይደለ የዞን ታይም ላይ ሳምንቱን ሙሉ ሳለጨስ እቀመጠለሁ ግን ኖርማሊ እዚ ከሆንኩ ፍሬ /free/ነኝ ምንም አሰረግም ምናምን አጭስ ይሰኛል አጫስለሁ እንደ ወዳበሪያ ነነ ያሰሃል፡፡

**P16: 23_KIR 2_1st interveiw_Busness campus Mekelle Univeristy.doc - 16:18 [Code.3 ?????? ??? ????? ??????..] (130:130) (Super)**

Codes: [Motivators for substance use- Excessive free time - Family: 3_Motivating factors for substance use]

No memos

Code.3 የኒቨርሲት ውስጥ ለመጠቀም የሚያበታታ ነገር ወዳ ሱስ የሚገፈታ ነገሮች አሁን ድብርት አለ ግቢ ውስጥ ድብረቱን እንት ለማረግ ዳም አሁን ያልኩሽ ነገር ምንጠቃምበቸው ማለት እና ጫት አነሱ በቃ ቀን ማሰለፍያ የሆነሉ

**P17: 24_KIR 3_ Ayder campaus Mekelle Univeristy.docx - 17:6 [??? ????? ?? ????? ?? ???? ?? ..] (22:22) (Super)**

Codes: [Motivators for substance use- Excessive free time - Family: 3_Motivating factors for substance use]

No memos

ካምቲ ዝበልኩካ ከዓ ኣጀሚርዋ ከም መሕለፊ ግዜ ሱቅ ኢለካ በቃ ዝተልዓለ በተለይ ካብ ትምህርቲ ዝተበራረ ዝረከብክዎ ዝጀመረሉ ምክንያት ክትሪኦ ከለካ ካብ ት/ቲ ስለዝተባረረ በቃ መውዓሊ ይስእን ዳሓር ደገ ከይዱ ስዋ ይሰቲ ፣ ሲጋራ ገለ ይስሕብ ከመኡ እናበለ ብዙሓት ዓይነታት ኣልኮል ፣ ሲጋራ ፣ ጫት ናብ ኩሎም ተደሪስዎም ኣሎ ማለት እዩ መሕለፊ ግዜ እናበለ ማለት እዩ፡በናይ ትምህርቲ ድክመት ዝተለዓለ ዝጀመረሉ ኩነታት ውን ኣሎ፡፡

**P18: 3_M_FGD_Male User's _Ayder.docx - 18:3 [??? ???? ?????? ??? ?? ???? ??..] (6:6) (Super)**

Codes: [Assumed purpose to strat substance use- Concentration - Family: 4_Percieved importance of subatance use by users] [Motivators for substance use- Excessive free time - Family: 3_Motivating factors for substance use]

No memos

ልግዜ መህለፊ ልመፅናዕቲ ልሓደ ሐደ ነገራት ትጥቀመሉ ዳአ እምበር ሱስ ብስሩ አስፈላጊ አይደለም እዩ

______________________________________________________________________

**Code: Motivators for substance use- Expermentation {5-0}**

**P 1: 1_Female Users FGD in Busness college.docx - 1:83 [????? ??? ???? ????? ??? ?? ??..] (103:103) (Super)**

Codes: [Motivators for substance use- Expermentation - Family: 3_Motivating factors for substance use]

No memos

ሲጀምሩት ላይክ ላይፉን ሊሞክሩት ብለዉ ነዉ የሚጀምሩት ብዬ ኣምናለዉ።ጥቅሙ ከመጀመሩ በኋላ ነዉ የሚያዉቁት።ሲጀምሩት ምንድነዉ ብለዉ ይጀምሩታል።ቴስቱን ማወቅ ስለሚፈልጉ እናም ጥቅሙን ማወቅ ስለሚፈልጉ ይጀምራሉ።

**P 2: 10_M_IDI_Male non user BC.docx - 2:5 [???? ?? ??? ???? ?? ?? ???? ??..] (10:10) (Super)**

Codes: [Motivators for substance use- Expermentation - Family: 3_Motivating factors for substance use]

No memos

ክጥዕም ገለ እስኪ ክምክሮ ብኡ ብኡ ገይርካ ትጥቀም ነገር አሎ::

**P21: 6_F_IDI with Female Non User at Ayder.docx - 21:7 [??? ????? ??? ??? ??? ?? ?????..] (41:41) (Super)**

Codes: [Motivators for substance use- Expermentation - Family: 3_Motivating factors for substance use]

No memos

ኩሎም ዝጥቀሙሉ ጥቅሚ ኣለው ኢሎመ እካ ኣይኮኑን ዝጥቀሙሉ ግን ሱቅ ኢሎመ እንዲዒ ሓንቲ ነይራማሓዛይ እካ ክነግረካ ምስ ካልኦት ማሓዙታ ወፂኣ ንደገ ክንሰቲ ኢላእና ሰኪራ ምስ ሰከረት ዝኮነ ዝሳሓብ ሲጋራ ምሰ ራኣየት እዚ ሲጋራ እንታይ እነታይ ከም ዝጥዕም ዘይጥዕሞ ኢላ ጣዓመቶ ንምንታይ ኣነ ኸ ዘይጥዕሞ ኢላ ኩሉ ሰብ ይሰቲ ምስ ራኣየት ሰኪራ እያ ብዘይ ልባ እያ ዝኒሃ ውኒኣ ሲሒታ ያ ካብኡ ብኡ ነይራ ጀሚራ ሹዑ ካብቲ ቆልዓ ተቀቢላ ለኩሰለይ ኢላ ሰትያ ።ኣነስ ሐሲባቶ ወይ ጥቅሚ ኣለዎ አላ ዘይኮን ሰትያስ ሰብ ይገብር ኣሎ ወይ ከኣ ብዘይ ሐሳብ ብ ተንሺን ይኩነ ኻሊእኻሊእ እውን ይጅምሮ ሞ መደሓር ሱሰ ይታሓዝ ።

**P22: 7_F_IDI with Female non user_BC.docx - 22:3 [???? ?????? ???? ???? ?????? ?..] (14:14) (Super)**

Codes: [Motivators for substance use- Expermentation - Family: 3_Motivating factors for substance use]

No memos

የገጠር ማህበረሰብ ከገጠር መጥተዉ የሚያዉቁት ነገር ሲፈልጉ ለዛ ነገር ተገላጭ ይሆናሉ እያወቁትም ለማወቅ

**P24: 9_IDI_Female proctor Ayder.docx - 24:15 [I. ?? ??? ??? ?????? ??? P. ??..] (52:53) (Super)**

Codes: [Motivators for substance use- Expermentation - Family: 3_Motivating factors for substance use]

No memos

I. ውጭ ማደር ለሱስ አስተዋፅኦ አለው
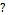


P. አዎ! አለው ብየ ነው የማስበው ማለት ስታስበው ወጭ የማድር ከሆንኩኝ እየጨፈርኩኝ ነው የማድረው፤ መጠጥም ይኖራል፤ ሲጋራ ማጨስም ይኖራል፤ ስለዚህ ሁሉ የሚጨፍር ለሱስ የተጋለጡ ናቸው ባንልም አንዳንዶቹ ግን ያጋልጣቸዋል። ይሸወዳሉ ብየ ነው የማስበው ለመሞከር ይቸኩሉ ይሆናል።

______________________________________________________________________

**Code: Motivators for substance use- Family related {3-0}**

**P 3: 11_M_IDI_Male nonuser BC.docx - 3:29 [???? /Family/ ?? /Case/ ??? ??..] (41:41) (Super)**

Codes: [Motivators for substance use- Family related - Family: 3_Motivating factors for substance use]

No memos

*ባለፉት እንዳልኩት ነው ከቤተሰብ የሚመነጩ ችግሮች ደግሞ አሉ፡፡ በቤተሰብ ውስጥ ስለተፈጠረ ክስተት በማሰብ ከዛ ነገር ለመውጣት ጓደኛ ጋር እንትን ብለ ወደ ሱስ የሚገቡበት ሁኔታ አለ፡፡*

በፋሚሊ /Family/ ኬዝ /Case/ ሊሆን ይችላል ፋሚሊ አሁን በደንብ ለተማሪው ብዙ ካልተላከለት ያንን ለመረሳት የሚያደርገው ነገር ሊሆን ይችላል የሆነ አለ አይደል አንድ ነገር ሊያምረው ይችላል ብዙ ነገር መብላት እያለበት ብዙ ነገርም ላይበላ ይቸላል ተይጠጠም ይችላል እና ወጣትም ስልሆነ እናም ግቢ ውስጥ ላይፍም ብዙ ነገር ስላለ እናም ብዙ ካልተላከለት ወዳዛ ነገር ውስጥ ነው የሚገባው ብዮ ነው የማስበው

**P 4: 12_MAle KIR_BC_round .docx - 4:49 [?? ????? ??? ??? ? ????? ?? ??..] (58:58) (Super)**

Codes: [Motivators for substance use- Family related - Family: 3_Motivating factors for substance use]

No memos

ሌላ ከቤተሰብ ወይም ወላጅ ጋ በተያያዘ ነው እዚ አንዳንድ ተማሪዎች የሚጀምሩት ቤተሰብ እዚ ሆነው እነሱ ወይ ሞቶባቸው ወይ ታምባቸው ምናምን እንደዚህ ዓይነት ጭቀት ነገር ሲሰሙ በቃ ወደ ዛ ነገር ይገፋፋችዋል፡፡

**P13: 20_KIR 1_ 3rd round interveiew_Business Compus_mekelle Univeristy .doc - 13:9 [???? ?????? ?? ????? ????? ???..] (14:14) (Super)**

Codes: [Motivators for substance use- Family related - Family: 3_Motivating factors for substance use]

No memos

______________________________________________________________________

**Code: Motivators for substance use- Felling of inferiority {4-0}**

**P 4: 12_MAle KIR_BC_round .docx - 4:51 [?? ??? ???? inferiority ??????..] (62:62) (Super)**

Codes: [Motivators for substance use- Felling of inferiority - Family: 3_Motivating factors for substance use]

No memos

ግቢ ውስጥ እንዴት inferiority የሚሰማቸው ተማሪዋች አሉ በክላስ ውጤታቸው ለሆን ይችላል በመጡበት ቦታ ምክንያት ምናምን እንደዚህ ነገር የሚሰማቸው ተማሪዎች ናቸው More ማለት ነው፡፡

**P 4: 12_MAle KIR_BC_round .docx - 4:52 [??? ?? ????? ??? ??????? ?? ??..] (62:62) (Super)**

Codes: [Motivators for substance use- Felling of inferiority - Family: 3_Motivating factors for substance use]

No memos

አሁን ብዙ ተማሪዎች ሀሺሽ የሚጠቀሙትን እዚ ግቢ ማለት በቃ በቁጥርም አናሣ ናቸው እና ለማወቅም እንትን ብዮ ነበር እና በቃ ወይ ጥርሣቸው የተበላሸ ሊሆን ይችላል ወይ ደሞ ፊታቸው ያህን ያክል አይደለም እና በዚያ influence ሴቶችን አሁን አይቻቸው ነበርና እና በቃ እንትናቸውን ሰታያቸው በቃ እና ያማችዋል

**P 8: 16_FGD ARID_Users .docx - 8:42 [?? ??? ????? ???? ?????? ?? ??..] (81:81) (Super)**

Codes: [Motivators for substance use- Felling of inferiority - Family: 3_Motivating factors for substance use]

No memos

እኔ ከሚን አንሰለሁ ጓደኛዬ እየተጠቆመ እኛ ለምንድነው ማስጠቀመው የለል

**P16: 23_KIR 2_1st interveiw_Busness campus Mekelle Univeristy.doc - 16:7 [???? ?? ??? ??? ?????? ??? ???..] (121:121) (Super)**

Codes: [Motivators for substance use- Felling of inferiority - Family: 3_Motivating factors for substance use]

No memos

ሒዚኣነ ኣብ ስራሕ ኽይዳ ኣይስሕብን ኣብዚ መፃአ አየ ዝጃምር ማለት ዩ ግን አንታይ ነስለካ ዝኛነ ዘጨናንቐካ ነገር እሆ ከፍቲምግ በርሱቲ ነይ ግቢ ምግቢ እቲ ቐርሺ ሀፍቲ ዘይምምጥጠን እኒአ ትሓዳ ወድ ሃፍታም ትብድኸነት ርንክል ዝብል እኒሆ ከብዚ ተብገሉ ሱቐ አልካ በቃ ለወ

______________________________________________________________________

**Code: Motivators for substance use- following frendship breack up {7-0}**

**P 1: 1_Female Users FGD in Busness college.docx - 1:28 [F:- ??? ???; ???? ?? ????? bre..] (59:60) (Super)**

Codes: [Motivators for substance use- following frendship breack up - Family: 3_Motivating factors for substance use]

No memos

F- ደምኛ ስትይ;

ለምሳሌ ቦይ ፍሬንድዋ break up ካደረገች ተናዳ እንደዛ ሊሆን ይችላል።

**P14: 21_KIR 2_ 2nd interview_Busness comapas_Mekelle Univeristy.doc - 14:60 [??? ?? ?????? ??? ??? ???? ???..] (53:53) (Super)**

Codes: [Motivators for substance use- following frendship breack up - Family: 3_Motivating factors for substance use]

No memos

እዚህ ላይ የታዘብኩት ሁለት ነገር አለ፡፡ አንዳንድ ያናገርካቸዉ ተማሪዎች ነበሩ ግንኙነት ከነበራቸዉ በኃላ እዚህ እንድ ዩኒቨርሲቲ ተመድበዉ ማለት ነዉ እዚህ ከመጠች በኃላ እሷ ሌላ ጓደኛ ስትይዝ በዛ ብስጭት የጀመረ ተማሪ አለ፡፡

**P14: 21_KIR 2_ 2nd interview_Busness comapas_Mekelle Univeristy.doc - 14:62 [????? ??? ?? ????? ??? ????? ?..] (55:55) (Super)**

Codes: [Motivators for substance use- following frendship breack up - Family: 3_Motivating factors for substance use]

No memos

አብዛኛዉ መጠጥ ነዉ ለመናገር መጠጥ ይጠቀማሉ ግን ደግሞ ፍቅረኛቸዉ እንደዚህ ያጡ ተማሪዎች ጠይቀዉ እምቢ የተባሉ ተማሪዎች ማጨስ ላይ more ይሳተፋሉ፡፡

**P14: 21_KIR 2_ 2nd interview_Busness comapas_Mekelle Univeristy.doc - 14:63 [???? ??? 2nd Year ???? ??? ?? ..] (59:59) (Super)**

Codes: [Motivators for substance use- following frendship breack up - Family: 3_Motivating factors for substance use]

No memos

እንግድ አምና 2^nd^ Year እያለን እዚህ ግቢ ራሱን ያጠፋ ተማሪ አለ ነበረና በፍቅር ነዉ ሌላ ጋደኛ ይዛበት ምናምን እንደዚህ ይጠጣል በብስጭት ግለት ነዉ ኢቭን እስከነረኪና ድረስ ጠጥቶ ነበረ በጣም እንትን በማለት ራሱ እስከማጥፋት የደረሰበት ደረጃ ነበረ፡፡ እሳ ጋር ፍቅር ላይ እያለም እምቢ ስትለዉም ብዙ ነገር ይጠጣል፡፡ ያጨሳል እንዲህ ነገር ይፈራልና ይህን ነገር ነዉ observe ነዉ ያደረኩት

**P17: 24_KIR 3_ Ayder campaus Mekelle Univeristy.docx - 17:2 [???? ??????? ?? ????? ???? ?? ..] (12:12) (Super)**

Codes: [Assumed purpose to strat substance use- Relief from anxity - Family: 4_Percieved importance of subatance use by users] [Motivators for substance use- following frendship breack up - Family: 3_Motivating factors for substance use]

No memos

ኣበዙይ ዝረከብክዎም ምስ ፍቅረኛካ ምብኣስ ኣሎ ካልእ ካዓ ካብ ቤት ት/ቲ ምብራር ንኣብነት ተባሪርካ እናሃለካ ግን ካብዚ ግቢ ከይለቀቁ ይከዱ ግን ኣበዙይ ኮይኖም ተጠቀምተ ይኩኑ ካብቲ ት/ቲ ለቂቆም ኣለው ኣበይ ይውዕሉ ኣለዉ? አብኡ ግን ካብቲ ት/ቲ ዓለም ስለዝተባረሩ እዮም ኣበኡ ዝወዕሉ ዘለው ማለት እዩ ከምዚ ዓይነት ረኪበ ኣለኩ ግን መበገሲኡ ናይ ገሊኦም ካብ ካብ ት/ቲ ዓለም ምብራር እዩ ናይ ገሊኦም ከዓ ምስ ፍቅረኘኦም ዝተበኣሱ ዝርከብክዎም ኣሎ፡፡

**P19: 4_IDI_ Male user BC.docx - 19:45 [?? ??? ????? actually ?? ??? ?..] (124:124) (Super)**

Codes: [Motivators for substance use- following frendship breack up - Family: 3_Motivating factors for substance use]

No memos

ሌላ ጋደኛ ትይዛለህ actually ብቻ መጥፎ ነግር ከዚህ የዘለለ ሱስ የምለስ እኔ በራሴ። እንድዚህ Chick የምንለው ነገር ኣለ ጋደኛ ያለ ኣቅምህ ትይዛለህ ዝም ብለህ ቆንጆ ስለሆነች ብቻ ኣንተነትህን ሳታቅ እካ ትይዛለህ እነሱ በብር ዛሬ ብር ነው ወሳኙ ፍቅር የሚባለው ንገር I don’t know እኔ ኣላምንበትም ስለዚህ ያንተ ብር ከሽቆለቆለ በኃላ ያቺ ልጅ ሌላ ትፈልጋለች already ስለዚህ academically ምትምታለህ ሂወትም የትጋደሉም ኣሉ ራሱ እካ ያጠፋ በግቢያችን ዓይናችን እያየን በቢላና ይህ መጥፎ ነገር ኣለ ስለዚህ በተማሪዎች እንደዚህ ዓይነት ፍቅር ሲያስፍልግህ እኩያህ ላንተ የምትሆን መምረጥ ኣለብህ ምክንያቱም ብዙ ተማሪዎች በዚህ ይጎዳሉ።

**P19: 4_IDI_ Male user BC.docx - 19:46 [??? ???? ?? ??? ???? ?? even ?..] (124:124) (Super)**

Codes: [Motivators for substance use- following frendship breack up - Family: 3_Motivating factors for substance use]

No memos

ለርጎ የሚጠጣ ኣለ መርዝ የሚጠጣ ኣለ even ወደ መጠጥ የሚያገፋፋህ ኣብዛኛው ፍቅር ነው by the way ዝምብለህ ልትሆንህ ወይም እያወቅክ እካ even ለትፈልግህ እያለች ግግም ብለህ ትሂዳለህ ኣልፈልግህም የሚል መልስ ሲምጣ ወደላልሆነ ነገር ትገባለህ እንደዚህ ዓይነት ነገሮች ኣሉ ያለነሳናችው።

______________________________________________________________________

**Code: Motivators for substance use- helplessness {18-0}**

**P 1: 1_Female Users FGD in Busness college.docx - 1:35 [????? ??? ??? ?? ???? ???? ???..] (63:63) (Super)**

Codes: [Motivators for substance use- helplessness - Family: 3_Motivating factors for substance use]

No memos

ሃይስኩል ንደዛ ነገር ላይ ብትታይ ቤተሰብ ሊጠራ እና ኣስተማሪም ኣለ። ካምፓስ ላይ ግን እንደዚ ኣይደል።ኣስተማሪዎቻችን እንካን እንደዚ ኣይነት ሲያዩን ሊረዱን ከሚሞክሩ ይልቅ ግሬዳችን የሚያበላሹበት ግዜ ይበዛል እና በዚህ ዓይነት ሁኔታ ዉስጥ ሆኖ ቤተሰብ የምንናገርበት ግዜ ኣይደለም።ትልልቅ ልጆች በራሳችን ሃንድል ማድረግ ኣለብን ተብሎ ነዉ የሚገመተዉ።መዉጫችን መንገድ መሸሸግያ የሚመስለን ኣልኮል ነዉ።

**P 1: 1_Female Users FGD in Busness college.docx - 1:38 [????? ??? ????? ????? ????????..] (65:65) (Super)**

Codes: [Motivators for substance use- helplessness - Family: 3_Motivating factors for substance use]

No memos

ሃይስኩል ሲሆን በራሳችን ኣይደለም የምናድገዉ።በጊዜ የምንገባበት እዚህ ላይ ስንመጣ ግን እንደምናስበዉ ኣይደለም ካምፓስ ላይ ሃይስኩል ላይ easy ነዉ።ኣስተማሪ ሲያስረዳህ ስለ ግሬድህ እየተጨነቀ ምንድነዉ።እዚህ ግን የማየዉ ነገር ኣስተማሪዎቻችን ስለእኛ ላይ የሚጨነቁበት ያንያህል ኣይደለም።ብንጫር እንካን ሳፖርት የሚያደርገን የለም።ኢ/ያ ዉስጥ ላይፍ ከባድ ነዉ።ከእንደዚህ ኣይነት ወጥተህ ቤተሰብ የምትናገርበት የለም።ኣልኮል መፍትሄ ነዉ ብለን የምናስበዉ weakness ቢሆንም ኣማራጭ መስሎ ስለሚታየን ነዉ።

**P 1: 1_Female Users FGD in Busness college.docx - 1:40 [?? ??? ????????? support ?????..] (69:69) (Super)**

Codes: [Motivators for substance use- helplessness - Family: 3_Motivating factors for substance use]

No memos

ብዙ ነው። አስተማሪዎቻችን support ሊያረጉን ይገባል። አንዳንዴ ላይፎቻችን ruin የሚያረጉት እነሱ ናቸው ሳስበው ማለት ነው። support አያረጉንም። ለምሳሌ መጠጥ ቤት ብንታይ ሁሉም የሚመስለው መቅበት ነው።ላይፍ ተመቻችቶልን ብር ኑሮን ስለዚህ ነገ ክላስ ላይ ብትታይ ለት/ት ፍላጎተ ያለህ ነው ተብሎ አይታሰብም።ችግራችን ጠርቶ አያናግሩህም። በቃ ከዚህ ነው ላይይፋችን መበላሸት የሚጀምረው ማለት ነው።

**P 1: 1_Female Users FGD in Busness college.docx - 1:47 [????? ??? ?????? ?????? ??? ??..] (78:78) (Super)**

Codes: [Motivators for substance use- helplessness - Family: 3_Motivating factors for substance use]

No memos

ወለጆችን እዚህ የሚገጥሙን ኣያውቁም። በቀን ሶስት (3) ሰዓት እንደምንማር ኣያውቁም ቢያውቁ ኑሮ አይልኩንም ነበር።ት/ት ቤት ላይ መምግሮቻችን እንዴት እንደሚቆጣጠሩን ያውቃሉ።እዚህም እንደዛ ይመስላቸዋል ምን ያህል ስርዓቱን እኛ ላይ ዘግናኝ እንደሆነ ይዘነግታል።

**P 1: 1_Female Users FGD in Busness college.docx - 1:54 [?? ?? ??? ?? ????? ??? ?? ?? ?..] (83:83) (Super)**

Codes: [Motivators for substance use- helplessness - Family: 3_Motivating factors for substance use]

No memos

ግን እዛ ማንም ኬር የሚሰጠዉ የለም ስለ እኛ ላይፍ መበላሸት እንመረቅ እንመረቅ ቢያንስ እንኳ ለኛ በያዝኑ እንኳ የቤተሰብ ባክግራዉንግ እንኳ ታይቶ የቤተሰብ እርዳታ መስጠት ኣለ።ማንም ለእኛ የሚያስብልን የለም ግቢ ላይፍ ነዉ ለዚህ ሁሉ የሚዳርገን በእርግጠኝነት ማለት ነወ።

**P 1: 1_Female Users FGD in Busness college.docx - 1:55 [?? ???? ????? ????? ??? ????? ..] (84:84) (Super)**

Codes: [Motivators for substance use- helplessness - Family: 3_Motivating factors for substance use]

No memos

እኛ ተጠያቂ ኣይደለን ለራሳችን ሂወት መበላሸት እዛጋ ማንም እያገዘን ኣይደለም ማንም ሊረዳንእሚመክር የለም።just pretending ብቻ ነዉ።we really care ይላል ግን ማንም ኬር የሚሰጥ የለም።

**P 1: 1_Female Users FGD in Busness college.docx - 1:56 [???? ??? ?? ???? ????? ?? ????..] (85:85) (Super)**

Codes: [Motivators for substance use- helplessness - Family: 3_Motivating factors for substance use]

No memos

በግቢዉ ላይፍ ስል ከክላስ ይጀምራል እኛ ሃይስኩል ላይ ቀለል ኣድርገን ኣጥንተን ገብተን እንሰራለን ኣስተማሪ ያግዘናል የሃይስኩል ኣስተማሪ ማለት ኣባት ነዉ መጨረሻህ እንዲያምር ይፈልጋል።ግቢ ዉስጥ ግን ከ100%- 99% ማንም ኬር የሚያደርግ የለም።ገብተን ተመርቀን መዉጣታችን ነዉ።ምንም ሳያዉቅ ራሱ ኮርጆ 3-4 ይዞ ሊወጣ ይችላል።ኣጥንቶ ግን ባለበት ችግር ብቻ መስራት እየፈለገ መስራት ሳይችል በወደቀ ዉጤት ወጥቶ ስራ የማያገኝ ኣለ።ያ ነዉ ፋክቱ በቃ ኣስተማሪዎቻችን እኔ እንጃ ፀጥ ብለዉ ለማስተማር ይሰጣቸዋል እድሉ እንጂ እዛ ፖዝሽን ላይ ይመጥናሉ ተብሎ እንደሚገመገሙ ኣይታየንም።

**P 1: 1_Female Users FGD in Busness college.docx - 1:57 [???? ????? ?? ??? ???? ?????? ..] (86:86) (Super)**

Codes: [Motivators for substance use- helplessness - Family: 3_Motivating factors for substance use]

No memos

ስለዚህ የቻልነዉ ሱስ ዉስጥ ገብተን የቻልነዉን ያህል ተመርቀን ግን ስንት ኣሉ ላይፍ ተበላሽቶ ለዘልኣለም የሚቀር። በትክክል ኣስተምሮን ግን ስንት ኣለ በትክክል የማያስተምር።እሱ ሳይገባ የኣንድ ወር ኣስፈርሞ እኛ 2 ቀን ስንቀር ግን ፋይናል እንዳንፈተን ያደርገናል።ያ ነዉ ሲስተሙ ማለት፤

**P 1: 1_Female Users FGD in Busness college.docx - 1:58 [?? ???? ??? ??? ?????? ?????? ..] (87:87) (Super)**

Codes: [Motivators for substance use- helplessness - Family: 3_Motivating factors for substance use]

No memos

ከዛ የዘለለ ነገር ከእኛ ኣይፈልጉም መምህራኖቹ ኣስተማሩ የሚባለዉ ነዉ የሚፈልገዉ ቢሮ ላይስለዚህ አነሱ ክላስ ገቡ ወጡ እነሱ የሚጠቅምባቸዉ A B C ብለዉግሬድ ማስቀመጣቸዉ ነዉ። ለነሱ ያ ነዉ የዘቺ ያስፈረሙባት ኣቴንዳንስ ከዛ መጨረሻ ቀን ወጥቶ የሚበተንልን ወረቀት ኣለ ግምገማ ሙሉ ይባላል።በግምገማዉ ምንም ነገር ተሰርቶበት ኣያዉቅም።በተሃደሶ የተለያየ ዓይነት ስብሰባ ይመጣል።ተማሪዉ ሃሳቡን ይገልፃል ግን ምንም ለዉጥ የለዉም።እኔ የትምህርት ስርዓቱ መበላሸት ለተማሪዉ መበላሸት ዋና ነዉ ተጠያቂም ነዉ።

**P 1: 1_Female Users FGD in Busness college.docx - 1:59 [???? ??? ?0.5 ???????? ?? 0.5 ..] (87:87) (Super)**

Codes: [Motivators for substance use- helplessness - Family: 3_Motivating factors for substance use]

No memos

ለምሳሌ ኣሁን በ0.5 ትጫራለህ።ግን ያቺ 0.5 ኣስተማሪዉ ሲያርም ስህተት ትሆናለች።ግን የትኛዉ ፈተና ወረቀት ነዉ እኛችን ገብቶ ያየነዉ።እጃችን የገቡ ፈተናዎች ላይ ስንት ኣስተካክለናል።እኔ ለምሳሌ 10 ማርክ ጨማሬ ኣግኝቻለሁ እኔ ጋር ፈተናዉ ሲደርስ ማለት ነዉ።ያቺ 0.5 ብዙ ተማሪ ታስጭራለች።ያቺ 0.5 የኣስተማሪዉ ክላስ ኣለመግባት ሊሆን ይችላል።እዉኔቴን ነዉ ሁለት ቻፕተር በ2 ሰዓት ጨርሶልን ይወጣል።ይሄ ሁሉ ኣለ ምንም ነገር እየተማርን እኮ ኣይደለም።

**P 1: 1_Female Users FGD in Busness college.docx - 1:60 [??? ?? ??? ????? ?? ??????????..] (87:87) (Super)**

Codes: [Motivators for substance use- helplessness - Family: 3_Motivating factors for substance use]

No memos

ግሬድ ነዉ እኛም ግሬዳችነ ነዉ የምንፈልገዉ።እነሱም ደግሞ ያቺ ግሬድ እስከሚያስቀምጥዋት ነዉ የሚሮጡት።እኛን ማስተማረ ሃሳባቸዉ ኣይደለም።እኔ ለምሳሌ ኢኮኖሚክስ የኢኮኖሚክስ ጭብጥ ሃሳብና እኔ ነገ ከዚህ ወጥቼ ስለ ኢኮኖሚክስ ትንታኔ ትሳለች ኣትሰጥም ኣይደለም እኔ እዛጋእየተፈተሽኩ ያለሁት።ግሬድዋ 2 ሞልትዋል ኣልሞላም ነዉ። A B C ነዉ ጨዋታዉ ከዛ ዉጭ ምንም ነገር የለም።ቁጥር ነዉ ያልኩት ይሄ ነዉ ሌላ ነገር ኣይደለም።

**P 6: 14_X_FGD male non-user at Ayder docx.docx - 6:63 [????? ????? ??? ??? ???? ??? ?..] (85:85) (Super)**

Codes: [Motivators for substance use- helplessness - Family: 3_Motivating factors for substance use] [Recommendations to adress motivating factors: Adminstartaive related - Family: 3_Motivating factors for substance use]

No memos

ኣብዛኛው ተብሎዋል ትንሽ ለየት የምትል ነገር ኣላት ኣሁን የነዚህ ጋር የእነዚህ ምክንያት እና መንስኤ የሚሆነው የadministration የዚህ ግቢ የ administration ን የኣስተዳደር ለተማሪ ያላቸው ትኩረት ከኣስተማሪ ከላይኛውም ጀምሮ እስከታችኛው ያለ በቃ እዚህ ያለ ተማሪ ነገ ህዝብ ሀገር የሚረከቡ ህዝቡ የሚያገለግል እንደ ኣንድ ዜጋ ተቆጥሮ I donot know ምንም ለመግለፅ ኣትችልም እነሱ ማለት ኣብዛኛው በቃ የሆነ የራሳቸው ተብትነት ኣለ ሴትከሆነች የራስዋ የሴትነት ምታያይዝበት ወንድከሆነ የራሱ በቃ የየራሳቸው በቃ ኣንድ ቢሮ ሁነው የሆነህ ኣንድ ችግር ለመፍታት ኣይስማሙም በቃ ያኛው የራሱ ይይዛል ያኛውን እንደዛ ተማሪ በዚህ ይጉላላል ለኣንዲት የሆነች ችግር ለመፍታት ሁለት ኣመት መጣም የሚያሳዝን ማለት ነው

**P 8: 16_FGD ARID_Users .docx - 8:17 [????? ???? ?? ???????? ?? ????..] (25:25) (Super)**

Codes: [Motivators for substance use- helplessness - Family: 3_Motivating factors for substance use]

No memos

ከቤተሰብ መለየት ራሱ እንዳፍለጎትህ ነው የምትሆነው ሚል

**P 8: 16_FGD ARID_Users .docx - 8:29 [Code 3 ????? ??? ???? ???? ???..] (49:49) (Super)**

Codes: [Ceaseing : Inetreventions exist to help ceasing - Family: 8_Intention to cease and experiance of relapse for substance use] [Motivators for substance use- helplessness - Family: 3_Motivating factors for substance use]

No memos

Code 3 መመካከር የለም ኣትቃም ኣትጠጠ የሚለኝ ሰው የለም አንዲያውም እንድ ሲቆም ሊያጨስ ያየውና ለምንድን ነው ስትል ነቆተህ ለፈተና እንድትደርስነው የሉህና እነሱን ትክተለለህ ፡፡

**P16: 23_KIR 2_1st interveiw_Busness campus Mekelle Univeristy.doc - 16:20 [???? ??? ???? ?? ????? ??? ???..] (130:130) (Super)**

Codes: [Motivators for substance use- helplessness - Family: 3_Motivating factors for substance use]

No memos

ቤታቸን ሆነን እዳዚህ ሱስ እናበዛም እንሽ ቀነስ እራረጋለን እዚ ፍረደም የለው ደግም የት እንደምታድ ምንምን ማንም የሚጠየቂህ ሰው የለም ብር ብቻ ከየዝክ ማደሪየ ብቻ ከልህ እየስከርስ ጥቃት ልትመጣ ትችለለህ 8፡00 ሰዓት ትመጠልለህ የሚቆጠጠር ሰው ስለሌለ ማለት ነው በዛ ነው በቃ ሱስ ውስጥ እንትን ምነሰው

**P24: 9_IDI_Female proctor Ayder.docx - 24:24 [?? ????? ?? ????? ??? ?? ??? ?..] (86:86) (Super)**

Codes: [Motivators for substance use- helplessness - Family: 3_Motivating factors for substance use]

No memos

ከዛ እንደዚህ ብሎ ተበላሽቶ የዚህ ግቢ ችግር ደግሞ መተሳሰብ የለም፤ አንድ ተማሪ እንደዛ ቢሆን እንኳን ስቆ ነው የሚያልፈው እንጂ ተማሪዎች ህብረት አሉ፤ ፕረዚዳንት የተማሪዎች አለ ግን አይተባበሩም፤ ከዛም ቤተሰብ በቅርብ የለም፤

**P24: 9_IDI_Female proctor Ayder.docx - 24:25 [????? ????? ????? ??? ????? ??..] (86:86) (Super)**

Codes: [Motivators for substance use- helplessness - Family: 3_Motivating factors for substance use]

No memos

ተማሪዎች ህብረት፣ ፕሮክተር ነበር መከታተል የሚችለው አሁን ግን እነዚህ ሰዎች በጣም ደካማ ናቸው አይከታተሉም፤ ተማሪው እብድ ሆኖ ነው የሚታየው፤ ከፍተኛ ደረጃ እስከሚደርስ የሚከታተል የለም፤

**P24: 9_IDI_Female proctor Ayder.docx - 24:30 [????? ??? ???? ??? ???????? ??..] (99:99) (Super)**

Codes: [Motivators for substance use- helplessness - Family: 3_Motivating factors for substance use]

No memos

ከተወሰነ ዓመት በኋላ፤ ማንም የሚከታተላቸው የለም መምህራንም፣ ተማሪዎች ህብረት፣ ጓደኛው እያጨሰ የሚያየው ከሆነ እሱም ጤና የለውም፤ መምከር አለበት ለማስተው፤ ከመጀመሪያ ከሆነ ጥሩ፤ ፍላጎትም ይኖረዋል፤ ጠበቅ አርገህ ከያዝከው በጣም ሳይለምደው ማለት ነው፤ አሁን ግን ለተማሪ ችላ የማለት ነገር አለ፤ የሚጨነቅለት የለም፤ ተማሪው በቃ ተበላሽተው፣ ተለማምዶ ከነገሮች ጋር ወይም አቋርጦ ይሄዳል ወይም ሳይኮሎጂካሊ ይጎዳል፤ ሳይካትሪክ ይሆናል እና ማንም ትኩረት የሚያረግበት የለም፤ እኛ ፕሮከተሮች እንሻላለን ብየ አስባለሁ፤ ፕሮክተር ስለሆንኩ ሳይሆን ከኛ ቅርብ ስላሉ መሰለኝ ስናያቸው የተወሰነ እንመክራቸዋለን፤ መቼ ጀመርከው፣ ምን የረግልሃል ተማሪ አይደለህም እንዴ ምክር እየሰጠን ተማሪዎቹ በቃ የተለመዱ ቢሆንም በመጠኑ እየመከርናቸው ነው፤

______________________________________________________________________

**Code: Motivators for substance use- hopless in educational carrier {1-0}**

**P 2: 10_M_IDI_Male non user BC.docx - 2:57 [??? ?? Friend ??? ????? ???? ?..] (69:69) (Super)**

Codes: [Motivators for substance use- hopless in educational carrier - Family: 3_Motivating factors for substance use]

No memos

ካብኡ ከዓ Friend ካልእ አእሚንዎ ዝመፅእ እቲ ማይንዱ ግቢ ክመፅእ ከሎ እነታይ ክገብር እዩ ዝመፅእ እንታይ ሓሱቡ አቂዱ ዝመፅእ ሐዚ ግቢ ክበሃል ከሎ ስሙ ከምዚ ካሊእ ወዲ ሃፍታም እንተኮይኑ ሱቅ ኢሉ ዕብድ ክብል ከም ድልየቱ አብኡን እንኡን ይከታተልዎ ነይሮም ሐዚ ንሱ ከይጭቅጭቅዎ እናበለ እዩ እምበር ካሊእ አቂዱ ይመፅእ ወይ አጋላት ገላ ይሕዝ ክብል ይክእል አና እቲ ኪነታት ሓደ ግቢ ክበሃል ከሎ ሰም እውን እዩ ሐዚ ስም ምስቶም ዝሕቡኻ ሐዚ ከከም ደረጃ ንሪኡ እቲ ተምሃራይ ዓሊሙ ዝመፀሉ ኩነታይ አሎ ወዲ ሃፍታም ካብ ትምህርቲ ተወሳኪ እንዲያም ንምዝንነዘይ ይመፅእ፡፡ ወዲ ድኻ ተመሪቆ ስራሕ ክቁፀር ወዲ መካካለኛ ውን ዝሮ ዝሮ ኩሎም ተመሳሰልቲ እዮም ምስቲ ድኻ ኩሎም ንዕላማ እዩም፡፡

______________________________________________________________________

**Code: Motivators for substance use- hoplessness {4-0}**

**P 1: 1_Female Users FGD in Busness college.docx - 1:32 [??????? ??????? ?????? ?? ????..] (62:62) (Super)**

Codes: [Motivators for substance use- hoplessness - Family: 3_Motivating factors for substance use]

No memos

ኣብዛኞቻችን በቤተሰባችን ኣስተሳሰብ ነዉ ተገድበን የነበርዉ ልክ እዚ ስትመጣ ከቤትህ ይዘሀዉ የመጣህ ነገር ይሰበራል።እና ኣንድ እዉቀትህን ሰብረህ ጥለሀዉ ሌላዉን ልታገኝ ስትሄድ ባዶነት ኣለ።ያ ትርጉም ኣልባ ማለት ነዉ ማህበረሰቡ የሰጠን ነገር ኣለ።እዛ challenge ኣርገን ኣልመጣንም እዚህ ነዉ challenge ያደረግነዉ።እና ከቤተሰቡ ይዘነዉ የመጣነዉ ይሰባበራል።ኣዲስ ነገር ለመገንባት ስትሄድ ደግሞ ኣዲስ ነገር እስክትገነባ ሰፊዉ ማህበረሰብ ለመልመድ የሚያደርግህ ምንም ነገር የለም።የምታደርገዉ እንድትደበቅ ነዉ።እንድትደበቅ ደግሞ ሰፊ ነገር ኣለህ ማለት ነዉ።ለምሳሌ ትንሽ ሲከፋህ ብዙ ነገር ኣጠገብህ ኣለ።ትርጉም ኣልባ ነገር ደግሞ መሸሸግያ ነዉ። ምትፈልገዉመ መጠጥ ምናምን ደግሞ ይሸሽግናል።ስለዚህ ወደዛ ይገባል ማለት ነዉ።

**P 1: 1_Female Users FGD in Busness college.docx - 1:34 [???? ?? ????? ????? ??? ???? ?..] (63:63) (Super)**

Codes: [Motivators for substance use- hoplessness - Family: 3_Motivating factors for substance use]

No memos

ካምፓስ ላይ ምንድነዉ ትናንትና እቤት ሳፖርት እየተደረግን የቆየን ልጆች በኣንድ ቀን እዚህ ስንገባ ሁሉም ነገር ይለወጣል።በኣመቺ ሁኔታ ሱስ ዉስጥ የገባ እና ሳያዉቀዉ ላይፍ ሳይመቻችለት የገባ ለመዉጣት እንኳ እኩል ኣይደለም።ተመቻችቶለት የሚገባዉ ለመዝናናት ነዉ።ኣልኮል ለመዝናናት የሚሆነዉ እስከ ተወሰነ ግዜ ነዉ።ከዛ በኃላ ግን ሳንፈልገዉ እንደጋግመዋለን።ለመዉጣት ይከብዳል

**P11: 19_KIR 1_ 2nd Ineterbview_ busness compus_Mekelle University.docx - 11:12 [?? ??? ?? ??? ?? ??? ?? ??????..] (15:15) (Super)**

Codes: [Motivators for substance use- hoplessness - Family: 3_Motivating factors for substance use]

No memos

ግን አሁን እሱ ፍሬሽ ላይ ያለው ይሀ ለመዝናናትና ምናምን ነገሮች መላመድ ብቻ ነው ሰኔርና ጅሲ ላይ ነው ያለው እሱ ግን ተስፋ ከመቁረጥ የሚመጣ ነው፡፡ ብዙሃኑ ማለት ነው፡፡ የይህወት አብዛኛውም ነገሮች ወደዛ ነገር እንድገባ የሚያረገው የህይወት ፍልስፍና ነው፡፡ እና የሕይወት ፍልስፍና ደግሞ የአብዛኛውም ያው ምንም ነገር ባዶ ነው፡፡

**P11: 19_KIR 1_ 2nd Ineterbview_ busness compus_Mekelle University.docx - 11:13 [??? ??? ??? ???? ?? ?? ??? ???..] (15:15) (Super)**

Codes: [Motivators for substance use- hoplessness - Family: 3_Motivating factors for substance use]

No memos

ይህን ይህን ስትል ስምበዛ እሱ ነው ማለት ነው፡፡ ፍልሴ የህወት ፍልስፍናቸው ሲጀመሪም አብዛኛውን ተማሪ እንደዛ ነው፡፡ ሁሉም ነገርን ትርጉም አልባ ማረግ ምናም ከዛ የሀ ኩሾቹን ምናምን ደግሞ substans ኦቹ እነዛ ነገር በጣም proof ያረጉታል፡፡ እዛ ይበልጥ ይገቡበታል ማለት ነው፡፡

______________________________________________________________________

**Code: Motivators for substance use- Media and advertisment[alcohol] {4-0}**

**P 1: 1_Female Users FGD in Busness college.docx - 1:51 [??? ?10 ???? ??? ??? ???advert..] (80:80) (Super)**

Codes: [Motivators for substance use- Media and advertisment[alcohol] - Family: 3_Motivating factors for substance use]

No memos

ኣሁን ከ10 ዓይነት በላይ መጠጥ ኣለ።advertisment እንዳለ መጠጥ ነዉ።መንግስት መቼ ተቆጣጠረዉ ታድያ እኛ ላይ በተዘረጋልን ትቦ እንሄዳለን።ሆን ብሎ እየተደረገ ያለዉ ነዉ የሚመስለዉ።

**P 6: 14_X_FGD male non-user at Ayder docx.docx - 6:87 [???? ?? ??? ?????? ???? ??? ??..] (126:126) (Super)**

Codes: [Motivators for substance use- Media and advertisment[alcohol] - Family: 3_Motivating factors for substance use]

No memos

ሁለተኛ ደሞ መቀሌ ዩንቨርስቲ በኣንድ ኣንድ ያለው እየከለከለ ነው ሱስ ተዉ ምናምን እያለ ነው ኣንድ ኣንድ ብታየው ደሞ ኣንደኛ ስፖንሰር እየሆነ ነው በ concert በቀደም የነበረው concert ኣንደኛ ስፖንሰር መቀሌ ዩንቨርስቲ ነው ለሌላ ማስተማር የነበረበት እሱ ግቡ እያለ ነው ኣሁንም እዚህ ጋር ትኬት ይሸጣል የconcert ውጭ እደሩ እያልክ ነው ኣሀን እዚ ጋር ግቢ ውስጥ መሸጥ ማለት ግቢ ውስጥ መለጠፍ ማለት የኮንሰርት ውጭ እደሩ ለሱስ እስኪ ተጋበዙ ማለት ነው እዛ ሂደህ ጥሩ ኣታይም ሁሉም ያጨሳል እዛጋር ቢራ ኣለ እዚህ ጋር ኣሁን ኣንደኛ ስፖንሰር ዋልያቢራ ምናምን የሚል ኣለ ያላል በታች እና እዛጋር ኮንሰርት ብትሄድ ቢራ ኣለ ሲጋራም ሊኖር ይችላል እና እዛውጭ ባደሩበት የሆነ ኢፌክት ይኖሮዋል እና መቀሌ ዩንቨርስቲ ይገፋፋልም ይከለክላልም በሌላ እና ይሄ ቢታሰብ

**P 6: 14_X_FGD male non-user at Ayder docx.docx - 6:89 [????? ??? ?? ?? 5 ??? ??? ????..] (129:129) (Super)**

Codes: [Motivators for substance use- Media and advertisment[alcohol] - Family: 3_Motivating factors for substance use]

No memos

እንዳለው ጓደኛ ደሞ ኮድ 5 ያለው ደሞኒ ኣልኩት መጀመርያ ኣሁን ለምሰሳሌ ላይ administration የመቀሌ ዩንቨርስቲ ኣንደኛ ስፖንሰር ሌላ ነገር sponser ማድረግ እያለበት ለሙዚቆኛች ለቢራ ለዳሽን ነፍ ነው ኣሁን ለምሳሌ ስንት ችግር በተማሪ መስራት ያለበት ይህን ነገር ለመፍታት ኣንድቀን አመቻችቶ መቸም ያንን ስፖንሰር ሲያደርግ የሚወጣው ወጪ ይታወቃል ስለዚ ምን ችግር እንዳለ ወይም እንዴት ኣድርገን መስራት እንዳለብን ብሎ እንትን እንዳይል sponser በቃ ኣንደኛ ስፖንሰር ሳይ መቀሌ ዩንቨርስቲ ነው በቃ መሆን የለበትም

**P 6: 14_X_FGD male non-user at Ayder docx.docx - 6:90 [sponser ???? ??? ?? ???? ?? ??..] (129:129) (Super)**

Codes: [Motivators for substance use- Media and advertisment[alcohol] - Family: 3_Motivating factors for substance use]

No memos

sponser ኣንደኛ የራያ ቢራ የዳሽን ቢራ እንትን ሲባል እንዳለው ብትሄድ መቀሌ ዩንቨርስቲ ብትሄድ መቀሌ ዩንቨርስቲ ስለዚ እሱ ሌላ ነገር እያለ የጤና ሳይንስ እየተባለ ሃገር የሚመሩ እንትን እያስተማረ እየተባለ ስንት ነገር የሚጠቀምበት በእነዚህ እስፖንሰር እያለ እንትን የሚል ከሆነ አለ የህብረተሰብ ስለዚ እንትን ራሱ እንትን ያደርግሃል እና

______________________________________________________________________

**Code: Motivators for substance use- Peer pressure {83-0}**

**P 1: 1_Female Users FGD in Busness college.docx - 1:27 [?? 3:- peer pressure ?????? ??..] (58:58) (Super)**

Codes: [Motivators for substance use- Acadamic related - Family: 3_Motivating factors for substance use] [Motivators for substance use- Educational system it slef - Family: 3_Motivating factors for substance use] [Motivators for substance use- Peer pressure - Family: 3_Motivating factors for substance use]

No memos

ኮድ 3- peer pressure እንዳላቸዉ ሊሆን ይችላል።ኑሮ ሲከብዳቸዉ ይመስለኛል ተማሪዉ ወይም ት/ት ሊከብደዉ ይችላል ወይም ላይፍ ግቢ ላይ ያለዉ ላይፍ ለምሳሌ ኣንድ ሴት ደምኛ ከተደረገች ተናዳ የምትጀምረዉ ሊሆን ይችላል እንጂ ደስ ብሎት የሚጀምር ሰዉ ይኖራል ብዬ ኣላስብም።

**P 1: 1_Female Users FGD in Busness college.docx - 1:29 [?? 2:-????? ???? ??? ?????? ? ..] (61:61) (Super)**

Codes: [Motivators for substance use- Peer pressure - Family: 3_Motivating factors for substance use]

No memos

ኮድ 2-እንደዚህ ኣይነት ነገር የሚጀምረዉ በ peer pressure ምክንያት ነዉ።

**P 1: 1_Female Users FGD in Busness college.docx - 1:33 [?? 4:- peer pressure ????? ???..] (63:63) (Super)**

Codes: [Motivators for substance use- Peer pressure - Family: 3_Motivating factors for substance use]

No memos

ኮድ 4- peer pressure ላይፋችን እንዳለ ሊኖር ይችላል።ነገር ግን ከካንፓስ ላይ ሃይስኩል ላይ ነዉ ያ ነገር

**P 1: 1_Female Users FGD in Busness college.docx - 1:90 [????? ?? ??? ????? ????? ?????..] (111:111) (Super)**

Codes: [Motivators for substance use- Peer pressure - Family: 3_Motivating factors for substance use]

No memos

በእርግጥ ይሄ ነገር በስልጣኔ የተያያዘ ነዉ።ማጨስ ማቆም መጠጣት የእኛ ባህል ኣየደለም።ስለዚህ ለተማሪ መጥተሽ ስልጣኔ በሚልጋ ኣንድ ወጣት ስላጨሰ ስልጣኔ ነዉ።እንደዛ ነዉ እየታሰበ ያለዉ።ማጨስ ጀብድ ነዉ።እና በኣከባቢዉ መኖር ተፅዕኖ ኣለዉ።

**P 2: 10_M_IDI_Male non user BC.docx - 2:2 [?? ?? ?? ????? ??? ???? ?? ???..] (10:10) (Super)**

Codes: [Motivators for substance use- Peer pressure - Family: 3_Motivating factors for substance use]

No memos

እቲ ሓደ ሓደ መንእሰይ እንሆ በተለይ ካብ ማእከል ሃገር ዝመፅእ ናይ ምዕባለ ወይ ናይ ሰልጣነ ምልክት መግለፂ ገይሩ እዩ ዝወሰዶ እና ይጥቀም እዩ

**P 2: 10_M_IDI_Male non user BC.docx - 2:4 [?? ??? ?????? ???? ???? ?? ???..] (10:10) (Super)**

Codes: [Motivators for substance use- Peer pressure - Family: 3_Motivating factors for substance use]

No memos

ሐዚ ምሳና አዕረክትና ዝነበሩ ንፈልጥ ኢና ከመይ ከም ዝአትው ገለ እቱ እናውሰኸ ከይዱ ዝበልኩኻ እና 1ይ ዓመት እናሃለኩ አዕርክተይ ነይሮም አነ ነይረ እና ዝተወሰኑ ሰባት እዮም ነይሮም ዝጥቀሙ ካብኡ ግን እንታይ እዩ ከምዚ ናይ ሕርፍና መግለፂ ገይሮም ሰለ ዝወሰድዎ እቲ ጫት እቶም ገለገለ አዕርክቲ እንታይ ኮይኖም ምሰኦም ምጥቃም ጀሚሮም

**P 2: 10_M_IDI_Male non user BC.docx - 2:13 [???? ?? ?? ??? ????? ??? ??? ?..] (20:20) (Super)**

Codes: [Motivators for substance use- Peer pressure - Family: 3_Motivating factors for substance use]

No memos

እንታይ እዩ ካብ ዕሽል ከተማታት ዝመጽ ከምዚ ናይ ምዕባለ መግለጺ ገይሩ ስለ ዝሓስቦ /ዝወስዶ ክብለካ ብዙሕ ነገር ኣሎ ዓብዪ ከተማ ከይንካ ትሓስብን ወይ’ውን ወዲ ገጠር ኮይንካ ትሓስቦ ዝኮነ Extreme ትሓስቦ ኣለኻ ናይ ባዕልካ ነገር ግን እቶም ምካከለኛ ዝኾኑ ከምኡ ይገብሩ። ካብኡ ከዓ ክብለካ ናይ ተፅዕኖ ክወድቅ ዝክእል እንተኢልና ንሳቶም ይመስለኒ ከምዚ ስብ ዝበሎም ክኮኑ ሓሪፍ ዝብሃል ወዲ ከተማ ንዑኡ ክመስሉ ስለ ዝደልዩ ናብ ዝበሎም ናይ ምስማዕ ባህሪያት ኣሎ።

**P 2: 10_M_IDI_Male non user BC.docx - 2:33 [?? ??? ?? ?? ???? ??? ?? ???? ..] (47:47) (Super)**

Codes: [Assumed purpose to strat substance use- Relief from anxity - Family: 4_Percieved importance of subatance use by users] [Motivators for substance use- Peer pressure - Family: 3_Motivating factors for substance use]

No memos

እሞ እተን ከም ከም ዓይነቱ እቶም ሱስ ይፈላላ እዩ ዕላመኦም ንአብነት ሲጋራ እንተ ኢልካ ሱቅ ኢልካ ንሙድ ንወዲ ከተማነት መግለፂ እዩ ወይ ከዓ ካብ ቅጥዒ ዝተልዓለ ሓደ ሓደ ሰባት ወይ ተምሃሮ ተበሳጭዩም ክጥቀሙ ይኽእሉ እዮም ንሳ እያ ክትከውን ትክእል፡፡

**P 2: 10_M_IDI_Male non user BC.docx - 2:35 [??? ?? ??? ?? ??? ?? ?? ??? ??..] (47:47) (Super)**

Codes: [Motivators for substance use- Peer pressure - Family: 3_Motivating factors for substance use]

No memos

ካብኡ እቲ መስተ ገለ ዝብል ግን ሐደ ባህሪ ውን አሎ መስተ ናይ ምስታይ ገለ ዋላ ስዋ ዝለመደ ስብ ክኽውን ይኽእል መስተ ዝስቲ ዋላ ካብ ከተማ ዝወፀ ስብ ክኸውኝ ይኽእል ወዲ ሃብታም ክኸውን ይኸጽል መግለፂ ካብ ወልዱ ዝተምሃረ ክኸውን ይኽእል ፅቡቕ መስተ ሰቲኻ ገለ ዝብሉኻ።

**P 2: 10_M_IDI_Male non user BC.docx - 2:55 [Adult ???? ?? ??? ??? ??? ?? ?..] (69:69) (Super)**

Codes: [Motivators for substance use- Peer pressure - Family: 3_Motivating factors for substance use]

No memos

Adult ክበሃል ከሎ ደሓን እዩም እዙይ ግን መንእሰይ እዩ ተደፋፋአይ እዩ Firye age ዝበሃል ነገር እውን እዩ ደሓር እንታይ እዩ ሐዚ ንአብነት ዝኾነ Group ደስ ዝብለካ መንእስይ ስለ ትረክብ ብቀረባ አብ መንድረ እንተኮንካ ገሊኡ ዓብይ ገሊኡ ግዛዕ ክትረክብ ትኽእል ኢኻ ቀሊል አይትረክብን አብዚ ግን መንእስይ ንምንእስይ ኢኻ ትራኸብ Group ናይ ምፍጣር ባህሪ አሎ

**P 2: 10_M_IDI_Male non user BC.docx - 2:56 [??? Group ????? ??? ?? ??? ???..] (69:69) (Super)**

Codes: [Motivators for substance use- Peer pressure - Family: 3_Motivating factors for substance use]

No memos

ከምዚ Group ክትፈጥር ከለኻ ከዓ ብዙሕ ባሕሪ ዘለዎ ሰብ ኢካትረክብ ሓሽሽ ዝጥቀም ሺሸ ዝጥቀም ጫት ሐዚ ሪኢኻ እታ ዓረከይ ብሓደ ሻሂ ንሰተ በሓደ ንጫወት ገለ ዶ ይመፅእ አብ ትርፊ ሰዓት ዳሓር እንታይ ትኸውን አለኻ እቲ ሓደ ናብቲ ናይ ገለ ክወስደካ እየ ክድፋፈአካ እየ ናይ ዓሪኪ በቃ ዓረኪ ዝበሃል የደፋፈእ እዩ በኡ ምኽንያት ሐደ ኩነታት ንሱ እዩ እቲ ግቢ መንእሰይ ብምኻኑ ካብኡ ከዓ ዓርካይ ዓርከት አሎ ብዙሕ ባሕሪ ዘለዎ ሰብ አሎ ዝደፋፈአካ

**P 3: 11_M_IDI_Male nonuser BC.docx - 3:15 [??? ????? ????? ?? ??????] (27:27) (Super)**

Codes: [Motivators for substance use- Peer pressure - Family: 3_Motivating factors for substance use]

No memos

የሆነ አራዳነት መስላቸው ነው የሚጠቀሙት

**P 3: 11_M_IDI_Male nonuser BC.docx - 3:17 [???? ??? /???/ ?? ???? ????? ?..] (29:29) (Super)**

Codes: [Motivators for substance use- Peer pressure - Family: 3_Motivating factors for substance use]

No memos

ትንባሆ ደግሞ /ሲጋራ/ ደም ምንለው ገዳኛችን ሊያጨስ አየነው እኛም እናጨሰለን ብለው እሱ አራዳ ነው እኛም አራዳ ነን ብለው ነው የሚያስቡት

**P 3: 11_M_IDI_Male nonuser BC.docx - 3:18 [??? ???? ??? ??? ?????? ?? ?? ..] (29:29) (Super)**

Codes: [Motivators for substance use- Peer pressure - Family: 3_Motivating factors for substance use]

No memos

ደግም ካላጨሱ ዳግም የሆነ በውስጣቸው ላይ ደሰ አይላቸውም እየለመዱ ሊመጡ እና በዛው ለምደው ይቀራሉ ተረድተሀኛል

**P 3: 11_M_IDI_Male nonuser BC.docx - 3:22 [???????? ???? ?? ???? ???? ?? ..] (31:31) (Super)**

Codes: [Motivators for substance use- Peer pressure - Family: 3_Motivating factors for substance use]

No memos

አጀማምራቸውነ በለቀም ያው በጋደኛ በጋደኛ ነው የሚጀመረው ጫትን የሚጠቀሙት ቅድም እንዳልኩት ለማንበብ ብለው ነው ትንባሆ/ሲጋራን ደግሞ የሚያጨሱት ቅድምም ተናግራዋለሁ ጋደችን አጭሰዋል እኛም አሪፍ ነንን እነሱም አሪፍ ናቸው ብለው ጋደኞችዋ ስላጨሱ ብቻ ነው የሚያጨሱት

**P 3: 11_M_IDI_Male nonuser BC.docx - 3:23 [????? ????? ?? ??? ?? ?? ??? ?..] (33:33) (Super)**

Codes: [Motivators for substance use- Peer pressure - Family: 3_Motivating factors for substance use]

No memos

የአልኮል የሚባለው የሄ ኦቨር ማታ ላይ ጭፈራ ምናምን ነገር አለ እና ከሴቶች ጋር አብሮ መጨፈር እንዳትጨፍር የሆናል መጨፈር ፋሸን ሰለሆነ በዚህ ግቢ ላይፍ ወሰጥ እና ይህንን ለመጨፈር 1 2 3 4 እያሉ ይጠጣሉ በዛው የአልኮል መጠጦች ተጋላጭ ሆነው ይቀራሉ ይሄን ነው ያልኩት

**P 3: 11_M_IDI_Male nonuser BC.docx - 3:31 [?? ??? ??? ????? ???? ??? ??? ..] (41:41) (Super)**

Codes: [Motivators for substance use- Peer pressure - Family: 3_Motivating factors for substance use]

No memos

እኔ ዳግም ከማን አንሳለሁ የሚለው ነገር ከማን እነሳለሁ ጋደኛዮ ይሄን እየተጠቀመ እኔ ለምንድን ነው የማልጠቀመው በሚለው ይሄ ነገር አለ

**P 3: 11_M_IDI_Male nonuser BC.docx - 3:43 [???? ?? ????? ??? ?? ??? ???? ..] (53:53) (Super)**

Codes: [Motivators for substance use- Peer pressure - Family: 3_Motivating factors for substance use]

No memos

አልኮል ላይ አብዛኛው የዚህ ግቢ ተማሪ አልኮል ይጠቀማል ለምን ይው አንደኛ ሪሌሽን ሺፕ/relationship/ ለማግኘት ብለው ነው የሚጠቀሙት ከሴቶች ጋር /Over/ ኦቨር የሚባል ነገር አለ ኦቨር ለመውጣት ብለው ነው ይሄን ነገር ማንኛውም ተማሪ መጠጥ አቁም ብለህ ብትመክረው ዋ እንትናሳ ለምን እየጠራ እንደዚህ ብትለኝስ ይሄ አይጠጣም ብትለኝስ ምናምን ነገር ብለው ነው የሚያስቡት

**P 4: 12_MAle KIR_BC_round .docx - 4:14 [Fresh ????? ????? ??? ??? ?? ?..] (13:13) (Super)**

Codes: [Motivators for substance use- Peer pressure - Family: 3_Motivating factors for substance use]

No memos

Fresh ተማሪዎች ከቤተስብ ሲመጡ ብሮች በዛ ያለ ብር ይዘው ይምጣሉ። ወደ ግቢ ይገባሉ።በዛ ያለ ብር ይዘው ማለት ነው። መቃም ማጨስ እና መጠጥን እንደ ሙድ ከሚይዙት ጊደኞቻቸው ትላልቅ ጎዎደኞቻቸው እዚ ግቢ ካሉት ቀድመው ከገቡት ተማሪዎች ከሚድርስባቸው ተፅእኖኣኦች የተነሳ ለመጀመር ይገደዳሉ

**P 4: 12_MAle KIR_BC_round .docx - 4:18 [???? ?? ????? ????? ??? 07?23 ..] (16:17) (Super)**

Codes: [Motivators for substance use- Peer pressure - Family: 3_Motivating factors for substance use] [Motivators for substance use- Previous exposure - Family: 3_Motivating factors for substance use]

No memos

ቤተስብ ላይ ጀምረወት የነበረው ነገር 07፡23

የጀመሩት ነገር ካለ እዚ ካሉት ቀድም እዚ ጀምረው ከነበሩት ጋር በመቀላቀል እንደ ሙድ ኣርገው ብመያዝ ይቀጥሉበታል። የሚል ንገር ነው የነገረኝ እነዚህ ስለዚ ተጋላጮቹ እነሱ ናቸው ማለት ነው።

**P 4: 12_MAle KIR_BC_round .docx - 4:25 [fresh ?? ????? peer pressure ?..] (30:30) (Super)**

Codes: [Motivators for substance use- Peer pressure - Family: 3_Motivating factors for substance use]

No memos

fresh ላይ እንደመጡ peer pressure ያጋጥማችዋል እና እዚ የነበሩ የነሱ ጓደኞች ለምሳሌ ሲጋራ የሚያጨሰ ከሆነ የመጣው በቃ ተማሪው ሲጋራ የሚያጨሰ ከሆነ በቃ ሙድ አንተ የገጠር ልጅ ነህ ወይ እንደዚህ ምናምን influence ነው፡፡ የሚኖረው በዚህ አይነት ይጀምራሉ ፡፡

**P 4: 12_MAle KIR_BC_round .docx - 4:27 [????? ???????? ??? ??? ????? ?..] (32:32) (Super)**

Codes: [Motivators for substance use- Peer pressure - Family: 3_Motivating factors for substance use]

No memos

አብዘኛው እንደነገርኩህ ቅድም ፈሬሽ ተማሪዎች ሲገቡ More ብዙ ብር ይዘው ይገባሉ፡፡ ብዙ Expectation ስላለው ማለት ነው ከቤተሰብ እና እነሱ ስለሚመጡ እዚህ ያሉት ጓደኞቻቸው የነሱ ብዙ ነገር ይጠብቃሉ፡፡ እና እነሱ ጋ ብዙ ያጠፋሉ ማለት ነው፡፡ እዚ ደሞ ነበር ተማሪዎች ምናምን ካሉ አብዛኛውን ግዜ ጥሬ ብር ይዘው እንደማይመጡ ነው የነገረኝ

**P 4: 12_MAle KIR_BC_round .docx - 4:32 [Peer Pressure ???? ??? ??? ?? ..] (41:41) (Super)**

Codes: [Motivators for substance use- Peer pressure - Family: 3_Motivating factors for substance use]

No memos

Peer Pressure ያልኩህ ነገር ማለት ነው ወይም ደም እዚ ደሞ ሌላ ከርም የመጣ ጓዋደኛ ካለው ስለሱ ባህሪ የሚያውቅ እዛ የጀመረው ነገር አሁን ለምሳሌ ይጠጣ ከነበረ አጠጣም እንዴ ምናምን ሊሉ ይችላሉ፡፡ እና እዚ ይጀምራል ማለት ነው ያዳብረዋል ማለት ነው፡፡

**P 4: 12_MAle KIR_BC_round .docx - 4:44 [??? ??? ???? ??? ?? ????? ????..] (57:57) (Super)**

Codes: [Motivators for substance use- Peer pressure - Family: 3_Motivating factors for substance use] [Process of getting addicted (from intitiation to addiction) - Family: 5_Process of getting addited for substances]

No memos

ያለው ተማሪ ጎዋደኛ ካለህ ዛሬ አንጠጣም ምናምን ይበባላሉ እና ካለው እሱ ወስዶ ያጠጣዋል ይሄዳሉ ማለት ሌላ ግዜ ያኛው ብር ካለው የሆነ እንትን ይስማዋል፡፡ ባለፈው እሱ ጋብዞኝ ነበረና ዛሬ ደሞ እኔ ልጋብዘው አይነት ነገር ማለት ነው ከዚያ ይሄዳሉ እንደዚህ ነገር እንደዚህ እያረጉ Develop ያረጉታል ማለት ነው ሲያረጉት በቃ ሌላ ጎዋደኛ ይሰበስባሉ ማለት ነው አሁን እዚጋ ምን አለ ከተማ በሚወጡበት ሰዓት እነዚ የአልኮል መጠጦች እዚ ግቢ አይገችም ብዮሃለው እና ከተማ ሄደው ነው የሚጠጡት ወይ ደሞ እዚ በቅረቢያ ቅድም በጠቀስኩልህ የመጠጥ ቤቶች አሉ እና ሲሄዱ እንትን አለ

**P 4: 12_MAle KIR_BC_round .docx - 4:45 [??? ??? ?? ????? ?? ?? ?? ????..] (57:57) (Super)**

Codes: [Motivators for substance use- Peer pressure - Family: 3_Motivating factors for substance use]

No memos

ከተማ ሄደው ነው የሚጠጡት ወይ ደሞ እዚ በቅረቢያ ቅድም በጠቀስኩልህ የመጠጥ ቤቶች አሉ እና ሲሄዱ እንትን አለ ምን አለ Local /ሎካሎች በጋንታ የGroup አላቸው እና ወይ 10 ወይ 8 ይሆናሉ እነሱን Confront ለማረግ የነሱን እንትን እዛ ወይ ብእርቸን ይቀሙዋቻል ወይ ደሞ ስልካቸው ይቀሙዋቻል ለዛ ብሎ ከዚ መጠጣት ያልጀመረ ወይ በአካሉ ግብድያ የሆነ ጉዋደኛ ወይ ደሞ በቃ ብዙ ሆነው ይሄዳሉ ከዚ ማለት ነው ፡፡ በሚሄዱበት ሰዓት ያለመደውም More ከዚያ በኃላ እንደለምድ ይገፋፉታል፡፡

**P 4: 12_MAle KIR_BC_round .docx - 4:46 [??? ??? ???? ??? ???? ???? ???..] (57:57) (Super)**

Codes: [Motivators for substance use- Peer pressure - Family: 3_Motivating factors for substance use]

No memos

ከዚህ ሶስት የሚጠጡ ከሆኑ ሎሎችን ለስላሳ ትጠጣለህ ብለው ይዞዋቸው ይሄዳሉ ከዚ ማለት ነው፡፡ እዛ ከሆዱ በኃላ በብዙ ምክንያት ማለት እንደዚህ ለስላሳ ይዘው እየሄዱ በለስላሳው ላይ ሌላው ቢራ ምናምን እያስለመዱዋችዋል ማለት ነው በግቢ ውስጥ አንዱ ተፅእኖ ፈጣሪ ነገር አንዱ ይሄ ነው፡፡

**P 4: 12_MAle KIR_BC_round .docx - 4:59 [???? Relationship ???????? ???..] (87:87) (Super)**

Codes: [Motivators for substance use- Peer pressure - Family: 3_Motivating factors for substance use]

No memos

የነሱን Relationship ስንመለከታቸው ከነሱ አይነት እንትኖች ጋ አሁን አደነዘዥ ጫት የሚጠቀም ተማሪ እና ሌሎች እንደሱ አይነት የሚጠቀም ተማሪ እና ሌሎች እንደሱ አይነት የሚጠቀሙ ጫት ያለ ተማሪ በጣም ምንም Relationship ነው ያላቸው ዛሬ አንደኛው ከሌለው በቃ 50 gram ከሆነ 5 ሆነው መቃም ይችላሉ ሲጋራ ገዝቶ ከሆነው አንዱ 3 ወይ 4 ሆነው ሊጠቀሙት ይችላሉ፡፡ በጣም ነው እንትን የሚሉት የሚተዛዘኑት ማለት ነው፡፡

**P 4: 12_MAle KIR_BC_round .docx - 4:60 [???? ????? ??? ?? ?????? ??? ?..] (87:87) (Super)**

Codes: [Motivators for substance use- Peer pressure - Family: 3_Motivating factors for substance use]

No memos

አልኮል የሚጠጡት በግቢ በቃ እንዳጋንታ እንደ ቡድን አይነት ነገር ይመስርታሉ እንዲያውም ማለት ነው፡፡ እንደ ቡድን እንደቡድን ይመሰርታሉው፡፡ 40፡45 ሲገቡም እንደዛ Relationአቸው ለነሱ ማለት ነው ከጉዋደኛቻቸው ጋ በጣም hard የሆነ Relationship አላቸው፡፡

**P 5: 13_Male KIR_BC_Round III.pdf - 5:15 [?? ?? ?? ????? ???? ?????? ???..] (3:1569-3:1868) (Super)**

Codes: [Motivators for substance use- Peer pressure - Family: 3_Motivating factors for substance use]

No memos

ላሊ ጫት ቤት እንዯዚህ ኣይነት ተማሪዎችን በጣም ይተሳሰባለ የሚቅሙ ተማሪዎች ኣንዴ ተማሪ ካሇው

ይበቃናሌ ላሊ ቀን እኔም ኣይኖረኝም ብል ስሇሚያስብ ምንዴን ነው ያሇኝ ኣንዴ ተማሪ ቤተሶቦቼን ከምጠይቅ እነዚህ ያለት

ጓዯኞቼን ይጠይቁኛሌ ምንሁነህ ነው ብዙነገር ይጠይቁኛሌ እዚህ ቤተሶበቼን የሚያውቁት ነገር የሇም እነሱ ግን በጣም ብዙ

ነገርን ይጠይቁኛሌ እነዚህ ሲጋራ የሚያጨሱ ተማሪዎች ማሇት ነው ኣንዴዋን ሇ3 ሇኣራት እንትን ብሇው እንዯሚጠቀሙ

እንዯሚጠቀሙ ጠይቃሇሁ

**P 6: 14_X_FGD male non-user at Ayder docx.docx - 6:3 [?? ?? ??? ???? ?? ????? ??? ??..] (15:15) (Super)**

Codes: [Motivators for substance use- Peer pressure - Family: 3_Motivating factors for substance use]

No memos

ከዛ ደሞ ኣንድ ኣንዶቹ ደሞ የመጠቀም ሁኔታ እንደ ከተማ ኣራዳነት እንደዛ ኣስቦ የሚያደርጉ ተማሪዎችም ኣሉ በቃ እየጨመረ እንደመጣ ነው

**P 6: 14_X_FGD male non-user at Ayder docx.docx - 6:10 [?? ??? peer infulence ???? ?? ..] (20:20) (Super)**

Codes: [Motivators for substance use- Peer pressure - Family: 3_Motivating factors for substance use] [who use substnces: Non-dorm Vs Dormitory - Family: 6_Who are at risk of practicing subatnce use]

No memos

በዛ የራሱ peer infulence ስላለው ከዛ ኣንድ ተማሪ ሲጀመር ደግሞ ከዛ ውጭ ቤት መከራየት ይጀምራሉ ምናምን ነገር ከዛ እሱደሞ በተለይ እዚህ ግቢ ኮመን ነው ደህናደህና የሆኑ ተከራይተው በጣምውጭ ይጠቀማሉ

**P 6: 14_X_FGD male non-user at Ayder docx.docx - 6:15 [??? ?????? ??? ?? ??? ?? ?????..] (23:23) (Super)**

Codes: [Motivators for substance use- Peer pressure - Family: 3_Motivating factors for substance use] [Motivators for substance use- Previous exposure - Family: 3_Motivating factors for substance use]

No memos

መቀሌ ዩንቨርስቲ በዚህ ግቢ ማለት ነው ኣድንዛዥ እፅና ኣነቃቂ ኣብዛኛው የሚጠቀም ወይ ከድሮ ልምድ የነበረው ነው ወይም እዚህ ከገባ ኣንዱ moderenization ስለሚያስቡ ነው

**P 6: 14_X_FGD male non-user at Ayder docx.docx - 6:28 [??? ????? ?? ?????? ? ??? ??? ..] (39:39) (Super)**

Codes: [Motivators for substance use- Peer pressure - Family: 3_Motivating factors for substance use] [SU_ Trend in tearms of time - Family: 1_Commonly used substances among univeristy students]

No memos

ቦታው ተማሪዎች ብቻ ኣይደለዉም እ ማለት ካንተ በላይ የተማሩ ሰዎች ኣስተማሪዎችም ዶክቶሮችም ጋዎናቸዉ ኣዉጥተው ሚገቡ ዶክቶሮች ኣሉ ይሄ የተደበቀ ነገር ኣይደለም

**P 6: 14_X_FGD male non-user at Ayder docx.docx - 6:29 [?? ?????? ???? ????? ??? ??? ?..] (39:39) (Super)**

Codes: [Motivators for substance use- Peer pressure - Family: 3_Motivating factors for substance use]

No memos

ከኛ ከተማሪዎች ባይሆን ከታመመው የመጡ ሰዎች ሲያወሩ የሰማናቸው ወሬዎች ኣሉ እዚህ ልታከሙ መጥተው በቃ እዚህ ዶክተር ሁኖ ጋወን ይዞ ምናምን መጥቶ ጋወኑ ኣውልቆ እ ወደ ጫት ቤትእየገባ የሚኝ ኣስተማሪም ዶክተርም ኣለእና እነሱ እንከን እንደዚህ ኣድርገው ሲጠቀሙ ተማሪዎች እማ ያው ያን ነገር ቀላል ነው የሚሆነው

**P 6: 14_X_FGD male non-user at Ayder docx.docx - 6:38 [????? ?? ??? ??? ??? ??? ???? ..] (54:54) (Super)**

Codes: [Motivators for substance use- Peer pressure - Family: 3_Motivating factors for substance use] [who use substnces: Fresh Vrs Senior students - Family: 6_Who are at risk of practicing subatnce use]

No memos

ከዛለላይ ደሞ መቸም ካንድ ዶርም ውስጥ ስምንት ኣለ ስድስት ኣለ ከዛ ውስጥ የግድ ኣንድ ሱሰኛ ሊኖር ይችላል ከዛ ውስጥ ጓደኛቹ ይጨምርና ይወስዳቸዋል ና ያው እነሱደሞ ያነቃቃል ሲልዋቸው መስልዋቸው የገባሉ ከዛ ሱኛ ይሆናሉ ከዛ ኣብዛኛው ተማሪ ከሁለተኛናሶስተኛ እንዲ የመጀመርያ ኣመት ተማሪ ብዙ አይጠቀምም ኮንሲደር የሚያደርገው

**P 6: 14_X_FGD male non-user at Ayder docx.docx - 6:46 [???? ??? ??? ??? ????? ????? ?..] (63:63) (Super)**

Codes: [Motivators for substance use- Peer pressure - Family: 3_Motivating factors for substance use]

No memos

ጫትጫት ስታይ የሆነ ሃሳብ ከነበረህ ትገዛለህ እና ለዛም ሁለተኛ ደግሞ በዚ ግቢ ዉስጥ የሚያጭሱ ኣሉ እንደ ሺፌር ቻይናዎች እነዚህ ኣሁን እኛ እመርጀንሲ ስናልፍበዛ ውጭ ሁነው እያጨሱ እናያለን ሰዉ ምን ይላል ለነዚህ ለምን ኣይከለኩልም ግቢ ዉስጥ የሚጨሱ ቡዙ ናቸው በዛ ኣይነት ኣለ

**P 6: 14_X_FGD male non-user at Ayder docx.docx - 6:47 [???? ??? ?? ??? ?????? ???? ??..] (63:63) (Super)**

Codes: [Motivators for substance use- Peer pressure - Family: 3_Motivating factors for substance use]

No memos

ሁለተኛ ደግሞ የኛ ብሎክ ከኣስፓልቱ ታቸኛዉ ስፓልት ኣለች እኛ ዶርም ማለት ነው እዛጋ ኣምስት ወይም ስድስት ጭፍራ ቤት ኣለ እና የእነሱ ድምፅ እስከ ኣስር ሰኣት ኣያስተኛም ሁለተኛ ደግም ያ ሙዚቃ እየሰማ እዚ የሚጨናነቅ ካለ ሰው ለምን ዘና ፈታ ኣልልም ብሎ የሚወጣ ኣይታጣም እህህህህ እና በዚህ ምክንያት ነዉ እንዳም በሴቶች እየጨመረ ነዉ ።እየበዙ ነው አዎ

**P 6: 14_X_FGD male non-user at Ayder docx.docx - 6:56 [??? ????? ??? ?? ??? ?? ??????..] (78:78) (Super)**

Codes: [Motivators for substance use- Peer pressure - Family: 3_Motivating factors for substance use] [who use substnces: from Urban Vs Rural - Family: 6_Who are at risk of practicing subatnce use] [who use substnces: Pocket moeney [high Vs Low] - Family: 6_Who are at risk of practicing subatnce use]

No memos

ሌላው ኣብዘኛው እዚህ ጋር እዚህ ግቢ የሚጠቀሙት ሲጋራ ነው ሲጋራ በጣም ኣይጨካከኑም በናትህ ስጠኝ ቢለው half ግማሽዋ ተጠቅሞ ይሰጠዋል በቃ ኣብዛኛው የከተማልጅ ሲጋራ ነው ጫት ደህና የሆኑ ከኣዲስ ኣባባ የመጡ ወይም ደሞ መጀመርያ ለምዶ የመጡ ነው የሚጠቀሙት

**P 6: 14_X_FGD male non-user at Ayder docx.docx - 6:60 [??? ?? peer infulence ? ?? ?? ..] (81:81) (Super)**

Codes: [Motivators for substance use- Peer pressure - Family: 3_Motivating factors for substance use] [Process of getting addicted (from intitiation to addiction) - Family: 5_Process of getting addited for substances]

No memos

ሌላው ደሞ peer infulence ፕ እሱ ደሞ ኣንድ ከጀመረ ለምሳሌ ሌላኛውም ኣንድ ጓደኛቹ እንትን ካሉ ከእነሱ ኣንዱ የጀመረካለ እነሱም ቀስበቀስ ይጀምራሉ ምናምን ለምሳሌ ፈተና ሲያልቅ ኣብረው ነው over night club የሚጠጡት ከወጡ የዛኔ እባክህ ስጠኝ እኔም ልቅመሰው እንጂ ብቻየን ልክ እንደ moderenization ስለሚያስቡት የዛኔ over የዛሬዋ ሌሊት ምንም ኣታመጣም ኣንዲት ሲጋራ ለውጥ ኣታመጣም ይባላል ሌላቀንም ናይት ክለብ ይወጣል ጣጣ የለውም ቅመሰው የዛኔ አልኮል ደሞ infulence ስለላለው ሲጋራ ኣይደለም ሌላም ምንም ነገር ብትወስድ ምንም ኣይመስልህም ሲነጋ ነው የምታውቀው የምትበሳጨው ያቺ over nignt club በጣም ገራሚ ሴቶችም እንደተባለው እዚህ ግቢ የሚያጨስ ኦልሞስት የለም ማለት ይቻላል ናይት ክለብ ከወጣች ግን almost.

**P 6: 14_X_FGD male non-user at Ayder docx.docx - 6:62 [ver nignt club ?? most common ..] (82:82) (Super)**

Codes: [Assumed purpose to strat substance use- Entertainment - Family: 4_Percieved importance of subatance use by users] [Motivators for substance use- Acadamic related - Family: 3_Motivating factors for substance use] [Motivators for substance use- Peer pressure - Family: 3_Motivating factors for substance use]

No memos

ver nignt club አለ most common ነው ሌላው እንዳልኩት የፈተና ስትረስም አለ ከgradu የተያያዘ ኣለኣይደል እና peer influence ኣለ እዚህ 3 ነገር የዚህ ግቢ ተማሪን affect የሚያደርጉ ነገሮች ና ማቸው

**P 6: 14_X_FGD male non-user at Ayder docx.docx - 6:66 [???? ?? ?? ???? ???? ????? ?? ..] (89:89) (Super)**

Codes: [Motivators for substance use- Peer pressure - Family: 3_Motivating factors for substance use]

No memos

ሁለተኛ ደሞ ያው ሲጀመር በቀልድ የሚጀምሩ ኣሉ ስትሻፍድ ስትቀልድ ከጓደኛች ጋር ምናምን በዛ የሚጀምሩ ኣሉ በተለይ ወጣት ሳለህ በቀልድ ምናምንኣብዛኛው የሲጋራ ሱስ በቀልድ እንደጫወታ ብለህ ምናምን ብለው ሚጀምሩት ይህ ይመስላል

**P 6: 14_X_FGD male non-user at Ayder docx.docx - 6:77 [??? ??? ??? ??? ??? ?????? ???..] (115:115) (Super)**

Codes: [Motivators for substance use- Acadamic related - Family: 3_Motivating factors for substance use] [Motivators for substance use- Peer pressure - Family: 3_Motivating factors for substance use]

No memos

ከግቢ ውስጥ ኣንዱ ሲባል ኣንዱ የትምህርት ጫናና ሁለተኛ ደሞ peer infulence ሁለቱ ነው በኣብዛኛው እንትን የሚለው ከግቢ ውስጥ

**P 6: 14_X_FGD male non-user at Ayder docx.docx - 6:83 [?? ????? ????? ??? ?? ?? compa..] (120:120) (Super)**

Codes: [Motivators for substance use- Peer pressure - Family: 3_Motivating factors for substance use]

No memos

ያው ተብሎዋል ኣብዛኛው ነገር ግን እኔ compare ስታደርገው ከግቢ ውስጥ እና ከግቢ ውጭ ነገር ላይ ያለው ላይ ካለው ኣንድ የሚያደርጋቸው ነገር ኣለ peer pressure ነው peer pressure ሁለቱም common ነው ምክንያቱ ውጭ ያሉም peer pressure ያለ ነው

**P 6: 14_X_FGD male non-user at Ayder docx.docx - 6:84 [?? ??? ?? ??? affect infulence..] (120:120) (Super)**

Codes: [Motivators for substance use- Peer pressure - Family: 3_Motivating factors for substance use]

No memos

ውጭ ያለው ሰው በጣም affect infulence የሚያደርገው un employment ነው ስራኣጥነት ካለ ምንም ሚያደርጉት ከሌለ ማጨስ የሆነ ጠላ ይጠጣሉ ከዛ እንደዛ እያሉ ወደ ኣልኮል ኣብዛኛው ሰው ብታየው ተንከራትቶም የሆነ ነገር 5 ,6 ኣመት ስራ አጥቶ ስራቢያገኙም reward ስለ ሆነ alcoholic drinkኩ ደሞ ማቋረጥ ኣይችሉም ማለት ነው ስለዚ የመጀመርያ የሚለው un employment ነው ብየ ማስበው

**P 6: 14_X_FGD male non-user at Ayder docx.docx - 6:85 [??? ???? unemployment ??? ??? ..] (124:124) (Super)**

Codes: [Consquences of substance use: hoplessness - Family: 7_Experiance of consquences of substance use] [Motivators for substance use- Peer pressure - Family: 3_Motivating factors for substance use]

No memos

ከውጭ ያለውን unemployment የስራ ማጣት እንትን እነሱ በሱ ኣማካኝነት ወደሱሱ ሲገቡ እነሱ በጣም ሱሰኛ ይሆናሉ እዚህ ያሉት ተማሪ ደሞ በትንሽ ነገር ነው የሚል እና እነሱ ስያይ ወጣ ሲል ወደዛሲሄድ እነሱ ደሞ እንትን ያደርጋል ያንንነ ነገር እንደሚጠቀሙ ያውቃል የሆነ ነገር ከ ጭንቀቱ ነገር የሚያወጣ ስለሚመስለው ያንን ነገር እንዲጠቀም ይገፋፋዋል ማለት ነው ያንን ነገር ነው ኣሁን እነሱ በብዛት ካለ ኣሁን ከተማ ውስጥ ላይ ካለ በብዛት ካለ ተማሪ ያንን ነገር የመጠቀም እንትን

**P 6: 14_X_FGD male non-user at Ayder docx.docx - 6:86 [??? ??? ??? ??? ???? ???? ??? ..] (126:126) (Super)**

Codes: [Motivators for substance use- Peer pressure - Family: 3_Motivating factors for substance use]

No memos

ማለት ኣሁን ያለው ሲጋራ በተላይ በተለይ ሲጋራ ነው እየበዛ ያለው ኣሁን ከኣንድ ሰው ሲጋራ የሚያጨስ ሰው ኣንድብር ስጠኝ ብትለው ኣይሰጥህም ሲጋራ ስጠኝ ብትለው ግን ይሰጥሃል ዋጋው የሲጋራ ኣንድ ብር ናት ግን በብር ብትጠይቅ ኣይሰጥህም ለዛ ሱሰኛ የሆነ ለሱስ ይጋብዛል እንደሚመስለኝ ማለት ነው

**P 6: 14_X_FGD male non-user at Ayder docx.docx - 6:88 [??? ????? ???? ?? 7 ??? ?? ???..] (129:129) (Super)**

Codes: [Motivators for substance use- Peer pressure - Family: 3_Motivating factors for substance use]

No memos

ኣሁን እንዳለው ጓደኛየ ኮድ 7 ማለት ነው የኣንኢንፕሎይመንት ኣለው እንዳለው ትንሽ ማብራራት ደፈልጌ ነው እነሱ ልጆች they are smart ናቸው በቃ ማለት social ለሰው ያላቸው ኣቅርቦት በጣም ታች ናቸው እኔይቅርታ ኣድርግልኝ እንጂ በቃ ማለት ብዙ ለመውቀስ ኣይደለም ግን ብዙ የሚያጋጥም ኣለ ያው የዚህ ሰራተኛ እና የነሱ ያላቸው ኣፕሮች ስታያቸው በጣም የሚገርም እነሱ ካላቸው ማለት ነው ለምሳሌ የሆነ ልጅ እዛ እዚህ ትጨናነቅ እና የሆነ ወጣ ስትል እዛ የሆነ ቦታ ስትቀመጥ እነሱ ስራ ያጣ ብዙ ኣለ በሄድክበት ነፍ ነው ሌላ ቦታ ኣይቀመጡም ስለዚ እዛ ብቻህን ብትቀመጥ ራሱ በቃ ወይደሞ ትንሽየ ውሃ ጠላ ለመጠጣት ስትሄድ ኣንድ ያሰብካት ጓደኛ እንሁን ዝም ካልክ ተጫወት እንጂ ጭንቀት ኣይወዱም ታወቃለህ ኣለኣይደል ኣይወዱም በቃ ምክንያቱ ምነው ተጨናነቅክ ሁላችን እኮ እንደዛነን በቃ ያፅናናሃል ታውቃለህ ስለዚ ሳታስበው ትወድደዋለህ በቃ ኣለ በቃ በጣም የሚገርም ስለዚ ያላቸው ኢንተራክሽን ከሰው ጋር በጣም smart ነው በጣም የሚገርም ስለዚ ያላሰብከው ነገር እነሱ ስለምታገኝ

**P 6: 14_X_FGD male non-user at Ayder docx.docx - 6:93 [??? ?? ??? ??? ???????? ??? ??..] (137:137) (Super)**

Codes: [Motivators for substance use- Peer pressure - Family: 3_Motivating factors for substance use]

No memos

እነሱ ደሞ በጣም ኮመን የሚያደርጋቸው ፉቸር ደሞ መተጋገዝ እርስ በርሳቸው 100 ብር ብቻ እያለው ሌላ ብር የለውም 100 ብር ስጠኝ ቢለው ኣውጥቶ ነው የሚሰጠው መጨካከን ምናምን ኣይታወቅም

**P 7: 15_X_IDI_ Male proctor Ayder.docx - 7:1 [?????? ???? ??? ???? ??? ?????..] (7:7) (Super)**

Codes: [Motivators for substance use- Peer pressure - Family: 3_Motivating factors for substance use]

No memos

የአደንዛዥ መጠቀም ሁኔታ በአሁን ሰዓት እንደልምድ እየተወሰደ ነው ያለው፡፡ ልምድ ሲል አንድ በ’dorm’ የሚጠቀም ካለእሱ እንደ ምሁርና ንቁ እንደዛውም የከተማ ልጅ አድርጎ ነው የሚወሰዱትና ሁሉም የ’dorm’ አባላት ወደሱ ዓይነት ባህሪ የመሄድ አዝማሚያ ነው የሚኖረው፤ በቀጥታ እንኳን ተጠቃኒ ባይሆኑ ቆይቶ ግን መግባታቸው የማይቀር ነው፡፡

**P 7: 15_X_IDI_ Male proctor Ayder.docx - 7:32 [???? ??? ?????? ??? ??? ??? ??..] (53:53) (Super)**

Codes: [Motivators for substance use- Peer pressure - Family: 3_Motivating factors for substance use]

No memos

ወንዶች ከድሃ ህብረተሰብ የመጣ ከሆነ ምንም ችግር የለውም፤ ሴቶች ግን እነዛ ከደህና ቤተሰብ የመጡ ሴቶች አላቸው እንደፈለጉ ነው ሁሉ ነገር ያደርጋሉ ሁሉ እንደፈለጉ አሁን ዋና ችግር ምንድነው በአንድ ዶርም አንድ ደህና አለች መካከለኛ ወይም ዝቅተኛ ቤተሰብ የመጣች አለች፤ አሁን ያቺ ከዝቅተኛ ኑሮ የመጣች ልጅ ይከብዳታል፣ ልብስ እንደፈለገችው አይደለም፤ ጌጣጌጥ የላትም ዳዩ እየተጠቀሙ እሷ ግን ምንም ስለሌላት ከነሱ እኩል ለመሆን ደጅ መውጣት ትጀምራለች፤ ደጅ መውጣት ምን ማለት ነው? በሌላ አባባል ደጅ ማደር አንዳንድ ነገር ትጀምራለች፤ በዚህ መጠን ከነሱ ታስተያየዋለች የፈለገችው ትለብሳለች፡፡ ስለዚህ ከዝቅተኛ ሕ/ሰብ የመጡ መውጣት የሚጀምሩ ይመስለኛል፡፡

**P 7: 15_X_IDI_ Male proctor Ayder.docx - 7:33 [????? ?/?? ??? ???? ????? ????..] (53:53) (Super)**

Codes: [Motivators for substance use- Peer pressure - Family: 3_Motivating factors for substance use]

No memos

ከዝቅተኛ ሕ/ሰብ የመጡ መውጣት የሚጀምሩ ይመስለኛል፡፡ በዚህ አንዳንድ ነገር ደጅ ማደር ይጀምራሉ ከደህና ቤተሰብ የመጡ የሚፈልጉት አድርገው ይመጣሉ፡፡ እነዛ (ከዝቅተኛ ቤተሰብ የመጡ) ግን በዛው ያድራሉ፡፡ በዚያው ወደ ሌላ ነገር ይገባሉ የሃብታም ልጆች ሱስም ይቀሰቅሳሉ የድሃ ልጆች ግን ደጅ ማደር ብቻ ነው፡፡

**P 8: 16_FGD ARID_Users .docx - 8:5 [?????? ???? ?? ??? ???? ????? ..] (11:11) (Super)**

Codes: [Motivators for substance use- Peer pressure - Family: 3_Motivating factors for substance use] [Process of getting addicted (from intitiation to addiction) - Family: 5_Process of getting addited for substances]

No memos

እንደቀልድ ከጋደኛ ጋር ባለው ቅርበት ቀሰበቀስ ይጀምርና ወደ ሱስ ደረጃ ያደርሳል

**P 8: 16_FGD ARID_Users .docx - 8:16 [???? ??? ?? ??????? ??? ????? ..] (23:23) (Super)**

Codes: [Motivators for substance use- Peer pressure - Family: 3_Motivating factors for substance use]

No memos

ገደኛም ወሳኝ ነው ከተከተልከው ሱሰኛ ትሆናለህ ካልተከተልከው ግን ምንም አትሆንም አንዳንዱ ሞዋያ ስታየ ነው ይሕንን ነገር የምትከተለው

**P 8: 16_FGD ARID_Users .docx - 8:21 [Code2 ???????? ??? ??? ?????? ..] (32:32) (Super)**

Codes: [Motivators for substance use- Peer pressure - Family: 3_Motivating factors for substance use]

No memos

Code2 የዩኒቨርስቲት ተማሪ ውስጥ እንዳጀምር የግል የጓደኛ ተዕእኖ ነው ወደዚህ የሚየስገባው

**P 8: 16_FGD ARID_Users .docx - 8:22 [Code3 ?????? ?? ?? ????? ???? ..] (33:34) (Super)**

Codes: [Motivators for substance use- Peer pressure - Family: 3_Motivating factors for substance use]

No memos

Code3 ኣስተሳሰብ ነው ወሬ ቢደጋገም ትርጉም የለውም ግን ጓዳኛ ስለምን የሉስኛ ጓዳኛ እኔ ከተኣተልኩኝ እኔም እንዳሱ ሱሰኛ እሁናለሁ ማለት ነው ፡፡

ተከትዮም ከልሄድሉ ግን እሱ ብቻውን ወዳሱሱ ይሄደል እግን ኣልሄድኩም ስለዚህ ሱሰኛ አድሆንም ማለት ነው፡፡

**P 8: 16_FGD ARID_Users .docx - 8:34 [?? ???? ??? ???? ?????? ???? ?..] (67:67) (Super)**

Codes: [Motivators for substance use- Peer pressure - Family: 3_Motivating factors for substance use] [Motivators for substance use- Previous exposure - Family: 3_Motivating factors for substance use] [who use substnces: Pocket moeney [high Vs Low] - Family: 6_Who are at risk of practicing subatnce use]

No memos

ጫት ሲጋራና ሓሺሽ ሁሉንም የሚጠቀመት ከፍተኛ ገቢ ያላቸው ቤተሰብ የመጡ ነቸው ብዬ ነው የማሰበው ፡፡ ከታች ከሃይስኩል ማቃቂያ ለጥነት ብለው ጫትን በተለይ ጠሚጀምሩት እዚ ግ ግን ኣራዳ የሆኑ ስልሚለሚመስላቸው ነው ፡፡

**P 8: 16_FGD ARID_Users .docx - 8:35 [??? ???? ??? ?? ???? ?? ??? ??..] (69:70) (Super)**

Codes: [Motivators for substance use- Peer pressure - Family: 3_Motivating factors for substance use]

No memos

ሲጋራ ከጓዳኛ ግፊት እሱ አረሳዳ ነው እኛም እራዳ እንሁን ብለው ነው ከዛ ለዋደው ይቀራሉ፡፡ የኣልከል መጠጥ

የሚገፋፋቸው over የሚባለውን ነገር ከሴቶች ጋር ኣብረው ይጨፍራሉ፡፡

**P 8: 16_FGD ARID_Users .docx - 8:37 [??? ???? ?? ????? ?? ???????] (74:74) (Super)**

Codes: [Motivators for substance use- Peer pressure - Family: 3_Motivating factors for substance use]

No memos

ሲጋራ ከጓዳኛ ጋር ለመለለስ ሃነ አረፍለመበል

**P 8: 16_FGD ARID_Users .docx - 8:38 [??? ??? ???? ??? ??? ??? ?????..] (75:75) (Super)**

Codes: [Assumed purpose to strat substance use- Entertainment - Family: 4_Percieved importance of subatance use by users] [Motivators for substance use- Peer pressure - Family: 3_Motivating factors for substance use]

No memos

ሐሽሽ የንተ የልሆነ ነገር ነገር የንተ እንደሆነ እስከልመተጥ over 1.2 ፣56 እየተበለ ይህ ይሰፈፈል ከሴቶች ጋር አከብሮ ለመጨመፈር ፍሽን ስለሆነ በግቢ ለያፍ

**P 9: 17_Male User_Adihaki campus.docx - 9:8 [???? ???? ?? ???? ??????] (19:19) (Super)**

Codes: [Motivators for substance use- Peer pressure - Family: 3_Motivating factors for substance use]

No memos

በጓዳኛ ተፅእኖ ነው በብዛት የሚጃምረው

**P10: 18_KIR 1_ 1st interview_Busness campus_mekelle univeristy.doc - 10:38 [??? ? Globalization ???? ?? ??..] (52:52) (Super)**

Codes: [Motivators for substance use- Peer pressure - Family: 3_Motivating factors for substance use]

No memos

*አሁን ከ Globalization አንፃር እኛ ከውጭ አገር አንፃር እኛ ‹‹ፋሮች›› ነን ብለን እናስባለን፡፡ ስለዚህ እነሱ ያደረጉት ሁሉ እንተገብራለን፡፡ እዚህ ጋር መጥተህ ደግሞ የከተማ ልጅ ደግሞ የገጠሩ ተማሪ ላይ ያው ‹‹Act›› ያረጋል፡፡ ያው ምክንያቱ ቀድሜ ነቅቻለህ ነው፡፡ የገጠር ተማሪ ደግሞ ከከተማ ተማሪ ጋር አብሮ መሆን ወደ ሱሱ የመግት ነገር አለ፡፡ ያው አራድነት መስሎ ስለሚይታይ ነው፡፡*

**P10: 18_KIR 1_ 1st interview_Busness campus_mekelle univeristy.doc - 10:40 [??? ?? ???? ???? ???? ?? ?????..] (52:52) (Super)**

Codes: [Motivators for substance use- Peer pressure - Family: 3_Motivating factors for substance use]

No memos

*እናም ይህ ተፅዕኖ አለ፡፡ አራነት ነው ሱሰኝነት ብሎ የሚያስብ አሉ፡፡ ሱስ ውስጥ ስትገባ ደግሞ ትቀናበታለህ ያንን ግን ደግሞ የተሳለልህ ነገር ስላላ እንደ ፍርነትም ትወስደዋለህ የተሳለልህ ነገር ስላለ ስላአወቅከው፡፡*

**P10: 18_KIR 1_ 1st interview_Busness campus_mekelle univeristy.doc - 10:41 [I: ???? ????? ??? ????? ???? ?..] (53:54) (Super)**

Codes: [Motivators for substance use- Peer pressure - Family: 3_Motivating factors for substance use]

No memos

***I: አንዳድ ተማሪዎች ሱሰኛ ተማሪዎች ይበልጥ ተግባቢ ቶሎ የሚገባቸው እና ደፋሮች ናቸው ብሎ የማሰብ ነገርስ አጋጥሞሽ ያውቃል፡፡ ይሄ ይንፀባረቃል ግቢ ውስጥ ?***

*P: አዎ፡፡ እውነት ነው ይንፀባረቃል ግቢ ውስጥ፡፡ እንትን እኮ ነው ነገሮችን ቀላል አድርገህ እንድታየው የሚዲርግህ እኮ አስተሳሰብህ ብቻ አይደለም ከሱስ ጋር ተያይዞ የሚመጡ ነገሮች አሉ፡፡ ብዙ በየአይነቱ ነው የምትሆነው (…..ሣቅ)፡፡ ያው ከመሸ በኋላ ነው ሰው አየኝ አላየኝ አትልም ቀን ቀን አትደገመውም የማታውን እና ግድ የለሽ ትሆናለህ፡፡ ግድየለሽ የሚያደርግህ ደግሞ ሰክረህ የሚታደርገው ነገር አታውቅም አጓጉል ነገሮች ታደርጋለህ ፡፡*

**P10: 18_KIR 1_ 1st interview_Busness campus_mekelle univeristy.doc - 10:42 [????? ??? ????????(??) ??? ???..] (55:55) (Super)**

Codes: [Motivators for substance use- Peer pressure - Family: 3_Motivating factors for substance use]

No memos

*የትኛውም ነገር አታከብደም፡፡(ሣቅ) አሁን ለምሳሌ የሆነ ተማሪ “Cheat” አደረገ ቢትባል ፍሬሽ ተማሩ ‹‹እረ ባክህ›› ብሎ ነው በጣም የሚገረመው፡፡ እንደኛ ሱሰኛ የሆነ ሰው ደግሞ ‹‹ውይ አደረገ/አረገች?›› ብለህ ስቀህ ነው የሚታልፈው፡፡ ብዙ አታካብድም ምክንያቱም ነጌ እኔም አደርገዋለህ ብለህ ታስባለህ፡፡ ማለት ነገሮችን ‹‹ክባድ›› ሰጥተህ አታየውም፡፡ ምክንያቱም ለሌላ ሰው ኦ! ብለህ የተገረምክበት ነገር በራስህ ላይ ደርሶ ታገኘዋለህ(ሣቅ) ፣ ስለዚህ ኦ! ብለ እኮ ነበር መባል ስለመ፣ስለማትፈልግ ዋጥ ታደርገዋለህ፣ ቀላል አድርገህ ታየዋለህ፡፡ ከዛ ይሄ እየተለመደ ይሄድ እና ቀለል ያለ እብደት ነካ ሊያረግህ ይችላል፡፡ እብድ ሁነህ ምናምን ግን አይደለም፡፡ አውቀህ እብድህ መሆን ስለሚያዋጣህ ነው፡፡*

**P10: 18_KIR 1_ 1st interview_Busness campus_mekelle univeristy.doc - 10:43 [??? ??? (??? ?????) ?? ?? ?? ?..] (57:57) (Super)**

Codes: [Motivators for substance use- Peer pressure - Family: 3_Motivating factors for substance use] [who use substnces: Pocket moeney [high Vs Low] - Family: 6_Who are at risk of practicing subatnce use]

No memos

*እዛጋ ደግሞ (ሱሰኛ ያልሆኑት) ቡዙ ጊዜ ከኛ ዳም ተማሪዎች ነበሩ እች ኮ ምንም የሀብታም ልጅ ናት ነበር የሚሉን፡፡ ቡዙ ጊዜ ያለመፈለግ ሳይሆን አልታዳልኩም እንደዚህ ዘና ለማለት አልተፈጠርኩም ነበረ የሚሉት፡፡ ግን የከተማው ልጅ ያደረገው ሁሉ ይስባቸዋል፡፡ ከነሱ ደግሞ ጎልተው የሚወጡት ‹ንጥቆ› የሚባሉት ናቸው፡፡ ከከተማ ልጅ ይልጥ አደገኛ ሁኔታ ይገጥማቸዋል፡፡ ማለት የከተማ ልጅ እንደነሱ አይጎዳም፡፡ የከተማ ልጅ ራሱን ይጠብቃል፡፡*

**P10: 18_KIR 1_ 1st interview_Busness campus_mekelle univeristy.doc - 10:44 [????? ?? ????? ?? ???????? ???..] (57:57) (Super)**

Codes: [Motivators for substance use- Peer pressure - Family: 3_Motivating factors for substance use] [who use substnces: from Urban Vs Rural - Family: 6_Who are at risk of practicing subatnce use]

No memos

*የከተማው ልጅ ያደረገው ሁሉ ይስባቸዋል፡፡ ከነሱ ደግሞ ጎልተው የሚወጡት ‹ንጥቆ› የሚባሉት ናቸው፡፡ ከከተማ ልጅ ይልጥ አደገኛ ሁኔታ ይገጥማቸዋል፡፡ ማለት የከተማ ልጅ እንደነሱ አይጎዳም፡፡ የከተማ ልጅ ራሱን ይጠብቃል፡፡ ‹ለማሻሻል› ይሞክራሉ ግን እንደ የከተማው ልጅ ነገሮችን ቀላል አድርጎ የማየቱ ነገር ቶሎ አይመጣም፡፡ እንደዚህ ታውቃቸዋለህ እኛ ራሱ ‹ለመዘነጥ ሞክራለች› ብለህ ነው የምትገልፃት፡፡ ሱስኛ ሲሆኑ ደግሞ በጣም ይገርማሉ(በማዘን ስሜት)፡፡*

**P10: 18_KIR 1_ 1st interview_Busness campus_mekelle univeristy.doc - 10:47 [?? ?? ??? ???? ???? ??? ??? ??..] (62:62) (Super)**

Codes: [Motivators for substance use- Peer pressure - Family: 3_Motivating factors for substance use]

No memos

*ግን ሱስ ሲባል የዕለት የዕለት ሱሰኛ ደግሞ አለ፡፡ የዕለት ተዕለት ሱሰኛው ለማንም አያስቀናም….(ሣቅ)፡፡ የሚያደርጉት ነገር በሙሉ፣ አለባበሳቸው ፣ ጫማቸው ሁሉንም ነገራች ያስቃል እንጂ አያስቀናም፡፡ ምክንያቱም ሁሉም ወጪዎቹ ለሱስ ስለሚያውሉት ማለት ነው ለለብስ መግዣ፣ መቀየርያ እንኳን አይኖራቸውም፡፡ ከዛ ቀጥሎ ያለው ሱሰኛ ደግሞ ይሄ የከተማው ሱሰኛ ነው፡፡ እነሱ ደግሞ እየበሉ የሚጠጡ አሉ፡፡ እነሱ ናቸው የሚያስቀኑት የሚባሉ፡፡ እነሱ ደግሞ ሱሱ ያን ያህል ሲጎዳቸው አታይም፡፡ ምክንያቱም ግንዛባቸው ይሸፍነዋል፡፡ ገንዘብ የማይሸፍነው የለም፡፡ አንተም ያን ህይወት ላይ ትቀናለህ ማለት ነው፡፡ ሁሉንም ነገር አለባበሳቸው ፣ ስታይላቸው ሁሉም ነገራቸው ይስባል፡፡ ያው አሁን እንኳን ባይሆን ስራ አለም ስትገባ አደረገዋሎህ ትላለህ፡፡ ከግዜ በኋላ ይሳካልሃል ማለት ነው፡፡*

**P12: 2_M_FGD_Male user_BC .docx - 12:12 [??? ????? ??? ?????? ???? ?? ?..] (29:29) (Super)**

Codes: [Motivators for substance use- Peer pressure - Family: 3_Motivating factors for substance use]

No memos

አንተ ጓደኞችህ ናቸው የሚወስኑህ ከሱሰኛ ጋር የምትውል ከሆነ ሊጋብዝህ ይችላል እና ምግብ ደግሞ ምግብ ብቻ ሱስ የሆነ ሰው ጋር ብትውል ላንተ ብሎ አይጠጣም እና ፍላጎት ብለህ እንትን ማለት አትችልም፡፡

**P12: 2_M_FGD_Male user_BC .docx - 12:30 [??? ???? ????? ??? ?? ??? ?? ?..] (51:51) (Super)**

Codes: [Motivators for substance use- Peer pressure - Family: 3_Motivating factors for substance use]

No memos

በዚህ በለነው ጀኔሬሽን ማለት ነው አንድ ሰው አጫጫሱ የሚያምር ከሆነ ያሄ ለጅ አራዳ ነው ይባላል አንድ ሰው ሊቅም አሪፍ አቃቃም የሚቆም ከሆነ ይሄ ልጅ በቃ ላይፋን አይታታል ማለት ነው ይባላል

**P13: 20_KIR 1_ 3rd round interveiew_Business Compus_mekelle Univeristy .doc - 13:15 [??? ?? ?? ????? ???? ????? ???..] (18:18) (Super)**

Codes: [Motivators for substance use- Peer pressure - Family: 3_Motivating factors for substance use]

No memos

*ሌላው ጫት ቤት እንደዚህ ዓይነት የለም፡፡ በጣም እርስ በእርስ ያተሳስባሉ፣ አንዱ ለአንዱ ይደጋገፋሉ፡፡ አንዱ ብር ካለው ገዝተው ይከፋፈላሉ፡፡ ሌላ ግዜ እኔም አይኖረኝም ብለው ስለሚያስቡ ነው መሰለኝ ይረዳዳሉ፡፡ እንዲያው አንዱ ምንድን ነው ያለኝ ቤተሰቦቼ ከሚጠይቁኝ ይልቅ እነዚህ የሱ ጓደኞቼ ናቸው ይበልጥ የሚጠይቁኝ፡፡ ፊቴ ቅይር ብሎ ከሄደ ምን ሁነህ ብለው ይጠይቁኛል፡፡ ቤተሰቦቼ ስለኔ ምንም የሚያውቁት ነገር የለም፡፡ ሲጋራ የሚያጨሱ ራሱ አንዷን ለሁለት ለሦስት እስከ መካፈል ድረስ ነው የሚደርሱት፡፡ እነዚህ ነገሮች በዚህኛው ዙር ተመልክቻለሁ ፡፡*

**P14: 21_KIR 2_ 2nd interview_Busness comapas_Mekelle Univeristy.doc - 14:1 [???? ?????? ???? ??? ????? ???..] (4:4) (Super)**

Codes: [Motivators for substance use- Peer pressure - Family: 3_Motivating factors for substance use]

No memos

በመቐለ ዩኒቨርሲቲ ካምፓስ ዉስጥ ተማሪዎች በኪሱ እንደጠመዱ የሚደረጉ ነገሮች በተለይ ግቢ ዉስጥ ከሉ ምክንያቶች የጋደኛ ተፅዕኖ ዋነኛ እንደሆነ ባለፈዉ አንስቼዉ ነበር፡፡ ተማሪዎች በድብቅ የጠቀሙ ወይም ያልጀመሩ የነበሩ ከሱ ጋር ጥብቅ ቁርንነት ካለቸዉ እዚህ ግቢ ዉስጥ ካሉ P college የዲፓርትመንት የአገራቸዉ ልጆች እንዲሁም ከተለያዩ አካባቢ ከሚመጡ ተማሪዎች ጋር በሚመጡ ተማሪዎች ጋር በሚመደቡበት ማለትም በዶርም ሊመደቡ ይችላሉ በዲፓርትመንት ሊመደቡ ይችላሉ ወይም በሌሎች ምክንያታዊ በሆኑና ባልሆኑ ነገሮች በሚያደርጉት ግንኙነት ጀመሩና በሱሱ የመጠመድ ሃይሉ ከፍተኛ ተወቀዋለህ፡፡

**P14: 21_KIR 2_ 2nd interview_Busness comapas_Mekelle Univeristy.doc - 14:2 [??? ??? ?? Fresh ????? ???? ??..] (4:4) (Super)**

Codes: [Motivators for substance use- Peer pressure - Family: 3_Motivating factors for substance use]

No memos

አሁን እዚህ ላይ Fresh ተማሪዎች በተለይ አዲስ የሚጠመዱ ተማሪዎችን specially አሁን ከአሶሳ የሚመጡ ከዲሬደዋ የሚመጡ ተማሪዎች አሉ ከሃይቅ የሚመጣ ተማሪዎች አሉ ብዙዎች እነሱ የሚመጡ ተማሪዎች በግቢ የተለመደ ነገር Welcome Fresh አለ Welcome Fresh አጠቃላይ ግቢዉ Welcome አንድ ግዜ ይላል ከዛ ባለፈ ተማሪዎች በየዲፓርትመንት ብቻ ሳይሆነወ በሃገራቸዉ አለ ከጎንደር የመጣ ጎንደሬዎች Welcome ይላሉ ከአዲግራት የመጣ አዲግራቶች Welcome ይላሉ እንደዚህ ያለ ነገር አለ እና አሁን ለምሳሌ አሶሳ የመጣ ጋደኛ አለ ከድሪደዋ የመጣ ከሃረርአካሲ ተማሪ ጋደኛ አለ እና እዛ አካባቢ ጫት የማይቀምሰዉ devia በሱ ነዉ እነሱ አካባቢ አሶሳ አሁን በቃ የጫት ምርጥ አገር እንደዛ ነዉ የሚታሰበዉ እዛ አገር ማለት ነዉ፡፡

**P14: 21_KIR 2_ 2nd interview_Busness comapas_Mekelle Univeristy.doc - 14:3 [??? ??? Welcome ???? ?????? ??..] (4:4) (Super)**

Codes: [Motivators for substance use- Peer pressure - Family: 3_Motivating factors for substance use]

No memos

እነሱ እዚህ Welcome ስንጠራ ይሰባሰባሉ ከዛ ከለፈ በዲፓርትመንት አለ አንዲት እንደሚተዋወቁ ማለት ነዉ በዲፓርትመንትም ይተዋወቃሉ፡፡ ሌላ ከዲፓርትመንት በላይ በዶርም ነዉ relationኑ በጣም እየጠነከረ ይመጣል፡፡ እዛ ከሌለ ቦታ የመጡ ተማሪዎች እዛ ተደብቀዉ የሚጠቀሙ አሁን የነገሩኝ ተማሪዎች ብዙ ተማሪዎች ማለት ነዉ ቤተሰብ ጋር ያሉ በግልፅ እንደዚህ አይጠቀሙም አንደኛ ቤተሰብ ይፈራሉ ሁለተኛ ከኢኮኖሚያቸዉ ጋር ቤተሰብ አይፈቀድላቸዉም ያንን በመፍራት እዚህ ያንን ነገሩ በቃ ግልፅ ሆነዉ ከነዛ ቦታ ከመጡ ተማሪዎች ጋር ሲቀላቀሉ በነፃነት እንዲጠቀሙ ይገፋፋቸዋል፡፡

**P16: 23_KIR 2_1st interveiw_Busness campus Mekelle Univeristy.doc - 16:11 [Code 5 ???? ???? ??? ?? ??????..] (125:125) (Super)**

Codes: [Motivators for substance use- Peer pressure - Family: 3_Motivating factors for substance use]

No memos

Code 5 የትኛው የተማሪ ክፍል ነው የሚሳተፈው በዚህኛው በቀድሙም ሀሰብ ለመስጠት እፈልጋለሁ አንዳኛ ደህነት በተባለው ነገር ላይ እኔ አልሰማማበትም ምክንያቱም ሱስኛ ሆኖ በደምብ የማስራ ሰው ኣለ ዉሎም ነው ጓዳኛህ ነው የሚወስነው ምን የሚባል አበበል ኣለ ጓዳኛህን ንገረኝና ማንነትህን እናግረሃለሁ የሚባል ነገር ኣለ እና ለሱስ እምትገቢበት ነገር አንዳና አንዳኛ ሁነሽ ምንም ሱስ የልለበት ልጅ ቢዳር አንቺ አይቶ የሚመጠለው ካለ ራሱ መጥቶ የሚጠይቀሽ ሰው ኣለ እዚግቢ ውስጥ ደም ወጥተህ ኦቨር/over/ብትገባ አይቶ ይህ ነገር ዳስ ይላል ማለት ነው ይለል

**P16: 23_KIR 2_1st interveiw_Busness campus Mekelle Univeristy.doc - 16:19 [?? ???? ?? ??? ????? ??? ???? ..] (130:130) (Super)**

Codes: [Motivators for substance use- Peer pressure - Family: 3_Motivating factors for substance use]

No memos

እኛ ለንፐፍ በቃ ግድን አንተኛም ግድን ነፍተን መግበት አለብ እንለለን እ አሁን ነፍቼ ደርም ስገባ የሌለ ሙድ ውስጥ ሆኜ ምንምና ስተኛ ሌለኛው እድ የማይጠቀመው ስውዬ ሲያየኝ ምንሁም ነው አንደዚህ ፊታ የሚከው ሁሌ የለኛል ከዘ የረግኩትን ነገር ኣይቶ ቀለ በቀለ እየየው ሊገበ የችለል

**P17: 24_KIR 3_ Ayder campaus Mekelle Univeristy.docx - 17:5 [?? ??? ???? ??? ???? ?? ?? ?? ..] (22:22) (Super)**

Codes: [Motivators for substance use- Peer pressure - Family: 3_Motivating factors for substance use]

No memos

እወ ኩሎም ተምሃሮ እዮም ኣቀዲሙ ንሱ ናብ ግቢ ኣቲዩ ኣሎ ኩነታት ዝተወሰነ ፈሊጡ ኣሎ ግን ኣብ ሓደ ከባቢ ይነበሩ ስለዝነበሩ ኣበዚ መፅዮም ምሰተራከቡ ብፍቀሪ በሓደ ኣለዉ ድሕሪ ግዜ ንሱ ተጠቃሚ ስለዝነበረ ምስኡ ድማ ብዙሕ ግዜ ንሱ ተጠቃሚ ስለዝነበ ምስኡ ድማ ብዙሕ ግዜ ብተደጋጋሚ ስለትነብር ብፍቅሪ ምክንያት ማለት ማለት እዩ ነሳ ውን ተጠቃሚት ክትከውን ክኢላ ማለት እዩ፡፡

**P17: 24_KIR 3_ Ayder campaus Mekelle Univeristy.docx - 17:21 [?? ???? ???? ???? ????? ?? ?? ..] (49:49) (Super)**

Codes: [Motivators for substance use- Peer pressure - Family: 3_Motivating factors for substance use]

No memos

እወ እንታይ መሲልካ በተለይ እንድሕር ደአ ኣብ ትምህርቲ ዘለዎም ድሌት እንተደኣ ቀኒሱ እቲ ድሌቶም እንተደኣ እናቀነሰ መፂኡ ስለዚ እቲ more ዝውዕሉሉ ኩነታት ወይካዓ ዝሰርሕዎ ስራሕቲ ናብቲ ደገ እዩ፡፡ስለዚ ምስቲ ኣብ ደገ ዘሎ ተጠቃሚ ክከውን ይክእል ወይ ካዓ ምስቲ ኣብ ደገ ዘሎ መንእሰይ ርክቦም እናጠንከረ ከይድ ደሓር ምስቲ ሐደ ሰብ ተፋለጥና ይብሉ ምስ ብዙሕ ሰብ ትፋለጥ ካብ ብዙሕ ደሓር ባህሪ ዘለዎ ሰብ ይረክቡ፡፡ ካብኡ ደሓር ነቲ ናይ ሱስኝነት ተጠቃሚነት እውን ብዝበለፀ ንክጥቀሙ ዝገብርዎም ኩነታት ውን ይረክቡ፡፡ እዙይ ብምንታይ እዩ ዝመፅእ ምስቲ ኣብ ደገ ዘሎ ሰብ relation ወይ ካዓ ዕርክነት ስለትፈጥር ምሰኡ approach ስለትገብር በቃ ካብ ግዜ ናብ ግዜ ዘለካ ርክብ እናወሳካ ብዝከዶ ቁፅሪ ካብ ግዜ ናብ ግዜ ሓቢሮም ዝሰርሕዎም ስራሽቲ ኣብቲ ሱስ ሓቢርካ ምጥቃም ድዩ ሓቢርካ ምስታይ ድዩ ሓቢርካ ምጫስ ድዩ ካብ ግዜ ናብ ግዜ እናወሰከ ይከድ ማለት እዩ ፡፡

**P18: 3_M_FGD_Male User's _Ayder.docx - 18:6 [?? ?? ??? ?? ?? ?????? ??? ???..] (18:18) (Super)**

Codes: [Assumed purpose to strat substance use- Concentration - Family: 4_Percieved importance of subatance use by users] [Motivators for substance use- Peer pressure - Family: 3_Motivating factors for substance use] [Process of getting addicted (from intitiation to addiction) - Family: 5_Process of getting addited for substances]

No memos

ያው እቲ ዘረባ ሓደ እዩ ልመፅናዕቲ ኢልካ ትጅምሮ ካብኡ ፈተና አለኒ ኢልካ ትቅሕም ከምዚ አለና ትቅሕም ቡኡ ቡኡ ሱስ ይሕዘካ በቃ ትጥቀም ትሽወድ ምስ ሓደ ዓርከይ ዓርከይ ማለት እዩ

**P18: 3_M_FGD_Male User's _Ayder.docx - 18:17 [?? ?????? ??? ???? ??? ?? ????..] (43:43) (Super)**

Codes: [Assumed purpose to strat substance use- Concentration - Family: 4_Percieved importance of subatance use by users] [Motivators for substance use- Peer pressure - Family: 3_Motivating factors for substance use] [Relapse: why is happenning_ Relief anxity - Family: 8_Intention to cease and experiance of relapse for substance use]

No memos

ጫት ብመፅናዕቲ ኢልኻ ትኣትዎ ሺጋራ ግን ብዝተፈላለየ ናይ ቤተሰብ ብስጭት ወይ ብናይ በዕሉ ብስጭት ልኾነ ነገር ሓሲቡ ነይሩ እንድሕ ለይተሳኸዐሉ ብኡ ካውኡ እዚአ ኢሉ ምስ ጀመራ ሮብዕ ሓሙስ ጀምዓት ምስ ስሓበላ ብኡ ይኽተብ ማለት እዩ ሰሓቢ ይኸውን ጫት ውን ኸምኡ መስተ ውን ኸምኡ ዓርክኻ እዩ ዘጥፋአካ አነ አይቅሕምን አይትሰን ገለ ኢልካ እንድሕር አእሚንካኒስ በቃ እሱካ ከምኡ እናበለኒስ ፅባሕ ንጉሆ ንወለደይ ድዩ ከሸግር ኢለስ ዝኾነ ነገር ክአስር ይኽእል እዩ ንስተ ተይበልካኒ ግን በቃ ኽልቴናያ ሐደ ኢና ንሓሰብ

**P19: 4_IDI_ Male user BC.docx - 19:20 [??? ??? ?? ??? ??? ??? society..] (57:57) (Super)**

Codes: [Motivators for substance use- Peer pressure - Family: 3_Motivating factors for substance use]

No memos

ከቤት ቅርብ ቦታ መጥቶ እዚህ ሊያይ society ራሲ ግቢ ዩኒቨርሲቲው ራሱ የሚበረታታ ነው Because እዛ ውስጥ የሚቅሙ፣ የሚያጨሱ፣ የሚጨፈሩ ልጆች አሉ እነዛ በማየት ወደዛ የሚያስገባ Source እሱ ነው፡፡ አዲስ ህይወት ነው የምትጀምረው፡፡

**P20: 5_IDI_Male user-BC.docx - 20:9 [????? ??? ???? ???? ????? ?? ?..] (28:28) (Super)**

Codes: [Motivators for substance use- Peer pressure - Family: 3_Motivating factors for substance use]

No memos

አብዛኞቹ ደግሞ እዚህም ገብተው የሚጀምሩ አሉ በጋደኛ ተፅእኖ ማለት ምንም ገደድአት እንደሌለው ጋደኛው ካሳመነው አንድ ሁለት ሦስተ ቀን እምቢ ቢል አራተኛ ቀን ላይ ሊከብደው ይችላል

**P22: 7_F_IDI with Female non user_BC.docx - 22:11 [???? ?? (More )???? ?? ?? ??? ..] (21:21) (Super)**

Codes: [Motivators for substance use- Peer pressure - Family: 3_Motivating factors for substance use]

No memos

በዛላይ ምር (More )ጓደኛሽ ነዉ ግቢ ዉስጥ የሚትመርጪዉ ጓደኛ መምረጥ ኣለብሽ ኣንዱ ጓደኛሽ ወደ ቤተክርስትያን church የሚወስድሽ ከሆነ ኣንዱ ጓደኛሽ ወደ ሱስ ቤት ነዉ የሚወስድሽ ኣንዱ ጓደኛሽ መጠጥ ቤት ነዉ የሚወስድሽ ኣንዱ ጓደኛሽ libraryh ነዉ የሚወስድሽ ስለዚ ላይፎችሽ በኣማራጮች የተመሉ ስለሆኑ ኣንቺ ኣንዱን መምረጥ ነዉ እንደዛ ነዉ ማስበዉ ።

**P22: 7_F_IDI with Female non user_BC.docx - 22:12 [??? ??? ??? ?? ?? ?? ???????? ..] (23:23) (Super)**

Codes: [Motivators for substance use- Peer pressure - Family: 3_Motivating factors for substance use]

No memos

ኣንድ ኣንድ ልጆች ብዙ ግዜ ጫት እንደነገርኩሽ ጫት ለማንበብ ለእንቅልፍ ነዉ ብለዉ ጀምረዉት ይገባሉ ከዛ ደግሞ በምርቃና ሳኣት ማጨስ ይፈልለጋሉ በእንደዛ ነዉ የሚጀሙርት ብየ ነዉ ማስበዉ ወይም ደግሞ ጓደኛሽ እስኪ ምከሪዉ የኔንን ቅመሽ ይሄንን ኣድርጊ እሚልሽን ከሰማሽ ኣንድ ጓደኛሽ እኩል ለመሆን ለመመሳሰል ኣንቺ ተቀምጠሽ እሱ እያጨሰ እየቃመ ደስ ስለማይላቸዉ እንድትጀምሪዉ ስለሚፈልጉ taste ኣድርጊዉ ይሉሻል እንዴ ትጀምሪዋለሽ ደግሞ ሞክሪዉ ይሉሻል ራስሽ በኃላ ሌላ ሰዉ ኣላማማጅ ትሆኛልሽ ከዛ በኃላ በእንደዚ ነዉ የሚጀሙርት ።

______________________________________________________________________

**Code: Motivators for substance use- Poor regulation enforcemetnof in University {2-0}**

**P17: 24_KIR 3_ Ayder campaus Mekelle Univeristy.docx - 17:26 [??? ??? ??? ??? ?? ?????? ?? ?..] (59:59) (Super)**

Codes: [Motivators for substance use- Poor regulation enforcemetnof in University - Family: 3_Motivating factors for substance use]

No memos

ሓዱሽ ነገር ዝብሎ ነገር ምሰ ተጠቃምነት ኣብ ግቢ ተተሓሒዙ ምስ ሕግን ደንብን ናይቲ ግቢ ተተሓሒዙ ዘሎ ድክመት ሓደ ጡበን ደቂ ተባዕትዮ እንተኮይኖም ኣብ ባንቶም /ጁባ ዘለዎ ባንቲ ገይሮም ሲጋራ፣ ጫት ሒዞም ይኣትዉ እዮም ዋላ ሓሺሽ ውን ሒዞም ይኣትዉ እዮም፡፡ ካብኡ እዙይ ሒዞም ረኪብናዮም ክንብል ከለና ናብ ዝምልክቶ ክነሕልፎም ከለና ብቀሊሉ መጠንቀቅታ ብካልእ ብካል ሱቅ ኢሎም ዝሰድዎም ካብኡ ንዳሓር በቃ እዙይ ምንም ማለት ኣይኮነን በቃ ነቲ ኣጋር ዘለዎም ኣረኣእያ ብቀሊሉ ሒዝና ክንኣቱ ንክእል ኢና እንተተረኪብና እውን ምንም ማለት ኣይኮነን ዝብል ኣተሓሳስባ የዕብዩ እና በቃ እቲ ዝወሃብ ቅፅዓት ወይ ከዓ እቲ ሕግን ደንብን ዝከረረ ስለዝኮነ

**P17: 24_KIR 3_ Ayder campaus Mekelle Univeristy.docx - 17:28 [????? ??? ??? ????? ??? ????? ..] (59:59) (Super)**

Codes: [Motivators for substance use- Poor regulation enforcemetnof in University - Family: 3_Motivating factors for substance use]

No memos

ምክንያቱ እዙይ ጥራሕ ዘይኮነስ ብበሪ ተዘይኮነ ውን ካብቲ ዘውግዖ ዘሎ ማለት እዩ ኣበዚ ከባቢ ፅናሕ እሞኒ ናብቲ ቦታ ንስካ ወርውራ እየ በደገ ኮይኖም ናብታ ከባቢ ንሳ ይውርውርዋ ዳሓር ፈቲሾም ካብቲ ከባቢ ይወስድዋ ማለት እዩ ብበሪ ስለዝኣቱ ዝወፅእ ውን ክቆፃፀርዎ ኣይክእልን

______________________________________________________________________

**Code: Motivators for substance use- Poor social life {1-0}**

**P 4: 12_MAle KIR_BC_round .docx - 4:47 [???? ?? ??? ??? ???? ???????? ..] (58:58) (Super)**

Codes: [Assumed purpose to strat substance use- Relief from anxity - Family: 4_Percieved importance of subatance use by users] [Motivators for substance use- Poor social life - Family: 3_Motivating factors for substance use]

No memos

ሁለተኛ ጥል ጥሊቻ ማለት ነው፡፡ ከዶርምተሪዎች ጋ ሊሆን ይችላል ወይ ደሞ ከሌላው ተማሪ ጋ ጥል ካለ ከጥሉ ለመውጣት በቃ የጥሉ ያሳደረውን Pressure ያሃነነ ነገር ለመርሣት ወይ ደሞ ከዛ ነገር ለመውጣት በቃ በንዴት ጠጥተው ምናምን ጠጥቶ ከመጣ በኃላ ወይ ይወጣለታል ያሳደባል ወይ ይማታል ብሎ ያስባል ወይ ካሎን ደሞ በዛ ይረሳዋል በማሰብ እነዚህ ነገሮች ይሄ አንዱ የሚገፋፉ ነገር ነው ማለት ነው፡

______________________________________________________________________

**Code: Motivators for substance use- Previous exposure {29-0}**

**P 2: 10_M_IDI_Male non user BC.docx - 2:6 [??? ?? ?? ???? ???? ?? ????? ?..] (10:10) (Super)**

Codes: [Motivators for substance use- Previous exposure - Family: 3_Motivating factors for substance use]

No memos

ካብኡ ከዓ ያው ብከምኡ ብከምኡ እቲ ተምሃራይ ውን እና መፀ ሓዱሸ ተምሃራይ ይመፅእ ከምኡ ኢሉ አብ ማእከል ሃገር ወይ ከዓ ካልእ ከተማታት ለሚድዎ ዝመፀ ተምሃራይ እናመፀ ክከይድ ከሎ እናበርከተ ይኸድ::

**P 4: 12_MAle KIR_BC_round .docx - 4:17 [????? ?????? ????? ??? ??? ???..] (16:16) (Super)**

Codes: [Motivators for substance use- Previous exposure - Family: 3_Motivating factors for substance use]

No memos

ተማሪዎች የሚመጡበት ኣከባቢና ከመጡ በኃላ የቤተስብ ቁጥጥር ኣለመኖሩ ለፍሬሽነት ያኔ ቤተስብ ላይ ጀምረውት የነበረ ነገር እዛ የነበረ ነገር ካለ እና ያንን ነገር ቤተስብ ላይ ጀምረወት የነበረው ነገር

**P 4: 12_MAle KIR_BC_round .docx - 4:18 [???? ?? ????? ????? ??? 07?23 ..] (16:17) (Super)**

Codes: [Motivators for substance use- Peer pressure - Family: 3_Motivating factors for substance use] [Motivators for substance use- Previous exposure - Family: 3_Motivating factors for substance use]

No memos

ቤተስብ ላይ ጀምረወት የነበረው ነገር 07፡23

የጀመሩት ነገር ካለ እዚ ካሉት ቀድም እዚ ጀምረው ከነበሩት ጋር በመቀላቀል እንደ ሙድ ኣርገው ብመያዝ ይቀጥሉበታል። የሚል ንገር ነው የነገረኝ እነዚህ ስለዚ ተጋላጮቹ እነሱ ናቸው ማለት ነው።

**P 4: 12_MAle KIR_BC_round .docx - 4:26 [??? ??????? ????? ?? ??? ??? ?..] (30:30) (Super)**

Codes: [Motivators for substance use- Previous exposure - Family: 3_Motivating factors for substance use]

No memos

እንደ ጉዋዳኞቻቸው ይወስናል ወይ ደግሞ ከዚያ ይዘውት እንደመጡት እነደጀመሩት ሱስ ይወሰናል ይሄ ነገር ማለት ነው፡፡ አሁን እዛ የሚጠጣ ከነበረ ፍሬሽ ተማሪ እዚ መጥቶ መጀመሪያ ለማጨስ አይደለም እንትን የሚለው ሚመጣ ጓደኛ ፈልጎ ለመጠጣት ነው እንትን የሚለው

**P 4: 12_MAle KIR_BC_round .docx - 4:29 [??? ?????? Experience ????? ??..] (36:36) (Super)**

Codes: [Motivators for substance use- Previous exposure - Family: 3_Motivating factors for substance use]

No memos

በፊት ከነበራቸው Experience ተገስተው ነው እዚ ከመጡ በኃላ የሚጀምራቸው ማለት ነው፡፡

**P 4: 12_MAle KIR_BC_round .docx - 4:30 [?? ??? ??? ???? ???? ????? ???..] (38:39) (Super)**

Codes: [Motivators for substance use- Previous exposure - Family: 3_Motivating factors for substance use]

No memos

ደሞ እዚያ ልምዱ ካላቸው ፍላጎት ኖሮዋቸው በቤተሰብ ቁጥጥር ሊኖር ይችላል ወይም ደሞ በቃ 14፡58

ስለማይፈቀድላቸው ለዛ ሊሆን ይችላል፡፡ እና ከዚ ምክንያት እዛ ፍላጎት ኖሩዋቸው ያልጀመሩት ወይም ደሞ ጀምረውትም More Developed ያላደረጉት ሱስ ካለ ማለት ነው፡፡

**P 6: 14_X_FGD male non-user at Ayder docx.docx - 6:2 [?? main factor ???? ???? ??? ?..] (15:15) (Super)**

Codes: [Motivators for substance use- Previous exposure - Family: 3_Motivating factors for substance use]

No memos

እና main factor ሊኖረው ይችላል ማለት ኣንዳድዶቹ ስንጠይቅ የኣከባቢ ሁኔታ የመጡበትሁኔታ ኣከባቢው እንደዛ ኣይነት ነገር ያለው ከዛ እነሱ ከዛ ሲመጡ ያው ከዛ በዚህ ደሞ ጓደኛ ሲያፈሩ ወደዛ የመውሰድ ኣቅማቸው ያው ስላላቸው ያንን ነገር እንደዛ ስላላቸው ለማድረግ የሚቀል ነገር ነው በዛ ኣይነት ነገር ነው እየጨመረ የመጣው

**P 6: 14_X_FGD male non-user at Ayder docx.docx - 6:15 [??? ?????? ??? ?? ??? ?? ?????..] (23:23) (Super)**

Codes: [Motivators for substance use- Peer pressure - Family: 3_Motivating factors for substance use] [Motivators for substance use- Previous exposure - Family: 3_Motivating factors for substance use]

No memos

መቀሌ ዩንቨርስቲ በዚህ ግቢ ማለት ነው ኣድንዛዥ እፅና ኣነቃቂ ኣብዛኛው የሚጠቀም ወይ ከድሮ ልምድ የነበረው ነው ወይም እዚህ ከገባ ኣንዱ moderenization ስለሚያስቡ ነው

**P 6: 14_X_FGD male non-user at Ayder docx.docx - 6:41 [???? ????? ???? ?? ??????? ???..] (58:58) (Super)**

Codes: [Motivators for substance use- Previous exposure - Family: 3_Motivating factors for substance use]

No memos

ከነዚህ ኣነቃቂና ኣደዛዥ እፅ ብዙሚጋለጡት ተማሪዎቹ ኣንድ የመጡበት ኣከባቢ ይወርስናል ኣሁን ለምሳሌ በዛ ሃረሬ የሚባል ኣከባቢ ከሆነ የመመማው ኣብዛኛዉ የተለመደ ነዉ ጫት ምናምን መጠቀም ።ከዛ ሁሉ እዛ የሚኖር ሰዉ እሱን ሊለምድ ይችላል ።ከዛ እዚ ከመጣ ለምደዋል የግድ እዚሁም መጠቀሙ ኣይቀርም

**P 6: 14_X_FGD male non-user at Ayder docx.docx - 6:42 [?? ???? ??? ?? ?? ??? pre ??? ..] (58:58) (Super)**

Codes: [Motivators for substance use- Previous exposure - Family: 3_Motivating factors for substance use]

No memos

ከዛ ሌላኛዉ ደግሞ እዛ ታች እያለ pre እያለ ምናምን እያለ የሚያቅ ኣሁን እዛ ሲገባ በቃ እንዳለ

**P 6: 14_X_FGD male non-user at Ayder docx.docx - 6:51 [?? ?????? ???? ?? ???? ???? fa..] (73:73) (Super)**

Codes: [Motivators for substance use- Previous exposure - Family: 3_Motivating factors for substance use]

No memos

ያው ተገልፀዋል በፊትም ግን ጥያቄው ከተነሳ factor ነው ነው በርግጥ ከገጠር የመጣ እና ከከተማ የመጣ ሰው ይለያያል ያው ለምሳሌ ሃረር አከባቢ ከሆነ እንግዳ ሁነህ ስትሄድ የሚሰጠው ጫት እንደ በቃ ዘውድ ነገር እህህህ የተለመደ ነገር ነው ይባላል እና ከዛ የመጣ ሰው ኣሁን ምንም ኣይመስለውም ከገጠር የመጣ ግን ላያውቀውም ይችላል

**P 8: 16_FGD ARID_Users .docx - 8:34 [?? ???? ??? ???? ?????? ???? ?..] (67:67) (Super)**

Codes: [Motivators for substance use- Peer pressure - Family: 3_Motivating factors for substance use] [Motivators for substance use- Previous exposure - Family: 3_Motivating factors for substance use] [who use substnces: Pocket moeney [high Vs Low] - Family: 6_Who are at risk of practicing subatnce use]

No memos

ጫት ሲጋራና ሓሺሽ ሁሉንም የሚጠቀመት ከፍተኛ ገቢ ያላቸው ቤተሰብ የመጡ ነቸው ብዬ ነው የማሰበው ፡፡ ከታች ከሃይስኩል ማቃቂያ ለጥነት ብለው ጫትን በተለይ ጠሚጀምሩት እዚ ግ ግን ኣራዳ የሆኑ ስልሚለሚመስላቸው ነው ፡፡

**P 9: 17_Male User_Adihaki campus.docx - 9:7 [????? ??? 11?12? ??? ???? ??] (19:19) (Super)**

Codes: [Motivators for substance use- Previous exposure - Family: 3_Motivating factors for substance use]

No memos

አብዛኛው ተማሪ 11፣12ኛ ጀምሮ የሚመጠ አለ

**P11: 19_KIR 1_ 2nd Ineterbview_ busness compus_Mekelle University.docx - 11:3 [??? ??? ???? ? ?? ???? ???? ??..] (4:4) (Super)**

Codes: [Assumed purpose to strat substance use- Concentration - Family: 4_Percieved importance of subatance use by users] [Motivators for substance use- Previous exposure - Family: 3_Motivating factors for substance use]

No memos

እጫት ደግሞ ከንባብ ጋ ነው በብዛት ሚያዘው ግን እንደዛም ሆኖ ከንባብ ያልተያዘ መንገድ ደግሞ ያለዉ ደግሞ ያዉ ከቤታቸው ይዞት ሚመጡት ነው ማለት ነው

**P12: 2_M_FGD_Male user_BC .docx - 12:2 [?? ??? ?? ???? ?? ???? ???? ??..] (7:7) (Super)**

Codes: [Motivators for substance use- Previous exposure - Family: 3_Motivating factors for substance use]

No memos

ግቢ ውስጥ ብዙ ተጠቃሚ አለ እንደዚ ዓይነት ነገር ግማሽ አይሆንም ቢያንስ 45% በደዚ ዓይነት ነገር ውስጥ ያለው ነው ግማሹ ነፃነት ይከተባል ግማሹ ደግሞ ራሱ ተበላኽቶ የመጣ ነው በቃ

**P12: 2_M_FGD_Male user_BC .docx - 12:3 [??? ???? ???? ?? ???? ?? ?????..] (9:9) (Super)**

Codes: [Motivators for substance use- Previous exposure - Family: 3_Motivating factors for substance use]

No memos

እኔም እንደዛ ይህንን ነው የሚለው እፅ አደንዛዥ ተጠቃሚ 50% ይሆናል 50% ደግሞ በፍላጎት ነው የሚገባው ብየ ነው የሚያስበው

**P12: 2_M_FGD_Male user_BC .docx - 12:6 [?????? ??? ???? ???? ???? ??? ..] (17:17) (Super)**

Codes: [Motivators for substance use- Previous exposure - Family: 3_Motivating factors for substance use]

No memos

ኢንተረስት ስትሉ ለምሳሌ እያየሽ ያደግሹ ነገር ሊሆን ይችላል አሁን ለምሳሌ ከበፊትም ጀምሮ ቤተሰቦቻቸው ወይ ሲጠጣ አይተሽ ታውቅያለሽ ተሰብሰቦ ሲጠጡ የማሕበር እለትም የሆነ በዓል ቢሆን ተሰብስቦ ሲጠጥ ታያለ እና ይህንን ነገር እማ ይሆኑ እኔም አጋጥሞኝ አርጌዋለሁ ብለህ ልታስበ ትችላለህ ሰፈሪህም ይወስነዋል ያደግክበት ቦታ ማለት ነው አሁን መጠጥ ሰፈር ውስጥ ካደክ ይህ የመጠጥ አይነት እንትን ይይዘሃል እድሜህ አይደርስም ስለምትባል ትንሽ እዛ ኤጅ// ላይ በምድር ስበት ታይም እኔም አረጌዋለው ምናምን ብለህ ታስባለህ ከዛ አንዴ ትገባበታለ ላትወጣም ትችላላህ

**P12: 2_M_FGD_Male user_BC .docx - 12:7 [?? ??? ?? ???? ????? ??? ???? ..] (17:17) (Super)**

Codes: [Motivators for substance use- Previous exposure - Family: 3_Motivating factors for substance use]

No memos

ሌላ ደግሞ ጫት ምናምን የምትለው አህን ለምሳሌ እኔ አሁን ከሰፈር ማለት ነው ጥዋት እኔ አንደኛ ክፍል ሁኜ ትምህርት ቤት ሲሄድ ጫት ሲራገፍ ነው የሚያየው የሄ ነገር ጥቅም አለው ማለት ነው ወይ የሆነ ነገር ይሮዋል ስው ዝም ብሎ እንትን አይልም በሚለው ኢንተረስተ /interest/ የሆነ ነገር ያድርባሃል ውስጥሕ ያ ደግሞ ህፃንነት እድሜ ላይ ስለሆንክ ያው የሆነ እንትን ውስጥ ይገባል /mind/ ውስጥ ይቀረፅበሃል እና የሆነች እድሜ ላይ ስትደረስ ያን ነገረ መጠቀም ትጀምራለህ ማት ነው

**P12: 2_M_FGD_Male user_BC .docx - 12:24 [????? ?? ??? ??? ?? /??? ???/ ..] (43:43) (Super)**

Codes: [Motivators for substance use- Previous exposure - Family: 3_Motivating factors for substance use]

No memos

አብዛኛው ግዜ የሱስ ላይፍ ወይ /የሱስ ሂወት/ የሚጀምረው በቃ ያችን ሃይስኩል ና መሰናድኦ ትምህርት ተጀምሮ ብዙ ሰው የሚጀምረው ከ10ኛ ክፍል በኃላ ከ9ኛ ክፍል በኃላ ያለው ነው እንዴት እንዴት ይጀምራል ለሚባለው በርግጥ እኔ የጀመሪኩት ገና ከ8ኛ ክፍል እንደጨረስኩ አከባቢ ነው ለዚህ አምስት አመት አሳልፋለሁ ማለት ነው ግን ሌሎቹ ጓደኞቼን ግን ሳያቸው ግን 10ኛ ክፍል ጨርሰው ማትሪክ እስከሚለቀቅ ክፍተት ባለው ዚኤ ጋፕ አልፋ ይሁን ወዲቄ ይሁን ሁሌም ጭንቀት አለ እና ያችን ሂወት ለማላለፍ ዳግም በቃ ልክ እንዳንተ ከተፈተኑት ወይ ደሞ ልክ እንደንተ ተፈትነው ከወደቁት ጋር ትውላላህ ልክ እንደዚህ አይነት ጊዜ ስታልፍ ከነሱ ጋር የሆነ ግዜ ታሳልፋለህ እባክህ ከወደቅክም ወደቅክ ነው ካለሆነ እንደዚህ እንደኛ እየሰራህ ታሳልፋለህ

**P12: 2_M_FGD_Male user_BC .docx - 12:25 [??? ??? ???? ????? ????? ?????..] (43:43) (Super)**

Codes: [Motivators for substance use- Previous exposure - Family: 3_Motivating factors for substance use]

No memos

መሞት የለም ካለህም ትኖራለህ ከሌለህም ትኖራለህ እየተባለ እንዲህ ትሰብካለህ ከዛም እዛ አካባቢ ላይ ማን እንዳሰገባህ ወይም ደግሞ እንዴት እንደገባህ ባላወቅከው ሁኔታ ራሰህን ሱስ ውስጥ ተዘፍቀህ ታገኘዋለህ ግን በምን ቅፅበት እንዲገባህ ምን አነሳሰቶህ እንዲገባህ ሪል ምክንያቱን አታቀውም ሩት ከውዘን አታቀውም ብቻ ግን ምክንያት ደርድር ብትበል ግን ዙሪያው ያሉት ምክንያቶች እነሱጋ መዋሌ ነው ውይም ደግሞ በወቅቱ የተወሰነ ብር ኪሴ ላይ ሰለ ነበር ያን ብር የሚጠፋበት ቦታ ሰለአጣሁኝ ወይም ጊዜየን የማሳለፍበት ቦታ አጥቼ ነው ብልህ ልታሰቀምጥትችላለህ ግን ሩት ከውዙን {Root cause} አታቀውም አንተ ማንም የሚያውቀው የለም ግን በነዚህ ምክንያቶች ነው ብልህ ልታስቀምጥ ትችላለህ

**P12: 2_M_FGD_Male user_BC .docx - 12:26 [?? ????? ?? ?????? ???? more ?..] (46:46) (Super)**

Codes: [Motivators for substance use- Previous exposure - Family: 3_Motivating factors for substance use]

No memos

እና ለመግባት ብዙ ምክንያቶች ይኖራሉ more ግዜ ሰታገኝና የምትውላቸው ሰዎች እኔ አሁን የጀመርኩት ከ 9ወደ 10 የክረምት ጥናት እየተማርኩኝ ነውና አንድ ወር ከምናምን ተምሬ የሆነ ልጅ አሰተማሪ ከሃላየ መጣና እንዳለ ከኃላ የምንቀመጠው ሁሉ እንባረራለን እንዳትመሩ ተብለን ማለት ነው በዛን ሰዓት ላይ እቤት ተባረሪኩ ብለህ መናገር ደሞ ይደብራል እቤት እሚከፍሉት እንድትማር ሰለ ሆነ ማለት ነው እና ሁላችንም እንመጣለን እንቀመጣለን የሆነ እኔ የደብረዘይት ልጅ ነኝ እና ሆራ እናጥና ብለን ጥናት እምናጠናው ደግሞ እዛ ቤዛ ት/ት ነውቤዛ ት/ትና ቤት ሆራ ዳም ፊት ለፊት ናቸው እዛ አልፎ ልትላይ የሰፈር ሰው ከየት እዛ እቤት ሄደ ይናገራል ስለዚህ እዛ እንገባለን ውስጥ ቁጭ እንላለን እሱ እንዳለው ሩቱን በምን እንደጀመሪኩም አላቅም እንዋኛለን ምናምን ነገር እዛ ወራ ማለት ነው እንወጣለን ከዛ በኃላ የሚጨሱ ልጆች ምናምን አሉ አብረውሽ የሚውሉት አብሮ የሚውለው ነው ዋናው የሚወስነው ነገር እጋዲ አሁን ተሰብስበን 2ት ሰው አይጨስም እንበል እነሱ የሚያጨሱ ከሆነ አንዳዴ ከነሱ ጋር ለመመሳሰልም ታረጋለህ ብቻ መጠጥም አሁን ብዙ ሰው ሳይፈልግ የሚያጨስ ሰው አለ በቃ እንደዚ ተሰብሰበህ እሱ ሲያጨሰ አይተህ ልታጨስ ትችላለህ አንተ እና እዛ ምክንያት ብዙ ሰው ከዛ 10ም ስንገባ በቃ ደይሊ (daliy) አላረግም እኔ ኖርማሊ ሲጋራ ነው ጫት ምናምን ግን ከ12ኛ በኃላ ነው በደንብ እንትን ያልኩት

**P14: 21_KIR 2_ 2nd interview_Busness comapas_Mekelle Univeristy.doc - 14:4 [?? ??? ?? ????? ??? ??? ???? ?..] (4:4) (Super)**

Codes: [Motivators for substance use- Previous exposure - Family: 3_Motivating factors for substance use]

No memos

ሌላ እዚህ ያሉ ተማሪዎች ደግሞ መቃም ነፃነት እንደማወጅ አድርጎ assume የሚደርጉት ብዙዎቹ ማለት ነዉ ዉጥረት አለባቸዉ ከቤተሰብ አለ ብዙ ነገር አለ ይህ አሁን በግቢ ዉስጥ ያሉ የሚገፋፋ ነገሮች አንዱ ነዉ እያነሳሁ ያለሁና አንጀመሩና በቃ በሱስ እንደጠመዱ ደርጋቸዋል

**P16: 23_KIR 2_1st interveiw_Busness campus Mekelle Univeristy.doc - 16:14 [Code 1 ??????? ?? ????? ????? ..] (128:128) (Super)**

Codes: [Motivators for substance use- Previous exposure - Family: 3_Motivating factors for substance use] [who use substnces: from Urban Vs Rural - Family: 6_Who are at risk of practicing subatnce use]

No memos

Code 1 እንደተበለው ቀድ ከየትኛው ቤተሰብና አከበቢ የመጣ ለሚለው አንዳኛ ነገር አንደኔ እንደኔ እኔ አሁን የመጠሁት ከደሴ ነው የውደሴ ከደር መዉጨጋ ሃይል አለች ከዘም ደግሞ ከሚሱ አለ ይው ዙር የዋን በጫት ተክል የተከበበች ነት አካበቢ ላይ ጫት ተካብይ፡፡

**P16: 23_KIR 2_1st interveiw_Busness campus Mekelle Univeristy.doc - 16:15 [???? ?? ????? ???? ??? ?? ?? ?..] (129:129) (Super)**

Codes: [Motivators for substance use- Previous exposure - Family: 3_Motivating factors for substance use]

No memos

የሚታይ ዕፅ አይደለም ስለዚህ ጠወሎ ልጅ ሆኖ ሀ ለጫት የመጥር አመለከበት የለው ሰው አይቦርም ስለዚህ እንድ የመጠንበት አከበቡ የወሰናል ስለዚህ ሁሉም ማለት የከብዳል ግን አብዘኛው ሰሜት የለው አስተያያት ጥሩ ነው በቃ ጫት ማድመቂያ ነው ጫት ማነቃቂያ ነው የሚለው ነው እንጂ ጫት መጥፍ ነው የዳነዝዛል የሚለውን ትርጉም በውስጡ ያየዘ ነገር የለውም በቃ እንደዚህ pcrcious (ፐርሊዮስ) (valuable) አመለካከት ነው የለው የወሎ ሰው

**P16: 23_KIR 2_1st interveiw_Busness campus Mekelle Univeristy.doc - 16:21 [Code.4. ????? ??? ?? ????? ???..] (131:131) (Super)**

Codes: [Motivators for substance use- Previous exposure - Family: 3_Motivating factors for substance use]

No memos

Code.4. በጠቃስዬ አሁን ሱስ በሚበለው አብዛኛው ሰው የሚዳምረው ሱስን እታችኛው ክፍል ላይ እየለ ነው የሄ ዳም ብዙ ትርፍ ጊዜ አለው በጠቀለይ እንደ ኢትዮጵያዊ ብዙ የማጥሰራው ነገር አለ የሚቀመጥበት ስዓት ይበልጠል የው ቀስ እየለ ሆቢ ሆኖበት ነው ከግ ወጪና ግቢ ውስጥ ማንምን የሚባለው ደም አሁን ማቐለ ውስጥ እንደለ ጨብሲ ቤት ነው በቃ በህል ነው ማለት ትችየለሽ ሌለም ቦታ ብትሄጂ ጫት በህል ነው ማለት ትችየለሽ እና በጠቃለይ ኢትዮጵያ በመሆኘሽ ከልጅነትሽ የሚየጅ ግብሽ ነገር ነው ማሰበው

**P17: 24_KIR 3_ Ayder campaus Mekelle Univeristy.docx - 17:4 [??? ??? ????? ??? ???? Back gr..] (18:18) (Super)**

Codes: [Motivators for substance use- Previous exposure - Family: 3_Motivating factors for substance use]

No memos

ደሽቲ ኣለዉ ክትሪኦም ከለካ መሊስካ Back grawond ዶሞ ብደንቢ ኣፅኒዖም ክትብሎም እንተለካ ልምዲ ነይርዎም ገና ዩኒቨርስቲ ከየኣቱ ልምዲ ዝነበሮም ኣብዙይ መፂኦም ከዓ ከም ብሓዱሽ ብብስጭት ኮነ ብካልእ ምክንያት ዝተወሰነ ልምዲ ስለዝነበሮም ተሎ ናብኡ develop ገይሮም ዝኣቱ እውን ኣለዉ፡

**P19: 4_IDI_ Male user BC.docx - 19:8 [??????? ???? ?????? Because ??..] (30:30) (Super)**

Codes: [Motivators for substance use- Previous exposure - Family: 3_Motivating factors for substance use]

No memos

የሚያወራሰው እንካን አይገኝምና Because ብሄርብሄረሰብ ነው እዚህ ያለው ከሃረ የመጣ ነው ከወለጋ የመጣ ነው ምናምንና እነሱ ደግሞ ብዙ ግዜ ከጋምቤላ የመጡ ናቸውና እነሱ ያለ ጫት ምንም actively አይቀሳቀሱም:: ስለዚህ ግዴታ ለማንበብ ሲሉ ይቆማሉና even ከቤተሰብ ራሱ ለጫት ተብሎ የራሳቸው ምድብ አለ ከዛ አከባቢ የመጡ እና ለማንበብ ይጠቀሙታል :: ሌሎቹ ግን በዕረት ሰዓት ምናምን የሚጠቀምወቸው ነገሮች አሉ::

**P20: 5_IDI_Male user-BC.docx - 20:8 [??? ????? ??? ?? ??? ???? ??? ..] (28:28) (Super)**

Codes: [Motivators for substance use- Previous exposure - Family: 3_Motivating factors for substance use]

No memos

ማለት አብዛኛው ተማሪ እዛ ጀምሮ የመጣም ሊሆን ይችላል ማለት በፊት 11ኛ 12ኛ እያለ

**P24: 9_IDI_Female proctor Ayder.docx - 24:29 [??! ??? ?? ???? ????? ??? ?? ?..] (99:99) (Super)**

Codes: [Motivators for substance use- Previous exposure - Family: 3_Motivating factors for substance use]

No memos

አዎ! አሁን ምን መሰለህ ተማሪዎች ማለት ነው በቃ አላማቸው ትምህርት ነው የመጡት ስለዚህ ደግሞ ሲመጣ ደህና ነበር፤ ሳይጀምር ነው የመጣው፤ ወንዶች ከሃይስኩል የሚጀምር አይጠፋም፤ ሴቶች ግን እዚህ ነው፤

______________________________________________________________________

**Code: Motivators for substance use- Undermining its consquences {2-0}**

**P 6: 14_X_FGD male non-user at Ayder docx.docx - 6:67 [??? ?? ????? ??? ????? ?? ?? ?..] (91:91) (Super)**

Codes: [Motivators for substance use- Undermining its consquences - Family: 3_Motivating factors for substance use]

No memos

የዛኔ ሱስ ይይዘኛል ብለህ ኣታስብል ወይ ዘና ለማለት ወይ ጓደኛ ለመምሰል እንደዛብለህ ከዛ ቀስበቀስ ወደሱስ ተጠቂ ትሆናለህ

**P10: 18_KIR 1_ 1st interview_Busness campus_mekelle univeristy.doc - 10:54 [I: ??? ?? ????? ?????? ????? ?..] (73:74) (Super)**

Codes: [Motivators for substance use- Undermining its consquences - Family: 3_Motivating factors for substance use]

No memos

*I:* ***ከሱስ ጋር የተያያዘ አጀማመርሽ ነግረሻል ወደ ሱስ የተቀየረብሽ መቼ ነው ትያለሽ ?***

***P:*** *ያው ከጓደኞቼ ጋር አብረን ነን፡፡ በቃ አለቀልን …(ሣቅ) እየተባባልን በቃ ሱሰኛ ሆንን እየተባባልን እንደቀልስ ነው የገባንበት፡፡*

______________________________________________________________________

**Code: Recommendations to adress motivating factors: Adminstartaive related {10-0}**

**P 1: 1_Female Users FGD in Busness college.docx - 1:85 [?? ????? ?????? ??? ???? ??? ?..] (108:108) (Super)**

Codes: [Recommendations to adress motivating factors: Adminstartaive related - Family: 3_Motivating factors for substance use]

No memos

እኔ የማስበዉ ዩኒቨርስቲ ይህን ዓይነት ሪስክ ኣለ።ኣሁን ዩኒቨርስቲ ስትገቢ ማንም ሰዉ ኣይቆጥርሽም።ከቤተሰቦችሽ ርቀሽ ነዉ ያለሽዉ።እና መንግስት ሃላፊነት ወስዶ ሲቀበልሽ ይህን ነገር ታሳቢ ቢያደርግ ጥሩ ነዉ።ጥሩ ነዉ ያለዉ ነገር ግን ለምሳሌ ያህል ዶርም ኣለ ዶርም እየወጣሽ ኣለማደር ግዴታ ቢሆን ብለሽ ታስቢያለሽ።ስድስት ሁኖዉ የሚታደርበት ዶርም ኣንድ ብቻዋን የምታድርበት ይኖራል።እየወጡ ሊጠጡ ሊጨፍሩ እና እንደዚህ ቢኖር ትላለህ።

**P 1: 1_Female Users FGD in Busness college.docx - 1:86 [???? ??? ?? ???? ??? ?? ??? ??..] (108:108) (Super)**

Codes: [Recommendations to adress motivating factors: Adminstartaive related - Family: 3_Motivating factors for substance use]

No memos

ላይፍሽ እዚህ ነዉ ምግብሽ እዚህ ነዉ ሁሉም ነገርሽ እዚህ ነዉ።እና ይህ ነገር ማድረግ ቢቻል የዚህ ቁጥር ይቀንሳል።መዉጣት ቢችል እና ቀን ቢያደርግ ቢያንስ የማታዉን ሰዓት ይጠቀምበታል፣ይተኛበታል፣ያርፍበታል፣ግን ቀን ክላስ ዉሎ ያጨሳል ሲጠጣ ያድራል።

**P 1: 1_Female Users FGD in Busness college.docx - 1:87 [???? ?? ?? ??? ??? ????????? ?..] (108:108) (Super)**

Codes: [Recommendations to adress motivating factors: Adminstartaive related - Family: 3_Motivating factors for substance use]

No memos

ስለዚህ ሰዉ ነን ዶርም መሆን የለበትም።ትልቁ የዩኒቨርስቲ side effect ወጥቶ ማደር እና ነን ዶርም ነዉ።

**P 1: 1_Female Users FGD in Busness college.docx - 1:88 [???? ???? ?? ??????? ?????? ??..] (109:109) (Super)**

Codes: [Recommendations to adress motivating factors: Adminstartaive related - Family: 3_Motivating factors for substance use]

No memos

መሰናዶ እያለሽ እኮ በስነስርዓት ትማርያለሽ ከዛ ቤት ትገብያለሽ ቤተሰብ ይቆጣጠርሻል ግን ይሄ ትንሽ ከፍ ማለት ኣለበት።በእርግጥ ራስሽን ነዉ በእራስሽ ማኔጅ የምታረጊዉ ግን ቢኖር ጥሩ ኔ ብዬ ኣስባለዉ።ቢያንስ ቁጥር ይቀንሳል

**P 1: 1_Female Users FGD in Busness college.docx - 1:106 [?? ???? ????????? ???? ????? ?..] (130:131) (Super)**

Codes: [Recommendations to adress motivating factors: Adminstartaive related - Family: 3_Motivating factors for substance use]

No memos

ግን እንደኔ እንደማየዉእንደ መፍትሄ ቢጠቀመዉ በብዬ የማስበዉ ማንም ተማሪ መዉጣት ባይችል።ፕሮክቶሮች ኣሉ ለምሳሌ በየቀኑ ሌሊት ሰዎችን ቆጥሮ ቢከታተል የተሻሉ ምሁራን ያፈራል እንጂ ኣይጎዳም።

በዩኒቨርስቲ ደረጃ ወጥቶ ማደር ባይፈቀድ ነዉ የምለዉ።ሌላዉ ነገር ኣሪፍ ነዉ።

**P 6: 14_X_FGD male non-user at Ayder docx.docx - 6:63 [????? ????? ??? ??? ???? ??? ?..] (85:85) (Super)**

Codes: [Motivators for substance use- helplessness - Family: 3_Motivating factors for substance use] [Recommendations to adress motivating factors: Adminstartaive related - Family: 3_Motivating factors for substance use]

No memos

ኣብዛኛው ተብሎዋል ትንሽ ለየት የምትል ነገር ኣላት ኣሁን የነዚህ ጋር የእነዚህ ምክንያት እና መንስኤ የሚሆነው የadministration የዚህ ግቢ የ administration ን የኣስተዳደር ለተማሪ ያላቸው ትኩረት ከኣስተማሪ ከላይኛውም ጀምሮ እስከታችኛው ያለ በቃ እዚህ ያለ ተማሪ ነገ ህዝብ ሀገር የሚረከቡ ህዝቡ የሚያገለግል እንደ ኣንድ ዜጋ ተቆጥሮ I donot know ምንም ለመግለፅ ኣትችልም እነሱ ማለት ኣብዛኛው በቃ የሆነ የራሳቸው ተብትነት ኣለ ሴትከሆነች የራስዋ የሴትነት ምታያይዝበት ወንድከሆነ የራሱ በቃ የየራሳቸው በቃ ኣንድ ቢሮ ሁነው የሆነህ ኣንድ ችግር ለመፍታት ኣይስማሙም በቃ ያኛው የራሱ ይይዛል ያኛውን እንደዛ ተማሪ በዚህ ይጉላላል ለኣንዲት የሆነች ችግር ለመፍታት ሁለት ኣመት መጣም የሚያሳዝን ማለት ነው

**P 7: 15_X_IDI_ Male proctor Ayder.docx - 7:46 [?? ??? ??? ???? ?? ?? ?? ???? ..] (84:84) (Super)**

Codes: [Recommendations to adress motivating factors: Adminstartaive related - Family: 3_Motivating factors for substance use]

No memos

አዎ በዚህ ትንሽ ልጨምር ዝም ብሎ ዋላ መፍትሄ ይሆናል ባይባልም እንደ እኔ መጠን ምን አለ መሰለህ አሁን የዩኒቨርሲቲው ሕገ ደንብ፤ ሲጋራ፣ ጫት ይዞ መገኘት አይገባም ጠጥቶ መግባት አይችልም ይባላል፤ ሲባል ግን ተማሪ ጠጥቶ መግባት አይችልም ይባላል፤ ሲባል ግን ተማሪ ጠጥቶ ወይም ይዞ ይመጣል፤ ሰራተኛ ደግሞ ወደ የሚመለከተው ያሳልፋል በህገ ደንቡ መሰረት ይህንን ሰው ሰው ጫት ይዞ እየቃመ አግኝቸዋለሁ፡፡ ግቢ ውስጥ ሲጋራ እያጨሰ ወይም ሰክሮ ገብቷል ስትል ጊዜ ተቀባይነት የለህም ማለት ይቻላል። በሌላ አባባል ምን ማለት ነው ህግ ጠንክሮ የማይል ከሆነ ተማሪም ማን ተቀጣና እያለ ይጠቀማል እንደተፈቀደ አድርጎ ነው የሚወስደው ዋና አስቸጋሪ ነገር እርምጃ አለመወሰድ ማለት ይባሀ ማለት አይደለም እርምጃ ሲባል ብዙ ዓይነት ነው።

**P 7: 15_X_IDI_ Male proctor Ayder.docx - 7:47 [???? ??? ?? ???? ??? ??? ?????..] (84:84) (Super)**

Codes: [Recommendations to adress motivating factors: Adminstartaive related - Family: 3_Motivating factors for substance use]

No memos

እርምጃ ሲባል ብዙ ዓይነት ነው። ምክር መስጠት፤ ማስጠንቀቂያ መስጠት፤ ወደ ሌላ ከዛ በኋላ ይህን ሁሉ ካልተደረገ እኛ እናሳልፋለንና ምን እንደርጋለን ሰለቸን አሁን ሲጋራ የሚያባርር መሆን ካወቅኩኝ በምንም ታአምር እልሞክረውም፤ ሕገ-ደንቡ ጠንከር ማለት አለበት አፈፃፀም ላይ፡፡

**P14: 21_KIR 2_ 2nd interview_Busness comapas_Mekelle Univeristy.doc - 14:58 [Fresh ?? ?? ??? ???? ???? ??? ..] (49:49) (Super)**

Codes: [Recommendations to adress motivating factors: Adminstartaive related - Family: 3_Motivating factors for substance use]

No memos

Fresh ላይ በኛ ሰዓት አምሽቶ የሚገባ ተማሪ የለም Attendance ካልፈረመ ሶስት አራት ቀን / Attendance ከቀረ በወር ዉስጥ ሂዶ ማስጠንቀቂያ ይፈርማል ከዛ በኃላ ከቀረ ይባረራል ተብሎ ተጀምሮ ነበር Fresh ላይ እያለን ያን ነገር ሁለት ወር ሶስት ወር ሳይቀሩ ቀራ ነገሩ ፊርማ ማለት ነዉ፡፡ ያ መቅረቱ በራሱ የራሱ ተፅዕኖ አለዉ በሱ ተጠመደዉ የነበሩ ተማሮዎች ሰዓት አምሽተዉ እንዲገቡ በጣን ብዙ ግዜ ላይ ግዜያቸዉ እንዲያሳልፉ በራሱ ተፅዕኖ አለዉ ማለት ነዉ፡፡

**P14: 21_KIR 2_ 2nd interview_Busness comapas_Mekelle Univeristy.doc - 14:59 [?? ??? ????? Ø ??? ??? ????? ?..] (50:51) (Super)**

Codes: [Recommendations to adress motivating factors: Adminstartaive related - Family: 3_Motivating factors for substance use]

No memos

ጥሩ ነበር ፊርማዉ?

⮚ በጣም ነበር ተማሪዎች ተያይዘዘዋል በቃ 4፡00 ሰዓት ላይ የግደ ማገኘት አለበት እዛ ቦታ ላይ 4፡00 ሰዓት ላይ እዛ ተገኘ ማለት ከ4 ሰዓት በኃላ ተመልሶ አይመጣም፡፡ ሌላ እንደዚህ እዚህ ሆኖ የቤተሰብ ተፅዕኖ ሊኖርበት ይችላል፡፡

______________________________________________________________________

**Code: Recommendations to adress motivating factors: awearness creation {2-0}**

**P 8: 16_FGD ARID_Users .docx - 8:53 [?? ?????? ??? ??? ??? ??? ????..] (94:95) (Super)**

Codes: [Recommendations to adress motivating factors: awearness creation - Family: 3_Motivating factors for substance use]

No memos

ጤና አገልግሎት በግቢ ውስጥ የሉት ሰዎች አንኳን ለሱሰኞች ለሌሎች አንኳን በቂ እውቀት ያለቸው አይመሰለኝም ፡፡

ብዙ እውቀቱ የለኝም ሽሻ የሚበለው ነገር ጥሩ መኣዛ አለው አብዘኛዉም ግዜ

**P17: 24_KIR 3_ Ayder campaus Mekelle Univeristy.docx - 17:14 [????? ????? ????? ???? ?????? ..] (32:32) (Super)**

Codes: [Recommendations to adress motivating factors: awearness creation - Family: 3_Motivating factors for substance use]

No memos

እንድሕር ተመኪርካ ተቐፂዕካ ብደንቢ እነተዓቢካ ያው ኣብ ፅቡቅ ደረጃ ብፅቡቅ መንገዲ ክትከይድ ትክእል ኢካ እድሕር ዳኣ ከምድሌትካ ብወነንካ ትከይድ እንተኮይንካ ኩሉ ግዜ ግን ትከዶ መንገዲ ዝተጋገየ ክከውን ስለዝክእል መጀመሪያ ኣብቲ family ውን ኣብቲ እቶም ደቆም ዘዕብዩ ኣካላት ኣብዚኦም ውንስ ክስርሖ ዝግባእ ነገር ከምዘሎ እየ ዝርዳእ ማለት እዩ ምስ ገንዘብ ተታሓሒዙ፡፡

______________________________________________________________________

**Code: Recommendations to adress motivating factors: Limit access of the substances {2-0}**

**P 9: 17_Male User_Adihaki campus.docx - 9:21 [?? ???? ??? ??? ??? ??? ??? ??..] (48:49) (Super)**

Codes: [Recommendations to adress motivating factors: Limit access of the substances - Family: 3_Motivating factors for substance use] [Recommendations to help ceasing: provide alternative enatrtainment - Family: 8_Intention to cease and experiance of relapse for substance use]

No memos

ይህ አከባቢ ለሱስ ቅርብ የሆኑ ነገሮ ሱቆች ጫት ቤቶች ለሌለ ቢዝነስ ቢቀየር በግቢ ውስጥ መዝናኛ ነገር ቢኖር ጥሩ ነው

ምሰሌ የሚነበብበት ቦታ /መፅሃፍ ማቆርብ ) የሚወያይበት ቦታ

**P11: 19_KIR 1_ 2nd Ineterbview_ busness compus_Mekelle University.docx - 11:25 [?? ??? ?? ????? ???? ??? ??? ?..] (42:42) (Super)**

Codes: [Recommendations to adress motivating factors: Limit access of the substances - Family: 3_Motivating factors for substance use]

No memos

አው አሁን እኔ በእንትን እንትን ምለው አሁን እዚ አካባቢ ማለት ነው፡፡ አሁን የተከፈቱ አሉ ሲጀመር መጠጥ ቤቶች እና ተጨማሪ መጠጥ ቤት እንዲከፈት እንኳን ገደብ ምናምን ቢኖር ጥሩ ነበር ማት ከ እንትን አንፃር የሀ የክልሉ ምናምን ማለት ነው፡፡ ማለት ዩኒቨርሲቲ አካባቢ መክፈት ኢኮ ክልክል ነው ግን እዚው አቅራቢያ አሁን ራሱ የእየሲሱ ነው፡፡ ልክ ስንመጣ First semester ላይ ኖቪል ፕላልቱ ተከፈተ አዲስ አሁን ደግሞ በዛ በኩል ሁለት ሌላ ተከፈቱ እነዚ ነገሮች ይልጥ ነገሩን እያደረሱ ነው ማለት የመጠጥ ከተማ እየሆነች ነው አንቺ ቦታ ራሱ ማት ነው፡፡

______________________________________________________________________

**Code: Recommendations to adress motivating factors: Public Discussions {2-0}**

**P11: 19_KIR 1_ 2nd Ineterbview_ busness compus_Mekelle University.docx - 11:21 [?? ????? ???? ??? ????? ??? ??..] (33:33) (Super)**

Codes: [Recommendations to adress motivating factors: Public Discussions - Family: 3_Motivating factors for substance use]

No memos

እኔ ከእንደዚ ዓይነት ማለት ውይይቶች ቢኖሩ ብየ አስባለው፡፡ ማለት አ እንደዚ አሁን ለምሳሌ public lecture ዓይነቶች ተማሪ እንድገባ ምናምን ተደርጎ በእንደዚ ዓይነት ትምርቶች ምናምን ቢሰጡ እነዛ ልጆችን ማግኘት ይቻላ፡፡ ማት አሁን ለምሳሌ እኔ እንኳን ከተረዳሁት ኑ አውሩ ስንላቸው ባለፈው አብዛኛውን ደስ አላለውም፡፡ ማት ሁሉም አልፈለገም፡፡

**P11: 19_KIR 1_ 2nd Ineterbview_ busness compus_Mekelle University.docx - 11:22 [????? ?? ?????? ?????? ??? ?? ..] (33:33) (Super)**

Codes: [Recommendations to adress motivating factors: Public Discussions - Family: 3_Motivating factors for substance use] [Recommendations to help ceasing: Peer support - Family: 8_Intention to cease and experiance of relapse for substance use]

No memos

የባለፈው እኛ የተወያየን ያልባቸውን ምናም በቃ እንባቸው መጥቶ ምናምን እየተናገሩ ነበር፡፡ ግን አሁን ያየሆነው ብዙ የሀ ውይይቱ ከመስፋቱ የተነሳ ነው፡፡ ልክ እንደዛ ዓይነት ውይይቶች ቢኖሩ ማለት ውስጣቸው ማግነት ይቻላል፡፡ ግን ከላይ ከላይ የሆነ ነገር ከላይ ከላይ መሆኑ ስለሚታወቅ ማንም አይገባበትም ማለት ነው፡፡

______________________________________________________________________
